# Design and Optimization of Spiro-Isatin-Thiazolidinone Hybrids with Promising Anticancer Activity

**DOI:** 10.3390/ph18101502

**Published:** 2025-10-07

**Authors:** Dmytro Khylyuk, Serhii Holota, Natalia Finiuk, Rostyslav Stoika, Tetyana Rumynska, Roman Lesyk

**Affiliations:** 1 Chair and Department of Organic Chemistry, Medical University of Lublin, ul. Chodźki 4a, 20-093 Lublin, Poland; 2 Department of Pharmaceutical, Organic and Bioorganic Chemistry, Danylo Halytsky Lviv National Medical University, 69 Pekarska St., 79010 Lviv, Ukraine; golota_serg@yahoo.com; 3 Department of Organic and Pharmaceutical Chemistry, Lesya Ukrainka Volyn National University, 13 Volya Ave., 43025 Lutsk, Ukraine; 4 Institute of Cell Biology of National Academy of Sciences of Ukraine, 14/16 Drahomanov Str., 79005 Lviv, Ukraine; nataliyafiniuk@gmail.com (N.F.);; 5 Department of Microbiology, Danylo Halytsky Lviv National Medical University, 69 Pekarska St., 79010 Lviv, Ukraine; tanityshka.r@gmail.com; 6 Department of Biotechnology and Cell Biology, Medical College, University of Information Technology and Management in Rzeszow, Sucharskiego 2, 35-225 Rzeszow, Poland

**Keywords:** spiro-isatin-thiazolidinone hybrids, MDM2–p53 interaction, molecular docking, drug design

## Abstract

**Background:** Cancer remains a leading cause of morbidity and mortality worldwide, and current therapies are limited by toxicity, cost, and resistance. Inhibition of the MDM2–p53 interaction is a promising anticancer strategy, as this pathway is frequently dysregulated across tumors. Spiro-isatin-thiazolidinone derivatives have shown diverse biological activities, including anticancer effects, but require optimization to improve potency and selectivity. The aims were to design, synthesize, and evaluate novel spiro-isatin-thiazolidinone hybrids with enhanced cytotoxicity against cancer cells and reduced toxicity toward normal cells. **Methods:** Derivatives were designed using molecular docking against MDM2, followed by structural optimization. Cytotoxic activity was evaluated in vitro by MTT assays on human and murine cancer cell lines and pseudo-normal cells. Docking and 100 ns molecular dynamics simulations assessed binding stability, while ADMET properties were predicted in silico. **Results:** Several derivatives exhibited micromolar cytotoxicity, with compound **18** emerging as the most potent and selective candidate (IC_50_ 6.67–8.37 µM across most cancer lines; >100 µM in HaCaT). Docking showed a strong affinity for MDM2 (−10.16 kcal/mol), comparable to the reference ligand, and stable interactions in simulations. ADMET predictions confirmed good oral bioavailability and moderate acute toxicity, fully compliant with Lipinski’s Rule of Five. Overall, the newly synthesized spiro-isatin-thiazolidinone hybrids, particularly compound **18**, demonstrated potent and selective anticancer activity, favorable pharmacokinetic properties and a good toxicity profile.

## 1. Introduction

Cancer poses significant challenges to global healthcare systems. In the search for novel anticancer agents, researchers face multiple obstacles, including the multifactorial nature of cancer, the adverse effects of conventional treatments, and the high production costs of innovative therapies [1,2,3].

The development of effective anticancer agents relies on the identification of appropriate molecular targets. Currently, nearly 200 potential targets have been identified for anticancer therapy. However, this number is likely incomplete, highlighting the complexity of cancer as a polyfactorial disease [4]. The multifaceted nature of cancer, which involves numerous genetic, epigenetic, and environmental factors, complicates the process of developing targeted therapies that can comprehensively address the diverse mechanisms driving tumor progression [5].

Anticancer treatments are often associated with significant adverse effects, which can lead to treatment discontinuation and may compromise otherwise healthy physiological systems [6,7]. The challenge of balancing therapeutic efficacy with patient safety remains a critical issue in oncology. While newer treatments may improve survival rates, the potential for severe side effects underscores the need for therapies that maximize clinical benefit while minimizing harm to the patient [8,9].

The production of cutting-edge anticancer therapies, such as monoclonal antibodies (e.g., pembrolizumab Keytruda^®^), poses significant economic challenges [10]. Despite their promising efficacy in tumor management, the high cost of manufacturing these therapies limits their accessibility, particularly in resource-limited healthcare systems [11].

Developing small-molecule-based new anticancer agents with broad efficacy across various cancer cell lines and utilizing low-cost production methods is an urgent priority for medicinal chemists, governments, and the pharmaceutical industry [12,13]. Achieving this goal is essential to improving accessibility and advancing global cancer treatment [14,15].

One of the main attractive targets for the development of new anticancer agents with potent, broad-spectrum cytotoxicity against various types of cancer cells is the inhibition of the MDM2–p53 interaction [16,17,18]. The p53 protein, often referred to as the “guardian of the genome,” plays a critical role in regulating cell cycle arrest and apoptosis [19,20]. The MDM2 protein regulates p53 by maintaining its low levels in normal cells, thereby preventing unnecessary apoptosis. Mutations in the *TP53* gene are implicated in approximately 50% of all human cancers, making it a significant factor in the development of tumors [21,22]. Additionally, p53 mutations are one of the main genetic alterations that provoke breast cancer. However, even in cancers where the *TP53* gene remains wild-type, p53 functionality can be compromised through alternative regulatory mechanisms, such as overexpression of MDM2 or other inhibitory factors [23,24].

Recent advances have led to the development of synthetic small-molecule inhibitors that specifically target a hydrophobic pocket on MDM2, the site where p53 normally binds. These inhibitors, known as MDM2–p53 antagonists, are currently undergoing clinical trials for a variety of cancer types [25,26]. Despite their promise, no MDM2 inhibitors have yet been approved for market use [27].

Many of these investigational inhibitors include spiro derivatives of the isatin core [28,29,30]. Additionally, 4-thiazolidinone-spiro indole-based compounds exhibit a wide range of biological activities, including anticancer [31], antibacterial, antitubercular [32], and anti-inflammatory effects [33]. Among these, certain 4-thiazolidinone-spiro indole compounds have demonstrated notable anticancer activity. However, detailed studies on the mechanism of action of Les-3467, a compound within this class, revealed non-selective effects on both cancerous and pseudo-normal cells, particularly at higher concentrations. Les-3467 differentially regulates p53 expression, increasing its levels in normal BJ cells while decreasing it in A549 lung cancer cells, suggesting a potential interaction with the MDM2–p53 axis. The upregulation of p53 in BJ cells may indicate MDM2 inhibition, leading to P53 stabilization, while its downregulation in A549 cells suggests either enhanced MDM2 activity or alternative degradation mechanisms. The compound also induces oxidative stress and apoptosis, which may contribute to its effects on p53 regulation. Furthermore, experimental data suggest that Les-3467 also acts as a PPARγ modulator, implicating its involvement in additional biological pathways beyond its anticancer activity [34].

**Figure 1:**
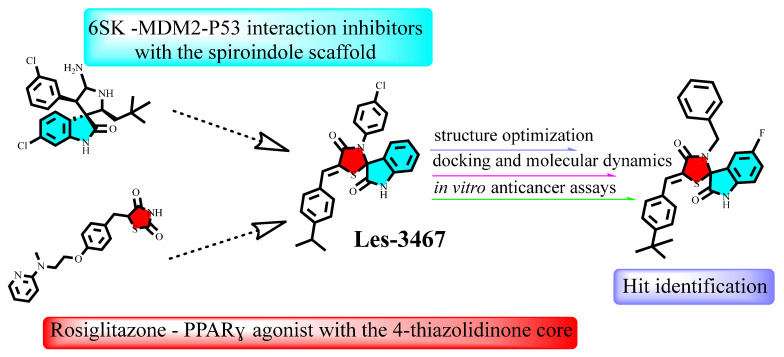
Structural optimization of the Les-3467 compound to enhance its anticancer activity and increase the selectivity of its cytotoxic effect.

Considering the potential interactions with MDM2 and partial agonism of PPARγ, the activation of which may also exert a pronounced anticancer effect [35,36], spiro 4-thiazolidinone–isatin derivatives represent an intriguing class of hybrid anticancer compounds with unique and complex mechanisms of action. However, their selectivity for cancer cells and toxicity profile need to be improved based on the available literature data and modern drug design approaches, such as docking and molecular dynamics techniques. To address these challenges, new molecules have been designed to enhance their anticancer activity and reduce toxicity profiles (Figure 1).

## 2. Results

### 2.1. The Rationale of the Synthesis

To identify potential structural modifications of the molecules, docking studies were conducted for Les-3390, Les-3470 with MDM2 (PDB 5LAV) and PPARγ (PDB 5YCP). The obtained AutoDock Vina docking scores are presented in Table 1. The binding energies of the native ligands (6SK from 5LAV and BRL from 5YCP) were used as a standard for comparison with those of Les-3470 and the proposed compounds. Docking simulations revealed that while Les-3390 possesses the oxospiroindole core and forms key interactions with crucial residues, such as two hydrogen bonds with His96 and Leu54, anchoring the inhibitor to the protein, it is not optimally suited to fully occupy the hydrophobic cavities of MDM2. Specifically: (A) Suboptimal Occupation of the Trp23 Pocket: The isatin scaffold does not fully fill the Trp23 pocket, resulting in weaker hydrophobic interactions. (B) Insufficient Fit in the Leu26 Pocket: The 4-chlorophenyl core of Les-3390 is poorly suited to the Leu26 pocket, leading to suboptimal π–π stacking or hydrophobic contacts that are critical for strong binding. The absence of additional interaction points is clear—both the reference ligand and the parent compound Les-3390 possess three interaction points. Introducing additional interaction points may enhance the affinity of novel derivatives for MDM2.

**Figure 2:**
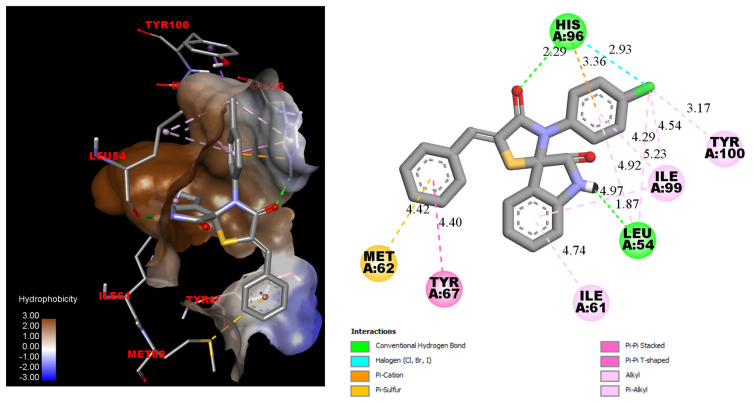
AutoDock Vina predicted pose of the Les-3390 with the MDM2 (PDB 5LAV).

In summary, while Les-3390 establishes fundamental hydrogen bonds with His96 and Leu54, its scaffold and substituents do not fully exploit the hydrophobic cavities of MDM2. This limits its binding affinity and efficacy compared to better-optimized inhibitors from the spiro indole derivatives, which provide superior pocket occupancy and additional stabilizing interactions. Furthermore, comparing the binding energy and inhibition constant reveals a significant difference between the reference molecule and Les-3390. The obtained in silico simulation data suggest potential pathways for optimizing 4-thiazolidinone and isatin derivatives (Figure 2).

Regarding the activity towards PPARγ receptors, the docking studies did not demonstrate significant binding energy or interactions with the key amino acid residues (His323, Ser289, His449, Tyr473) that stabilize the ligand, nor with Ser245 in the H2-β1 loop, which is crucial for inhibiting Cdk5-mediated phosphorylation [37].

Despite the obtained binding energies, the mechanism of PPARγ activation remains a controversial issue that requires further in-depth research. However, considering the experimentally confirmed activation of these receptors by the studied class of compounds, even with significant binding energy that does not reach the levels of reference compounds, the possibility of partial or indirect activation of PPARγ should be considered. Consequently, this may, to some extent, contribute to the expression of antitumor activity through this mechanism.

Considering the results of the docking simulation, the next steps in the optimization of the 4-thiazolidinone-isatin hybrids are outlined as follows:

(a) Addition of a Halogen at the 5-position of the Isatin Ring: Adding a halogen (e.g., chlorine or fluorine) at the 5-position of the isatin ring is expected to improve the fit within the binding site. This modification can enhance binding through halogen bonding or hydrophobic interactions, contributing to better pocket occupancy and additional stabilization.
(b) Replacement of the 4-Chlorophenyl Core with a Benzyl Substituent: Substituting the rigid 4-chlorophenyl core with a more flexible benzyl group could allow better suiting of the molecule to the binding pocket. This flexibility may lead to stronger hydrophobic interactions and better alignment with key residues, enhancing overall binding affinity.
(c) Addition of a Substituent to the 5-Arylidene Portion of the Molecule: Incorporating a substituent (e.g., or polar group) on the 5-arylidene moiety could improve binding energy by introducing new interactions with residues in the binding site. This change could also stabilize the ligand’s position more effectively, ensuring a stronger and more specific fit.

Such optimizations may enhance selectivity for normal cells by increasing cytotoxic activity against targeted cell lines or by reducing the overall toxic profile.

### 2.2. Synthesis

**Figure 3:**
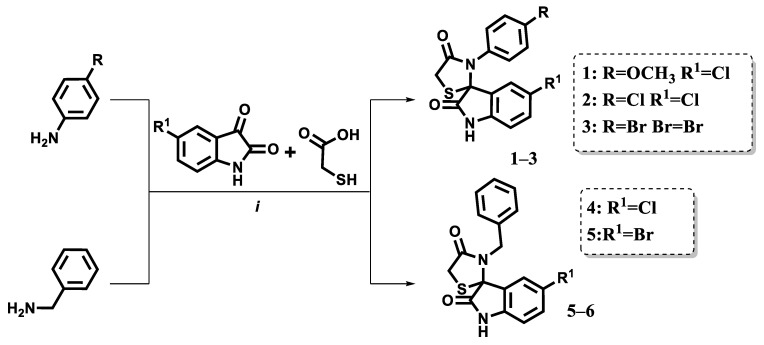
Synthetic Pathway of Spiro Thiazolidinone-Isatin Conjugates (i—anhydrous toluene medium under reflux with a drop of acetic acid for 6 h, followed by treatment with mercaptoacetic acid under reflux for 24 h).

The compounds were synthesized following a previously described procedure. Briefly, equimolar amounts of isatin and the corresponding aromatic amines were heated in anhydrous toluene with the addition of a few drops of acetic acid. After one hour of heating, a threefold excess of thioglycolic acid was introduced (Figure 3). The resulting intermediates then underwent a Knoevenagel condensation with aromatic aldehydes.

**Figure 4:**
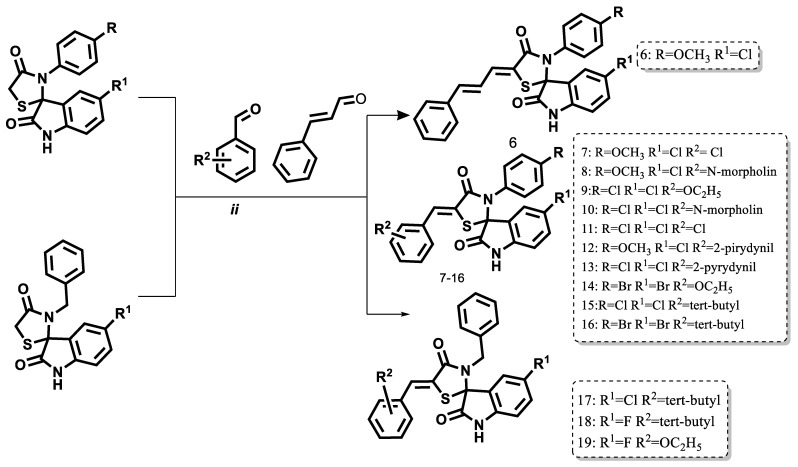
Scheme of the synthesis of 5-arylidene derivatives from spiro-thiazolidinone–isatin conjugates. (ii—heating in isopropanol for 3 h with an excess of the corresponding aromatic aldehyde and potassium tert-butoxide as a basic catalyst.).

Due to the enhanced reactivity of the methylene group at the 5-position of the 4-thiazolidinone ring, a strong base-catalyzed Knoevenagel reaction was employed, utilizing potassium tert-butoxide as the catalyst. The final compounds were purified by crystallization from a preselected solvent (Figure 4).

The structures of the derivatives **1**–**19** were confirmed by the ^1^H-NMR and ^13^C-NMR (copies of the corresponding spectra are presented in the Supplementary Materials).

### 2.3. Anticancer Activity

The cytotoxic potential of the synthesized compounds was investigated using the MTT assay after 72 h incubation with a panel of human and murine cancer cell lines, including colorectal carcinoma (HCT116), estrogen receptor-positive breast adenocarcinoma (MCF-7), triple-negative breast adenocarcinoma (MDA-MB-231), epidermoid carcinoma (KB3-1), myeloid leukemia (K562), and glioblastoma (U373), and mouse breast carcinoma (4T1) cells. To assess the selectivity of the compounds, pseudo-normal cell lines—human keratinocytes (HaCaTs) and mouse fibroblasts (NIH3T3s)—were also tested under the same conditions. IC_50_ values were determined for all compounds and are summarized in Table 2.

Among the tested series, compound **18** emerged as the most potent and broadly active molecule, demonstrating notable cytotoxicity across all examined cancer cell lines. The IC_50_ values for compound **18** ranged from low micromolar to submicromolar levels: 8.37 µM (HCT116), 6.99 µM (MCF-7), 6.67 µM (MDA-MB-231), 7.92 µM (KB3-1), 37.37 µM (K562), and 29.81 µM (U373). Importantly, its activity against pseudo-normal HaCaT and NIH3T3 cells was significantly lower (IC_50_ > 100 µM and 98.39 µM, respectively), indicating selectivity for cancerous over normal cells.

This selective cytotoxicity is especially relevant when compared to the reference drug doxorubicin, which exhibited potent anticancer activity but also considerable toxicity toward normal cells. While doxorubicin showed lower IC_50_ values in certain cancer lines, its activity against HaCaT and NIH3T3 was also high, underlining its narrow therapeutic window. In contrast, compound **18** demonstrated a more favorable balance between efficacy and safety, suggesting its advantage in terms of therapeutic index.

Structurally related compounds **16** and **17** also exhibited promising cytotoxic profiles. Compound **17** was active against HCT116 (8.24 µM), MCF-7 (6.64 µM), MDA-MB-231 (13.52 µM), and KB3-1 (5.85 µM) and possessed lower toxicity towards U373 (32.26 µM) and K562 (67.86 µM), while compound **16** showed lower potency against KB3-1 (29.47 µM) and MDA-MB-231 (53.27 µM), with moderate activity in other lines. However, the IC_50_ of compound **17** against pseudo-normal cells (e.g., HaCaTs) was lower than that observed for compound **18**, indicating reduced selectivity.

The broad activity profile of compound **18**, coupled with its favorable selectivity, suggests a potential mechanism involving the modulation of fundamental regulatory pathways in cancer cells. One such mechanism could be the reactivation of p53 tumor suppressor function via inhibition of the MDM2–p53 interaction, as this pathway is commonly dysregulated across various malignancies. The scaffold of compound **18** contains structural features consistent with known allosteric modulators of MDM2, supporting this hypothesis. However, further mechanistic studies—including molecular docking, target engagement assays, and apoptosis markers—are needed to confirm this proposed mode of action.

In contrast, several compounds in the series, such as **1**, **4**, **8**, **9**, **14**, and **15**, did not exhibit relevant cytotoxicity (IC_50_ > 100 µM for most cancer lines) and can be considered inactive under the tested conditions.

Taken together, the data strongly support compound **18** as the lead molecule in this series. Its potent, broad-spectrum cytotoxicity, combined with low toxicity toward normal cells, highlights its promise as a candidate for further development. Follow-up studies will focus on validating its molecular targets, optimizing its pharmacokinetic properties, and evaluating its in vivo anticancer efficacy.

### 2.4. In Silico Simulation

#### 2.4.1. Docking

Docking results indicate that the tested set of compounds exhibits a higher affinity for MDM2 in most cases, demonstrating binding energies comparable to or even exceeding those of the reference ligand Nutlin-3a (Table 3). Notably, compound **18** exhibited a binding energy comparable to that of the reference ligand, 6SK. The docking results were broadly consistent with the cytotoxicity profiles observed across the panel of tested cancer cell lines. Incorporation of an arylidene moiety introduced additional interaction sites within the binding pocket and yielded a more favorable binding energy for the resulting complexes. Regarding affinity for PPARγ receptors, most compounds exhibit significantly lower binding energies compared to the reference ligand Lobeglitazone. However, compound **18**, which demonstrated the highest cytotoxic activity, also showed a relatively high binding affinity for PPARγ. Nevertheless, analysis of the compound–receptor complex with the best docking score suggests that the compound does not interact with the key amino acid residues required for receptor activation. Partial interaction may still occur, potentially contributing to the overall anticancer effect through indirect modulation of PPARγ activity.

Compound **18** forms two hydrogen bonds within the MDM2 binding site: one with His96 (1.78 Å) and another with Gly58 (2.76 Å) via the fluorine atom of the 5-fluoroisatine core. Additionally, the same fluorine atom establishes two non-covalent halogen bonds with Leu54 and Phe55, contributing to enhanced binding stability. This strong anchoring effect improves the overall binding affinity compared to unsubstituted spiro-isatin derivatives, which lack these specific interactions.

**Figure 5:**
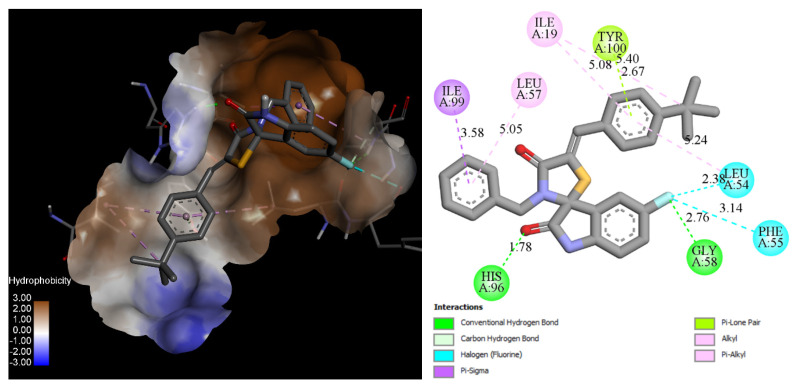
Three-dimensional and two-dimensional schemes of the interaction of the compound **18** in the best docking pose inside MDM2 (PDB 5LAV).

Furthermore, due to its increased flexibility and longer distance, the benzyl substituent better accommodates the cavity formed by Ile99 and Leu57, optimizing ligand fit within the binding site. The 4-tert-butylphenylidene moiety interacts with Ile19 and Tyr100 through alkyl and π–lone pair interactions, further stabilizing the ligand–receptor complex (Figure 5).

#### 2.4.2. Ligand Stability in the MDM2 Binding Pocket: 100 ns MD Insights

**Figure 6:**
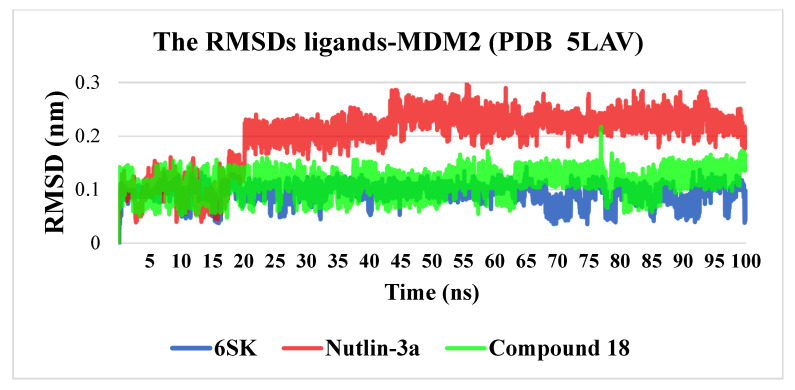
RMSD dynamics of ligands (6SK, Nutlin-3a, and Compound **18**) in complex with MDM2 (PDB ID: 5LAV) during a 100 ns molecular dynamics simulation.

Over the 100 ns molecular-dynamics trajectories, heavy-atom RMSD profiles clearly discriminated the conformational stability of the three ligand–MDM2 complexes (Figure 6).

- 6SK underwent a brief relaxation (~5 ns), after which its RMSD plateaued in the 0.02–0.12 nm range, implying a rigid, tightly anchored pose. The ligand remains locked in the hydrophobic cleft through a persistent π-stacking/van der Waals triad with Trp23, Leu54 and Leu57.
- Compound **18** showed a gradual rise in RMSD to ≈0.18 nm, indicating modest flexibility: its core contact pattern is retained while peripheral substituents periodically re-orient, suggesting scope for scaffold refinement without sacrificing affinity.
- Nutlin-3a displayed the largest excursions (0.18–0.28 nm) for most of the trajectory, reflecting partial displacement from the optimal binding pose and intermittent loss of key hydrogen bonds—behavior consistent with the higher dissociation constants reported for this chemotype.

Collectively, 6SK emerges as the most promising inhibitor: its low dynamics point to a highly favorable interaction energy and potentially slow off-rate. Compound **18** maintains intermediate potential for optimization, whereas Nutlin-3a would require substantial modification to achieve comparable complex stability.

**Figure 7:**
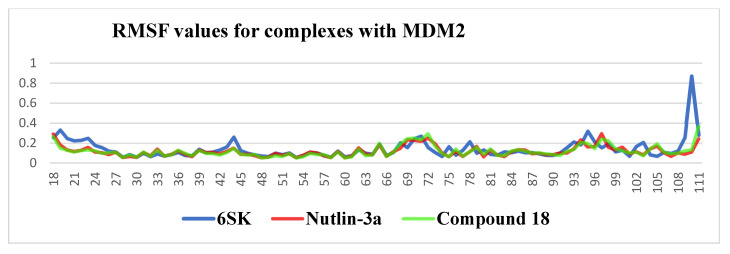
RMSF values of MDM2 residues in complexes with ligands 6SK, Nutlin-3a, and Compound **18** over the 100 ns molecular dynamics simulation.

After aligning each trajectory to the initial backbone reference, per-residue Cα root-mean-square fluctuations (RMSFs) were computed for residues 18–111 to quantify local flexibility induced by the three ligands. Across the structured core of MDM2 (residues 30–100) all RMSF values remained below 0.30 nm, confirming that ligand binding preserves the global integrity of the scaffold; however, distinct dynamic signatures emerged at several peripheral segments. The 6SK complex displayed three pronounced mobility hotspots: a solvent-exposed loop at residues 40–45 (RMSF ≈ 0.25 nm), the tip of the “lid” helix α4 at residues 67–75 (≈0.26 nm) and, most strikingly, the disordered C-terminal tail at residues 108–111, which surged to ≈0.85 nm. Nutlin-3a damped the N-terminus and lid comparably to Compound **18** but only partially restrained the tail, yielding intermediate fluctuations (~0.45 nm). Compound **18** imposed the tightest global control: outside the inherently mobile terminal residues its RMSF seldom exceeded 0.20 nm, indicating that its contact network transmits stabilizing constraints well beyond the immediate binding cleft. Collectively, these data suggest that although 6SK locks firmly into the hydrophobic pocket (as evidenced by its low ligand RMSD reported earlier), it transmits less mechanical restraint to distal loops and the C-terminus than Compound **18**, potentially translating into a faster off-rate. Conversely, the extensive damping exerted by Compound **18** supports a longer residence time despite its slightly higher ligand flexibility, while Nutlin-3a occupies an intermediate dynamic regime. These insights highlight the value of coupling ligand-centric and protein-centric metrics in inhibitor optimization and point to strategic positions—particularly around residues 40–45 and 108–111—where additional interactions could merge the high-affinity pose of 6SK with the conformational control exhibited by Compound **18** (Figure 7).

**Figure 8:**
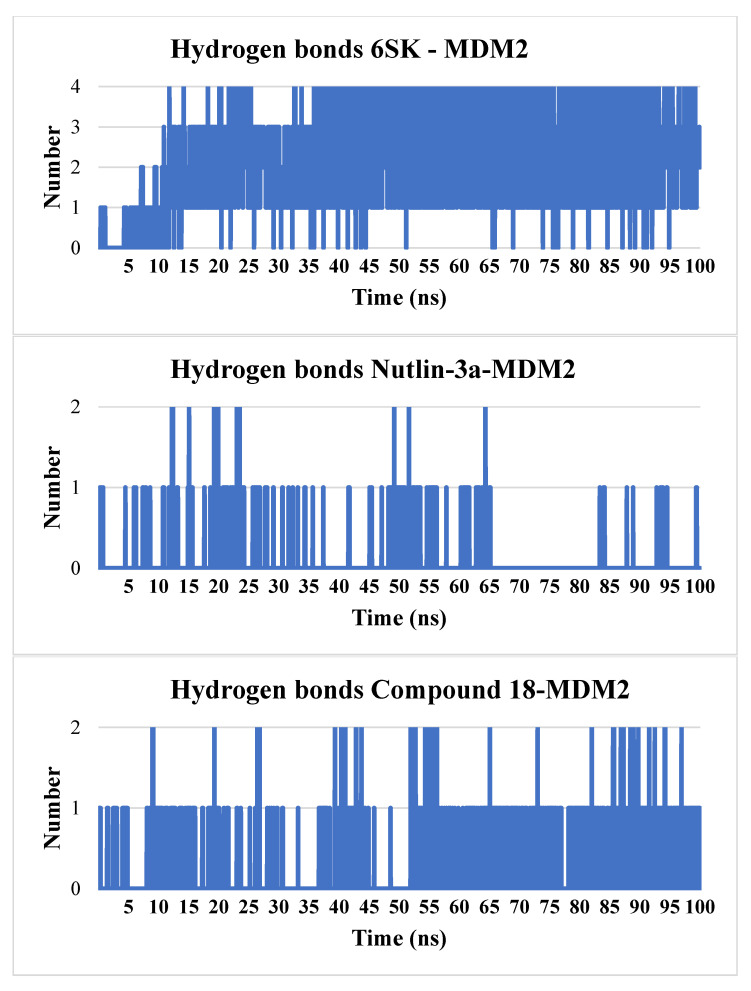
Time evolution of hydrogen bonds formed between MDM2 and ligands 6SK, Nutlin-3a, and Compound **18** during the 100 ns molecular dynamics simulation.

All-atom analysis of ligand–MDM2 hydrogen bonding over the 100 ns trajectories reveals markedly different interaction patterns for the three compounds. 6SK rapidly establishes hydrogen bonds with the protein, reaching up to four simultaneous contacts by ~15 ns; thereafter, it sustains between two and four hydrogen bonds for the majority of the simulation (mean ≈ 3), indicating a robust polar anchoring network. In contrast, Nutlin-3a forms at most two hydrogen bonds, but these are highly intermittent: brief spikes to two contacts occur around 15–25 ns and again near 50–60 ns, while for much of the trajectory it makes zero or a single hydrogen bond (mean < 0.5), underscoring weak and transient polar engagement. Compound **18** occupies an intermediate regime, typically maintaining one persistent hydrogen bond and occasionally forming a second (particularly between 50 and 70 ns), yielding an average of ≈1–1.5 contacts. Together with the RMSD and RMSF data, these hydrogen-bonding profiles reinforce that 6SK not only binds most stably in a conformational sense but also leverages the strongest and most enduring polar interactions with MDM2; Compound **18** achieves moderate hydrogen-bond stabilization, while Nutlin-3a relies predominantly on hydrophobic contacts and exhibits minimal, short-lived hydrogen bonding (Figure 8).

**Figure 9:**
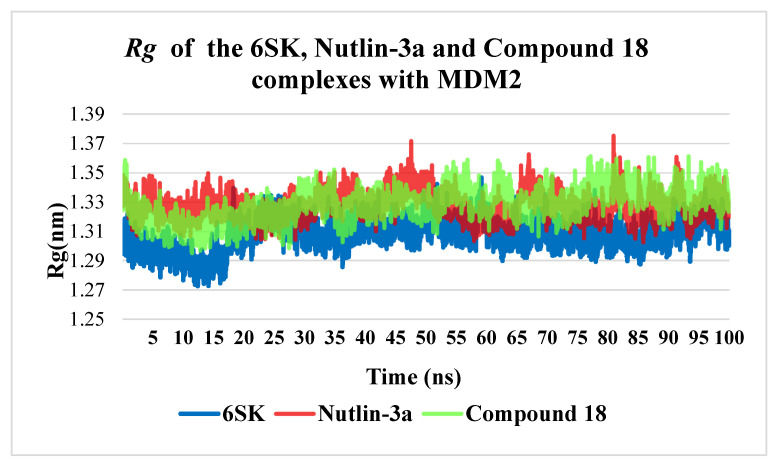
Radius of gyration (Rg) of MDM2 in complexes with ligands 6SK, Nutlin-3a, and Compound **18** during the 100 ns molecular dynamics simulation.

All-atom, 100 ns molecular-dynamics trajectories of MDM2 in complex with 6SK, Nutlin-3a, and Compound **18** were further interrogated by calculating the radius of gyration (Rg) of the protein backbone (residues 18–111) at 10 ps intervals. All three complexes maintained a stable global fold, with Rg values fluctuating within a narrow band throughout the simulation, yet clear ligand-dependent differences emerged. The MDM2–6SK complex exhibited the smallest average Rg of approximately 1.30 nm and the tightest distribution (±0.02 nm), indicative of a subtly more compact and rigid tertiary structure. By contrast, Nutlin-3a induced a higher mean Rg of about 1.33 nm and transient excursions up to 1.37 nm—most notably near 45 ns and 80 ns—reflecting episodic expansion of the overall protein envelope in line with the enhanced local flexibility and intermittent loss of hydrogen bonds observed earlier. Compound **18** produced an intermediate profile (mean ~1.33 nm, ±0.015 nm) but with fewer pronounced breathing motions than Nutlin-3a, suggesting it enforces moderate compaction without the structural tightness of 6SK. Together with the RMSD, RMSF and hydrogen-bonding data, these Rg trajectories confirm that 6SK not only stabilizes the binding pocket but also promotes the greatest overall cohesion of MDM2, whereas Nutlin-3a permits occasional global loosening and Compound **18** delivers balanced intermediate restraint. This multi-metric analysis underscores the importance of Rg as a complementary measure of ligand-induced allosteric effects on protein stability and supports the prioritization of Compound **18** for further lead optimization (Figure 9).

Analysis of interactions with key residues corroborates the trajectory data obtained for the three ligands. Notably, 6SK maintains persistent contacts with Tyr100 (75%), Leu54 (85%), and Leu57 (80%), reflecting its deeply buried and hydrophobically stabilized pose. Compound **18** also sustains strong interactions with Leu54 (65%) and Gly58 (58%), consistent with its designed halogen-bonding and hydrogen-bonding capabilities, and exhibits the highest level of engagement with His96 (75%). In contrast, Nutlin-3a shows considerably weaker and more transient interactions: its contacts with Leu54 and Leu57 persist only 43–45% of the time, while Gly58 (11%) and His96 (27%) interactions are essentially unstable. These contact occupancies parallel the hydrogen-bonding and RMSD profiles: the longer-lived interactions of 6SK and compound **18** correlate with their enhanced binding stability, whereas the fleeting contacts of Nutlin-3a mirror its higher mobility and presumably faster dissociation rate (Table 4).

Overall, 6SK demonstrates a pronounced reliance on hydrophobic interactions with lipophilic residues, yielding a more stable binding orientation. This observation is consistent with previous reports highlighting the central role of hydrophobic contacts in stabilizing ligands within the MDM2 pocket [38,39]. Compound **18**, supported by both hydrophobic anchoring and hydrogen bonding, displays a stronger dependence on hydrogen bonding, which further reinforces its binding pose. By contrast, the weaker and intermittent interactions of Nutlin-3a result in a more dynamic complex, underscoring how higher contact occupancies directly contribute to binding efficiency and reduced off-rates.

#### 2.4.3. ADMET Profile

Across the panel of 19 candidate molecules, in silico ADMET testing revealed a generally favorable drug-likeness profile. Thirteen structures—compounds **1**–**7**, **9**, **11**, **18**, and **19**—satisfied Lipinski’s Rule of Five without any violations, whereas six molecules (**8**, **10**, **12**–**14**, **17**) breached a single criterion, predominantly molecular weight (M) exceeding 500 Da, yet still remained within a developable chemical space. Only compounds **15** and **16** exceeded two Lipinski thresholds, combining elevated molecular mass (≈598 Da) with pronounced lipophilicity (MLogP > 5), factors that may compromise oral absorption and formulation.

Molecular weights ranged from 328 to 598 Da and MLogP values from 2.17 to 5.24. For the majority of compounds, lipophilicity was calculated as values between 2 and 4, a window conducive to balanced permeability and aqueous solubility; the two most hydrophobic entities (**15** and **16**) will likely necessitate solubilizing strategies or structural optimization.

Toxicological predictions placed every molecule in toxicity class IV (“harmful if swallowed”), with median lethal doses (LD_50_) spanning 693–1600 mg kg^−1^. Although this class is acceptable for early discovery, compounds exhibiting the lowest LD_50_ values (**1**, **11**, **13**, **15**) warrant heightened attention in subsequent sub-chronic safety assessments. The observed toxicity findings are consistent with the in vitro biological assay results, in which the tested compound exhibited a moderate toxicity profile (Table 5).

Of particular note, compound **18** fully complies with all Lipinski criteria (MW = 458.96 Da; MLogP = 4.50; HBA = 2; HBD = 1; zero violations) and maintains a moderate predicted LD_50_ of 1098 mg kg^−1^, positioning it among the most promising leads for further oral development. Collectively, the dataset indicates that most compounds occupy a drug-like physicochemical space and display only moderate acute toxicity. The lighter, more hydrophilic subset (**1**–**4**) and fully compliant compound **18** emerge as the strongest candidates for progression, whereas the heavier single-violation molecules (**8**, **10**, **12**–**14**, **17**) may advance provided permeability and metabolic stability are experimentally confirmed. In contrast, compounds **15** and **16** present a higher risk profile that demands either structural refinement or advanced formulation approaches before further preclinical investment.

## 3. Discussion

The newly synthesized spiro-isatin-thiazolidinone derivatives display clear advantages over earlier analogs such as Les-3467, which showed limited anticancer potency and poor selectivity, affecting both cancerous and pseudo-normal cells at higher concentrations. In contrast, several of the new derivatives, most notably compound **18**, exhibited low-micromolar cytotoxicity toward multiple cancer cell lines, including HCT116, MCF-7, MDA-MB-231, and KB3-1, while showing markedly reduced effects on NIH3T3 fibroblasts and HaCaT keratinocytes. This improvement in the selectivity index suggests that the design modifications successfully enhanced tumor-specific activity, potentially reducing off-target toxicity compared with both Les-3467 and standard agents such as doxorubicin.

In silico ADMET predictions support these findings. Compound **18** complies fully with Lipinski’s Rule of Five, with balanced molecular weight and lipophilicity, and shows a moderate predicted acute toxicity (oral LD_50_ ≈ 1098 mg kg^−1^). This favorable drug-likeness profile, combined with the reduced in vitro impact on non-malignant cells, indicates a potentially wider therapeutic window. Such properties mark a substantial advancement over earlier scaffolds, which were hindered by either suboptimal physicochemical parameters or significant normal-cell toxicity.

Docking studies reveal that the new series binds MDM2 with higher predicted affinity than Les-3467, and in some cases surpasses Nutlin-3a, a benchmark MDM2–p53 inhibitor. Compound **18** achieved a binding energy of −10.16 kcal/mol, comparable to the co-crystallized ligand 6SK and stronger than Nutlin-3a (−8.63 kcal/mol). Its binding pose engages key residues—forming hydrogen bonds with His96 and Gly58 and halogen bonds with Leu54 and Phe55—while its flexible benzyl and tert-butylphenylidene groups optimize hydrophobic pocket occupancy. Notably, the introduction of a more flexible substituent at the third position, by creating an additional torsional degree of freedom, allows improved adaptation to the hydrophobic cavity. This enhanced conformational adaptability facilitates more efficient pocket filling, thereby contributing to the overall strengthening of the anticancer effect. Furthermore, the presence of a halogen atom in the 5-position of the isatin ring was associated with improved anticancer activity, with fluorine substitution producing a more pronounced enhancement than chlorine. This difference may be attributed to the smaller atomic radius and higher electronegativity of fluorine, which can strengthen halogen bonding and improve overall ligand–receptor complementarity.

Of particular interest, derivatives containing pyridine fragments (compound **12** and **13**) demonstrated a pronounced and selective effect on certain cancer cell lines (KB3-1), with one compound showing the most potent response in the series. This effect was especially evident against the target line where its activity markedly exceeded that observed for other derivatives, despite exhibiting only moderate cytotoxicity toward the remaining tested cancer models. Such a pattern may suggest the presence of a specific mechanism of action that confers enhanced sensitivity in this cell type—potentially involving unique molecular targets or transport processes—while sparing other lines. However, this hypothesis requires further targeted studies to elucidate the underlying basis of this selectivity.

Molecular dynamics simulations further support compound **18’s** favorable interaction profile. Over 100 ns, the complex maintained low ligand RMSD values and dampened fluctuations in key MDM2 regions compared to Nutlin-3a. The interaction network of compound **18** stabilized both the immediate binding cleft and distal structural elements, a property potentially linked to longer target residence time. While another analog, 6SK, showed even tighter binding and more persistent hydrogen bonding, compound **18** offers a balanced profile of strong anchoring with moderate flexibility—attributes that may improve adaptability to target conformational changes in vivo.

Off-target activity toward PPARγ appears minimal for the new derivatives. Although compound **18** exhibited measurable docking affinity for PPARγ, it lacked interactions with key activation residues, reducing the likelihood of receptor agonism. This is a notable improvement over Les-3467, which displayed experimentally confirmed PPARγ modulation, potentially contributing to non-specific effects.

Overall, compound **18** combines broad-spectrum anticancer potency, improved selectivity for malignant cells, favorable drug-likeness, and stable, specific engagement of the MDM2 target. A comparative assessment of cytotoxic activity across the synthesized compound set reveals clear structure–activity trends that rationalize this performance. Derivatives lacking the 5-arylidene substituent (e.g., compounds 1–5) were largely inactive, indicating the crucial role of this moiety in target engagement. Among arylidene-bearing analogs, N-benzyl-substituted thiazolidinones demonstrated significantly higher potency compared to their N-aryl counterparts, suggesting that increased conformational flexibility may facilitate optimal binding. Substituents on the isatin core also modulated activity: halogens at position 5—especially Cl and F—enhanced cytotoxicity, while Br-substituted analogs were generally less effective. The nature of the para-substituent on the arylidene ring further influenced activity, with hydrophobic and electron-rich groups (e.g., 4-Cl, 4-OEt, 4-tBu) promoting potency, and polar functionalities (e.g., morpholine in compound 8) suppressing it. Of note, pyridine-containing derivatives showed selective activity in certain cancer lines, hinting at possible compound-specific mechanisms. Collectively, these findings validate key design principles such as hydrophobic pocket coverage, halogen bonding, and substituent flexibility, offering a clear path toward further scaffold optimization. The consistent SAR patterns and favorable bioactivity profiles support the continued exploration of spiro-isatin-thiazolidinones as possible agents for MDM2–p53 reactivation in cancer therapy.

## 4. Materials and Methods

### 4.1. Chemistry

#### 4.1.1. General Information

Melting points were measured in open capillary tubes on a BÜCHI B-545 melting point apparatus (BÜCHI Labortechnik AG, Flawil, Switzerland) and are uncorrected. The elemental analyses (C, H, N) were performed using the Perkin–Elmer 2400 CHN analyzer (PerkinElmer, Waltham, MA, USA) and were within ±0.4% of the theoretical values. The 600 MHz-1H and 151 MHz-13C spectra were recorded on a Bruker AVANCE-600 spectrometer (Bruker, Bremen, Germany). All spectra were recorded at room temperature and were referenced internally to solvent reference frequencies. Chemical shifts (δ) are quoted in ppm, and coupling constants (J) are reported in Hz. Solvents and reagents that are commercially available were used without further purification.

#### 4.1.2. Synthesis and Characterization of Spiro[3H-indol-3,2′-thiazolidin]-2,4′(1H)-diones (Compounds **1**–**5**)

A mixture of isatin or isatin’s derivatives (10 mmol) and the corresponding amine (10 mmol) in purified toluene (70 mL) containing a drop of acetic acid was heated at reflux for 6 h. Completion of the condensation to the respective imine was monitored chromatographically (TLC). Mercaptoacetic acid (30 mmol) was then added, and the reaction mixture was further refluxed in a Dean–Stark apparatus for 24 h. After cooling, the concentrated reaction mixture was poured into a saturated NaHCO_3_ solution. The resulting solid was collected by filtration and recrystallized from ethanol or acetic acid.

##### Characterization of Compounds **1**–**5**

5-Chloro-3′-(4-methoxyphenyl)spiro[indoline-3,2′-thiazolidine]-2,4′-dione (**1**)

White powder, yield 84%, mp 185–187 °C (ethanol). 1H NMR (600 MHz, DMSO-d6, δ): 10.88 (s, 1H, NH), 7.76 (s, 1H, arom.), 7.29 (d, J = 8.4 Hz, 1H, arom.), 7.01–6.96 (m, 2H, arom.), 6.90–6.84 (m, 2H, arom.), 6.77 (d, J = 8.3 Hz, 1H, arom.), 4.13 (d, J = 15.5 Hz, 1H, CH2), 4.02 (d, J = 15.5 Hz, 1H, CH2), 3.69 (s, 3H, CH3). 13C NMR (151 MHz, DMSO-d6, δ): 176.3 (C=O), 172.1 (C=O), 159.3, 140.8, 131.5, 130.1, 128.8, 127.9, 127.2, 127.0, 115.0, 112.6, 69.9 (C-2), 55.6 (CH3), 32.6 (C-5). Anal. calc. for C17H13ClN2O3S: C, 56.59%; H, 3.63%; N, 7.76%. Found: C, 56.80%; H, 3.90%; N, 7.90%.

5-Chloro-3′-(4-chlorophenyl)spiro[indoline-3,2′-thiazolidine]-2,4′-dione (**2**)

White powder, yield 78%, mp 88–90 °C (acetic acid). 1H NMR (600 MHz, DMSO-d6, δ): 10.98 (br.s, 1H, NH), 7.74 (s, 1H, arom.), 7.46–7.40 (m, 2H, arom.), 7.29 (d, J = 8.4 Hz, 1H, arom.), 7.12–7.07 (m, 2H, arom.), 6.81 (d, J = 8.3 Hz, 1H, arom.), 4.17 (d, J = 15.6 Hz, 1H, CH2), 4.06 (d, J = 15.6 Hz, 1H, CH2). 13C NMR (151 MHz, DMSO-d6, δ): 176.2 (C=O), 172.1 (C=O), 140.8, 135.4, 133.5, 131.7, 130.3, 130.0, 127.3, 126.9, 112.8, 69.7 (C-2), 32.7 (C-5). Anal. calc. for C16H10Cl2N2O2S: C, 52.62%; H, 2.76%; N, 7.67%. Found: C, 52.80%; H, 3.00%; N, 7.90%.

5-Bromo-3′-(4-bromophenyl)spiro[indoline-3,2′-thiazolidine]-2,4′-dione (**3**)

White powder, yield 86%, mp 178–180 °C (acetic acid). 1H NMR (600 MHz, DMSO-d6, δ): 10.98 (s, 1H, NH), 7.84 (s, 1H, arom.), 7.59–7.53 (m, 2H, arom.), 7.42 (d, J = 8.3, 1H, arom.), 7.03 (d, J = 8.3, 2H, arom.), 6.76 (d, J = 8.3 Hz, 1H, arom.), 4.16 (d, J = 15.6 Hz, 1H, CH2), 4.06 (d, J = 15.6 Hz, 1H, CH2). 13C NMR (151 MHz, DMSO-d6, δ): 176.1 (C=O), 172.1 (C=O), 141.2, 135.9, 134.6, 133.0, 130.7, 129.7, 127.7, 122.1, 114.9, 113.3, 69.6 (C-2), 32.8 (CH2). Anal. calc. for C16H10Br2N2O2S: C, 42.32%; H, 2.22%; N, 6.17%. Found: C, 42.50%; H, 2.40%; N, 6.40%.

3′-Benzyl-5-fluorospiro[indoline-3,2′-thiazolidine]-2,4′-dione (**4**)

Pink powder, yield 65%, mp 200–202 °C (ethanol). 1H NMR (600 MHz, DMSO-d6, δ): 10.70 (s, 1H, NH), 7.22–7.09 (m, 5H, arom.), 6.90 (d, J = 8.0 Hz, 2H, arom.), 6.83 (d, J = 8.6 Hz, 1H, arom.), 4.38 (d, J = 15.3 Hz, 1H, CH2), 4.07 (d, J = 15.2 Hz, 1H, CH2), 4.03 (d, J = 15.4 Hz, 1H, CH2), 3.97 (d, J = 15.4 Hz, 1H, CH2). 13C NMR (151 MHz, DMSO-d6, δ): 176.1 (C=O), 172.4 (C=O), 158.5 (d, JC-F = 158.5 Hz), 138.4, 136.0, 128.5, 128.4, 127.9, 126.2 (d, JC-F = 9.0 Hz), 118.3 (d, JC-F = 24.1 Hz), 114.3 (d, JC-F = 24.1 Hz), 112.1 (d, JC-F = 7.5 Hz), 69.2 (C-2), 46.6 (CH2), 32.6 (C-5). Anal. calc. for C17H13FN2O2S: C, 62.18%; H, 3.99%; N, 8.53%. Found: C, 62.30%; H, 4.20%; N, 8.70%.

3′-Benzyl-5-chlorospiro[indoline-3,2′-thiazolidine]-2,4′-dione (**5**)

White powder, yield 74%, mp 185–187 °C (ethanol). 1H NMR (600 MHz, DMSO-d6, δ): 10.48 (s, 1H, NH), 7.41 (s, 1H, arom.), 7.28 (d, J = 8.2 Hz, 1H, arom.), 7.16 (d, J = 8.2 Hz, 2H), 6.96 (br.s, 3H, arom.), 6.68 (d, J = 8.3 Hz, 1H, arom.), 4.22 (s, 2H, CH2), 4.05 (d, J = 15.8 Hz, 1H, CH2), 3.74 (d, J = 15.7 Hz, 1H, CH2). 13C NMR (151 MHz, DMSO-d6, δ): 177.1 (C=O), 176.0 (C=O), 142.7, 129.5, 128.7, 125.8, 125.7, 125.6, 124.0, 123.6, 111.4, 111.2, 56.31 (C-2), 46.1 (CH2), 39.5 (C-5). Anal. calc. for C17H13ClN2O2S: C, 59.22%; H, 3.80%; N, 8.12%. Found: C, 59.40%; H, 4.00%; N, 8.30%.

#### 4.1.3. Synthesis and Characterization of 5-Arylidene Derivatives of Spiro[3H-indol-3,2′-thiazolidin]-2,4′(1H)-diones (Compounds **6**–**19**)

A flask was charged with the appropriate compound **1**–**4** (5 mmol), the aromatic aldehyde (10% excess), potassium tert-butoxide (7.5 mmol), and isopropanol (15 mL). The mixture was heated under reflux for 3 h. After cooling, 1 mL of acetic acid was added. The precipitated product was collected by filtration and recrystallised from toluene or acetic acid.

##### Characterization of Compounds **6**–**19**

(Z)-5-Chloro-3′-(4-methoxyphenyl)-5′-((E)-3-phenylallylidene)spiro[indoline-3,2′-thiazolidine]-2,4′-dione (**6**)

Brown powder, yield 45%, mp 174–176 °C (toluene). 1H NMR (600 MHz, DMSO-d6, δ): 11.06 (s, 1H, NH), 7.90 (s, 1H, arom.), 7.61 (d, J = 7.5 Hz, 1H, arom.), 7.60 (d, J = 11.6 Hz, 1H, CH=), 7.43–7.28 (m, 5H, arom.), 7.12 (d, J = 15.4 Hz, 1H, CH=), 7.05 (d, J = 7.6 Hz, 2H, arom.), 6.98 (dd, J = 15.4, 11.6 Hz, 1H, CH=), 6.91 (d, J = 7.7 Hz, 2H, arom.), 6.82 (d, J = 8.4 Hz, 1H, arom.), 3.71 (s, 3H, CH3). 13C NMR (151 MHz, DMSO-d6, δ): 174.4 (C=O), 166.1 (C=O), 159.5, 141.0, 139.4, 136.6, 131.9, 130.4, 130.1, 129.3, 128.4, 127.6, 127.5, 127.3, 127.0, 126.6, 126.5, 124.2, 115.0, 112.9, 69.5 (C-2), 55.7 (CH3). Anal. calc. for C26H19ClN2O3S: C, 65.75%; H, 4.03%; N, 5.90%. Found: C, 65.90%; H, 4.20%; N, 6.10%.

(Z)-5-Chloro-5′-(4-chlorobenzylidene)-3′-(4-methoxyphenyl)spiro[indoline-3,2′-thiazolidine]-2,4′-dione (**7**)

White powder, yield 68%, mp 226–228 °C (acetic acid). 1H NMR (600 MHz, DMSO-d6, δ): 11.09 (br.s, 1H, NH), 7.94 (s, 1H, arom.), 7.64–7.57 (m, 3H, arom. + CH=), 7.55 (d, J = 7.8, 2H, arom.), 7.34 (d, J = 8.4, 1H, arom.), 7.07 (d, J = 7.4, 2H, arom.), 6.93 (d, J = 7.4, 2H, arom.), 6.82 (d, J = 8.4 Hz, 1H, arom.), 3.71 (s, 3H, CH3). 13C NMR (151 MHz, DMSO-d6, δ): 174.2 (C=O), 166.3 (C=O), 159.6, 141.4, 133.7, 133.6, 131.9, 131.4, 130.4, 129.5, 128.2, 127.4, 127.3, 126.7, 125.6, 125.3, 115.1, 112.9, 69.7 (C-2), 55.7 (CH3). Anal. calc. for C24H16Cl2N2O3S: C, 59.64%; H, 3.34%; N, 5.80%. Found: C, 59.90%; H, 3.50%; N, 6.00%.

(Z)-5-Chloro-3′-(4-methoxyphenyl)-5′-(4-morpholinobenzylidene)spiro[indoline-3,2′-thiazolidine]-2,4′-dione (**8**)

Yellow powder, yield 62%, mp 262–264 °C (toluene). 1H NMR (600 MHz, DMSO-d6, δ): 11.04 (s, 1H, NH), 7.90 (s, 1H, arom.), 7.52 (s, 1H, CH=), 7.46–7.41 (m, 2H, arom.), 7.33 (d, J = 8.4 Hz, 1H, arom.), 7.07–7.01 (m, 4H, arom.), 6.91 (d, J = 7.4, 2H, arom.), 6.82 (d, J = 8.4 Hz, 1H, arom.), 3.76–3.72 (m, 4H, 2*CH2), 3.71 (s, 3H, CH3), 3.22 (dd, J = 6.0, 3.8 Hz, 4H, 2*CH2). 13C NMR (151 MHz, DMSO-d6, δ): 174.5 (C=O), 166.9 (C=O), 159.5, 151.4, 141.1, 131.8, 131.3, 130.5, 130.1, 128.5, 127.4, 127.2, 127.1, 124.7, 119.6, 115.0, 114.9, 112.8, 69.5 (C-2), 66.3 (2*CH2), 55.7 (CH3), 47.7 (2*CH2). Anal. calc. for C28H24ClN3O4S: C, 62.98%; H, 4.53%; N, 7.87%. Found: C, 63.20%; H, 4.70%; N, 8.10%.

(Z)-5-Chloro-3′-(4-chlorophenyl)-5′-(4-ethoxybenzylidene)spiro[indoline-3,2′-thiazolidine]-2,4′-dione (**9**)

Yellow powder, yield 75%, mp 183–185 °C (acetic acid). 1H NMR (600 MHz, DMSO-d6, δ): 11.15 (s, 1H, NH), 7.92 (s, 1H, arom.), 7.61 (s, 1H, CH=), 7.49 (d, J = 7.7 Hz, 2H, arom.), 7.47 (d, J = 7.8 Hz, 2H, arom.), 7.35 (d, J = 8.4 Hz, 1H, arom.), 7.18 (d, J = 7.8 Hz, 2H, arom.), 7.03 (d, J = 7.7 Hz, 2H, arom.), 6.85 (d, J = 8.4 Hz, 1H, arom.), 4.08 (q, J = 7.0 Hz, 2H, CH2), 1.34 (t, J = 7.0 Hz, 3H, CH3). 13C NMR (151 MHz, DMSO-d6, δ): 174.3 (C=O), 166.7 (C=O), 159.5, 141.1, 135.1, 133.8, 131.7, 130.8, 130.0, 129.3, 128.6, 127.6, 126.9, 126.5, 125.7, 121.0, 115.4, 113.0, 69.4 (C-2), 63.8 (CH2), 15.0 (CH3). Anal. calc. for C25H18Cl2N2O3S: C, 60.37%; H, 3.65%; N, 5.63%. Found: C, 60.50%; H, 3.90%; N, 5.90%.

(Z)-5-Chloro-3′-(4-chlorophenyl)-5′-(4-morpholinobenzylidene)spiro[indoline-3,2′-thiazolidine]-2,4′-dione (**10**)

Brown powder, yield 52%, mp > 260 °C (toluene). 1H NMR (600 MHz, DMSO-d6, δ): 11.12 (s, 1H), 7.91 (s, 1H, arom.), 7.55 (s, 1H, CH=), 7.48 (d, J = 7.7 Hz, 2H, arom.), 7.46 (d, J = 7.8 Hz, 2H, arom.), 7.35 (d, J = 8.4 Hz, 1H, arom.), 7.17 (d, J = 7.8 Hz, 2H, arom.), 7.04 (d, J = 7.8 Hz, 2H, arom.), 6.84 (d, J = 8.4 Hz, 1H, arom.), 3.76–3.71 (m, 4H, 2*CH2), 3.25–3.21 (m, 4H, 2* CH2). 13C NMR (151 MHz, DMSO-d6, δ): 174.4 (C=O), 166.1 (C=O), 159.5, 141.0, 139.4, 136.6, 131.9, 130.4, 129.3, 128.4, 127.6, 127.5, 127.3, 127.0, 126.6, 124.2, 115.0, 112.8, 69.5 (C-2), 55.73 (2*CH2), 40.5 (2*CH2). Anal. calc. for C27H21Cl2N3O3S: C, 60.23%; H, 3.93%; N, 7.80%. Found: C, 60.50%; H, 4.10%; N, 8.00%.

(Z)-5-Chloro-5′-(4-chlorobenzylidene)-3′-(4-chlorophenyl)spiro[indoline-3,2′-thiazolidine]-2,4′-dione (**11**)

Yellow powder, yield 74%, mp 194–196 °C (acetic acid). 1H NMR (600 MHz, DMSO-d6, δ): 11.20 (s, 1H, NH), 7.95 (s, 1H, arom.), 7.66 (s, 1H, CH=), 7.61 (d, J = 7.8 Hz, 2H, arom.), 7.57 (d, J = 7.8 Hz, 2H, arom.), 7.50 (d, J = 7.7 Hz, 2H, arom.), 7.35 (d, J = 8.4Hz, 1H), 7.19 (d, J = 7.8 Hz, 2H, arom.), 6.84 (d, J = 8.4 Hz, 1H). 13C NMR (151 MHz, DMSO-d6, δ): 174.0 (C=O), 166.3 (C=O), 141.2, 134.9, 134.0, 133.9, 133.5, 132.2, 131.5, 130.7, 130.1, 129.6, 127.6, 127.3, 126.2, 125.8, 125.2, 113.1, 69.5 (C-2). Anal. calc. for C23H13Cl3N2O2S: C, 56.63%; H, 2.69%; N, 5.74%. Found: C, 56.80%; H, 2.90%; N, 5.90%.

(Z)-5-Chloro-3′-(4-methoxyphenyl)-5′-(4-(pyridin-2-yl)benzylidene)spiro[indoline-3,2′-thiazolidine]-2,4′-dione (**12**)

Yellow powder, yield 66%, mp 199–201 °C (toluene). 1H NMR (600 MHz, DMSO-d6, δ): 11.11 (s, 1H, NH), 8.70 (d, J = 4.8 Hz, 1H, arom.), 8.23 (d, J = 8.4 Hz, 2H, arom.), 8.05 (d, J = 8.2 Hz, 1H, arom.), 7.97 (s, 1H, arom.), 7.91 (td, J = 7.7, 1.8 Hz, 1H), 7.73–7.68 (m, 3H, arom. + CH=), 7.35 (d, J = 8.4 Hz, 1H, arom.), 7.08 (d, J = 7.6 Hz, 2H, arom.), 6.94 (d, J = 7.6 Hz, 2H, arom.), 6.83 (d, J = 8.4 Hz, 1H, arom.), 3.72 (s, 3H, CH3). 13C NMR (151 MHz, DMSO-d6, δ): 174.2 (C=O), 166.5 (C=O), 159.7, 155.5, 150.2, 141.2, 139.1, 137.8, 135.3, 132.0, 130.5, 130.3, 129.4, 128.7, 128.3, 127.5, 126.8, 126.2, 125.8, 125.3, 121.0, 115.1, 113.0, 69.8 (C-2), 55.8 (CH3). Anal. calc. for C29H20ClN3O3S: C, 66.22%; H, 3.83%; N, 7.99%. Found: C, 66.50%; H, 4.00%; N, 8.20%.

(Z)-5-Chloro-3′-(4-chlorophenyl)-5′-(4-(pyridin-2-yl)benzylidene)spiro[indoline-3,2′-thiazolidine]-2,4′-dione (**13**)

Brown powder, yield 70%, mp 195–197 °C (toluene). 1H NMR (600 MHz, DMSO-d6, δ): 11.11 (s, 1H, NH), 8.70 (d, J = 4.8 Hz, 1H, arom.), 8.24 (d, J = 8.4 Hz, 2H, arom.), 8.06 (d, J = 8.0 Hz, 1H, arom.), 7.98 (s, 1H, arom.), 7.92 (td, J = 7.7, 1.8 Hz, 1H), 7.73–7.68 (m, 3H, arom. + CH=), 7.51 (d, J = 8.6 Hz, 2H, arom.), 7.37 (d, J = 8.5 Hz, 1H, arom.), 7.26 (t, J = 7.6 Hz, 1H, arom.), 7.20 (d, J = 8.6 Hz, 2H, arom.), 6.86 (d, J = 8.4 Hz, 1H, arom.). 13C NMR (151 MHz, DMSO-d6, δ): 174.1 (C=O), 166.5 (C=O), 155.4, 150.2, 141.2, 139.3, 137.9, 135.2, 134.0, 132.2, 130.8, 130.4, 130.2, 129.4, 128.7, 127.5, 126.6, 126.3, 125.8, 125.0, 123.5, 121.0, 113.1, 69.6 (C-2). Anal. calc. for C28H17Cl2N3O2S: C, 63.40%; H, 3.23%; N, 7.92%. Found: C, 63.60%; H, 3.50%; N, 8.10%.

(Z)-5-Bromo-3′-(4-bromophenyl)-5′-(4-ethoxybenzylidene)spiro[indoline-3,2′-thiazolidine]-2,4′-dione (**14**)

Yellow powder, yield 77%, mp 190–192 °C (acetic acid). 1H NMR (600 MHz, DMSO-d6, δ): 11.15 (s, 1H, NH), 8.02 (s, 1H, arom.), 7.63–7.58 (m, 3H, arom. + CH=), 7.51 (d, J = 8.0 Hz, 2H, arom.), 7.47 (d, J = 8.5 Hz, 1H, arom.), 7.10 (d, J = 8.0 Hz, 2H, arom.), 7.04 (d, J = 8.0 Hz, 2H, arom.), 6.80 (d, J = 8.3 Hz, 1H, arom.), 4.08 (q, J = 6.9 Hz, 2H, CH2), 1.34 (t, J = 6.9 Hz, 3H, CH3). 13C NMR (151 MHz, DMSO-d6, δ): 174.1 (C=O), 166.6 (C=O), 159.5, 141.5, 135.6, 134.9, 133.0, 131.7, 131.0, 129.9, 127.2, 126.9, 126.7, 122.4, 121.0, 115.4, 115.2, 113.4, 69.2 (C-2), 63.8 (CH2), 15.0 (CH3). Anal. calc. for C25H18Br2N2O3S: C, 51.22%; H, 3.09%; N, 4.78%. Found: C, 51.50%; H, 3.30%; N, 5.00%.

(Z)-5′-(4-(Tert-butyl)benzylidene)-5-chloro-3′-(4-chlorophenyl)spiro[indoline-3,2′-thiazolidine]-2,4′-dione (**15**)

Yellow powder, yield 80%, mp > 260 °C (acetic acid). 1H NMR (600 MHz, DMSO-d6, δ): 11.15 (s, 1H, NH), 7.94 (s, 1H, arom.), 7.63 (s, 1H, CH=), 7.54–7.46 (m, 6H), 7.35 (d, J = 8.4 Hz, 1H, arom.), 7.19 (d, J = 7.9 Hz, 2H, arom.), 6.85 (d, J = 8.4 Hz, 1H, arom.), 1.30 (s, 9H, 3*CH3). 13C NMR (151 MHz, DMSO-d6, δ): 174.1 (C=O), 166.5 (C=O), 152.4, 141.1, 135.0, 133.9, 132.1, 131.8, 130.8, 130.1, 129.7, 127.6, 127.3, 127.1, 126.4, 126.3, 123.3, 113.0, 69.4 (C-2), 35.1 (C-(CH3)3), 31.3 (3*CH3). Anal. calc. for C27H22Cl2N2O2S: C, 63.66%; H, 4.35%; N, 5.50%. Found: C, 63.90%; H, 4.60%; N, 5.70%.

(Z)-5-Bromo-3′-(4-bromophenyl)-5′-(4-(tert-butyl)benzylidene)spiro[indoline-3,2′-thiazolidine]-2,4′-dione (**16**)

Yellow powder, yield 74%, mp > 260 °C (acetic acid). 1H NMR (600 MHz, DMSO-d6, δ): 11.16 (s, 1H, NH), 8.04 (s, 1H, arom.), 7.65–7.59 (m, 3H, arom. + CH=), 7.55–7.46 (m, 5H, arom.), 7.11 (d, J = 8.0 Hz, 2H, arom.), 6.80 (d, J = 8.4 Hz, 1H, arom.), 1.30 (s, 9H, 3*CH3). 13C NMR (151 MHz, DMSO-d6, δ): 174.1 (C=O), 166.6 (C=O), 152.4, 141.6, 135.5, 135.0, 133.1, 131.9, 131.1, 130.0, 129.8, 127.1, 126.8, 126.4, 123.4, 122.5, 115.2, 113.5, 69.3 (C-2), 35.1 (C-(CH3)3), 31.4 (3*CH3). Anal. calc. for C27H22Br2N2O2S: C, 54.20%; H, 3.71%; N, 4.68%. Found: C, 54.40%; H, 3.90%; N, 4.90%.

(Z)-3′-Benzyl-5′-(4-(tert-butyl)benzylidene)-5-chlorospiro[indoline-3,2′-thiazolidine]-2,4′-dione (**17**)

Yellow powder, yield 82%, mp 163–165 °C (toluene). 1H NMR (600 MHz, DMSO-d6, δ): 11.03 (s, 1H, NH), 7.56 (s, 1H, CH=), 7.49–7.39 (m, 4H, arom.), 7.30 (d, J = 8.4, Hz, 1H, arom.), 7.19–7.09 (m, 3H, arom.), 7.04 (d, J = 8.2 Hz, 1H, arom.), 6.96–6.91 (m, 2H, arom.), 6.86 (d, J = 8.4 Hz, 1H, arom.), 4.60 (d, J = 15.3 Hz, 1H, CH2), 4.09 (d, J = 15.3 Hz, 1H, CH2), 1.24 (s, 9H, 3*CH3). 13C NMR (151 MHz, DMSO-d6, δ): 173.8 (C=O), 167.0 (C=O), 152.2, 141.4, 136.0, 132.0, 131.9, 129.7, 128.6, 128.5, 128.0, 127.2, 127.1, 126.6, 126.3, 125.8, 123.6, 112.9, 69.1 (C-2), 47.0 (CH2), 35.1 (C-(CH3)3), 31.4 3*CH3. Anal. calc. for C28H25ClN2O2S: C, 68.77%; H, 5.15%; N, 5.73%. Found: C, 69.00%; H, 5.40%; N, 5.90%.

(Z)-3′-Benzyl-5′-(4-(tert-butyl)benzylidene)-5-fluorospiro[indoline-3,2′-thiazolidine]-2,4′-dione (**18**)

White powder, yield 68%, mp 157–159 °C (toluene). 1H NMR (600 MHz, DMSO-d6, δ): 10.97 (s, 1H, NH), 7.61 (s, 1H, CH=), 7.53–7.48 (m, 2H, arom.), 7.46 (d, J = 8.6 Hz, 2H, arom.), 7.23–7.16 (m, 2H, arom.), 7.19–7.13 (m, 2H, arom.), 7.02–6.97 (m, 3H, arom.), 6.90 (d, J = 8.6 Hz, 1H, arom.), 4.61 (d, J = 15.4 Hz, 1H, CH2), 4.17 (d, J = 15.4 Hz, 1H, CH2), 1.29 (s, 9H, 3*CH3). 13C NMR (151 MHz, DMSO-d6, δ): 174.0 (C=O), 167.0 (C=O), 158.6 (d, JC-F = 238.5 Hz), 152.1, 138.8, 136.0, 132.0, 129.6, 128.6, 128.5, 127.9, 126.3 (d, JC-F = 9.0 Hz), 125.4 (d, JC-F = 9.0 Hz), 123.7, 118.6 (d, JC-F = 24.6 Hz), 114.5 (d, JC-F = 24.1 Hz), 112.5 (d, JC-F = 7.5 Hz), 69.3 (C-2), 47.0 (CH2), 35.0 (C-(CH3)3), 31.3 (3*CH3). Anal. calc. for C28H25FN2O2S: C, 71.16%; H, 5.33%; N, 5.93%. Found: C, 71.30%; H, 5.50%; N, 6.10%.

(Z)-3′-Benzyl-5′-(4-ethoxybenzylidene)-5-fluorospiro[indoline-3,2′-thiazolidine]-2,4′-dione (**19**)

White powder, yield 74%, mp 163–165 °C (acetic acid). 1H NMR (600 MHz, DMSO-d6, δ): 10.97 (s, 1H, NH), 7.59 (s, 1H, CH=), 7.50–7.45 (m, 2H, arom.), 7.22–7.10 (m, 4H, arom.), 7.05–7.00 (m, 2H, arom.), 6.99 (d, J = 8.1 Hz, 3H, arom.), 6.90 (d, J = 8.6 Hz, 1H, arom.), 4.62 (d, J = 15.4 Hz, 1H, CH2), 4.15 (d, J = 15.4 Hz, 1H, CH2), 4.07 (q, J = 6.9 Hz, 2H, CH2), 1.33 (t, J = 6.9 Hz, 3H, CH3). 13C NMR (151 MHz, DMSO-d6, δ): 174.2 (C=O), 167.2 (C=O), 158.6 (d, JC-F = 239.7 Hz), 138.7, 136.1, 131.6, 128.6, 128.55, 128.51, 127.9, 127.17, 126.59, 125.5 (d, JC-F = 10.5 Hz), 121.52, 118.8 (d, JC-F = 28.6 Hz), 115.49, 114.6 (d, JC-F = 31.7 Hz), 112.5 (d, JC-F = 10.5 Hz), 69.2 (C-2), 63.8 (CH2), 47.04 (CH2), 15.0 (CH3). Anal. calc. for C26H21FN2O3S: C, 67.81%; H, 4.60%; N, 6.08%. Found: C, 68.00%; H, 4.80%; N, 6.30%.

### 4.2. Cell Lines and Culture Conditions

Human breast adenocarcinoma cells of MCF-7 and MDA-MB-231 lines, human glioblastoma cells of U373 line, human chronic myelogenous leukemia cells of K562 line, human colorectal carcinoma cells of HCT116 line, and human epidermoid carcinoma cells of KB3-1 line were kindly donated by a Collection of the Institute for Cancer Research at Vienna Medical University (Vienna, Austria). Human keratinocytes of HaCaT line, murine breast carcinoma cells of 4T1 line, and murine fibroblasts of the NIH3T3 line were obtained from the Cell Collection of the R.E. Kavetsky Institute of Experimental Pathology, Oncology and Radiobiology of the National Academy of Sciences of Ukraine (Kyiv, Ukraine). MCF-7, MDA-MB-231, U373, K562, HaCaT, and NIH3T3 cells were cultured in DMEM medium (Sigma-Aldrich, Burlington, MA, USA) supplemented with 2 mM L-glutamine, 4500 mg/L glucose, and 1 mM sodium pyruvate. HCT116 and KB3-1 cells were maintained in RPMI-1640 medium supplemented with 2 mM L-glutamine (Sigma-Aldrich, USA). Both media were supplemented with 10% fetal bovine serum (Sigma-Aldrich, USA). All cell lines were incubated at 37 °C in a humidified atmosphere containing 5% CO_2_.

### 4.3. The MTT Assay

The substrate cells were seeded at a density of 5000 cells per 100 µL, while suspension K562 cells were seeded at 15,000 cells per 100 µL in 96-well plates and incubated overnight. Doxorubicin (Dox, Sigma-Aldrich, USA) was used as a reference drug. Studied substances were then added in 100 µL of culture medium, and the cells were further incubated for 72 h. Subsequently, 20 µL of MTT solution (5 mg/mL, Sigma-Aldrich, USA) was added to each well and incubated for 2–4 h. Formazan crystals were then solubilized by adding dimethyl sulfoxide (Sigma-Aldrich, USA). Absorbance was measured using a BioTek ELx800 Absorbance Reader (BioTek Instruments, Inc., Winooski, VT, USA). The results were analyzed and illustrated using GraphPad Prism (version 8.0.1; GraphPad Software, San Diego, CA, USA) and presented as a mean (M) ± standard deviation (SD). The IC50 values of the compounds were determined using nonlinear regression analysis in GraphPad Prism 10.4.2 [40].

### 4.4. Molecular Docking Protocol

Receptor and ligand structures were prepared according to the following workflow. The crystal structure of human MDM2 (PDB ID: 5LAV) [41] was retrieved from the Protein Data Bank. All crystallographic water molecules and co-crystallized ligands were removed, and missing side chains and hydrogens were added using AutoDockTools 1.5.6. The final receptor structure was converted to PDBQT format, with Kollman charges assigned and all non-polar hydrogens merged. The structures of the reference ligands 6SK and nutlin-3a were obtained from the PDB. The structures of the synthesized compounds were drawn in ChemOffice 22 and optimized in Chem3D using the MMFF94 force field [42] (20,000 iterations, RMS 0.01). Protonation states were checked at physiological pH; Gasteiger charges were then assigned, and rotatable bonds were defined automatically in AutoDockTools. Each ligand was saved in PDBQT format. Molecular docking was performed with AutoDock Vina 1.2.5 [43]. A cubic search box (20.625 Å × 20.625 Å × 20.625 Å) was centered on the MDM2–p53-binding cleft (coordinates x = −6.833, y = 11.116, z = −3.007), encompassing all known hot-spot residues (Phe19, Trp23, Leu26). Exhaustiveness was set to 32, and up to 10 binding modes were generated per ligand with an energy range of 4 kcal mol^−1^. All other settings were left at Vina defaults. During the validation redocking procedure, the obtained RMSD value of 0.954 indicated that the selected model was capable of predicting binding energies and ligand positions within MDM2 with satisfactory accuracy [44]. Docking poses were ranked by predicted binding affinity (kcal mol^−1^). The top poses for each ligand were visually inspected in BIOVIA Discovery Visualizer to verify the preservation of key hydrophobic and hydrogen-bond interactions. The best poses of 6SK, nutlin-3a, and compound **18** in complex with MDM2 were then subjected to 100 ns molecular dynamics simulations to validate binding stability.

### 4.5. Molecular Dynamics

The molecular-dynamics trajectories were generated with the cloud-based SiBioLead pipeline, which wraps a fully automated GROMACS 2023 workflow behind a web interface [45]. After uploading the top-ranked docking pose of compound **18** and two reference ligand–MDM2 complexes, the platform automatically generated ligand topologies compatible with OPLS/AA and applied the OPLS/AA force field to the protein. A rhombic-dodecahedral unit cell (triclinic representation) was constructed and solvated with SPC water. NaCl (0.15 M) was added to mimic physiological ionic strength and neutralize the systems. Energy minimization was performed using the steepest-descent algorithm, followed by 1000 ps of equilibration. Production MD simulations of 100 ns were carried out in GROMACS, and trajectories were analyzed for RMSD, RMSF, radius of gyration (Rg), and hydrogen-bond metrics to assess complex stability and residue–ligand contact frequencies (cutoff distance 3.5 Å). Trajectories were analyzed using GROMACS and Visual Molecular Dynamics (VMD 1.9.4) [46]. The percentage of simulation time each key amino acid remained in contact with the ligand was calculated from the trajectory using built-in VMD scripts.

### 4.6. ADMET Profiles

In silico prediction of ADMET parameters was performed using the freely available web platforms SwissADME [47] and ProTox-III [48]. The structures of the synthesized compounds were converted to SMILES notation and submitted to the respective platforms. Physicochemical descriptors relevant to Lipinski’s Rule of Five (Ro5)—molecular weight (MW), hydrogen-bond donors (HBDs), hydrogen-bond acceptors (HBAs), and lipophilicity—were computed in SwissADME, with Moriguchi’s MLOGP used as the primary logP estimator. Compounds were classified as Ro5-compliant (zero violations), single-violation, or multiple-violation according to standard thresholds (MW ≤ 500 Da, MLOGP ≤ 5, HBD ≤ 5, HBA ≤ 10). Predicted acute oral toxicity was obtained from ProTox-III, which reports both toxicity class and median lethal dose (LD_50_, mg·kg^−1^).

## Figures and Tables

**Table 1:** AutoDock Vina1.2.5 docking score for based compounds and reference ligands.

Compound	MDM2 (PDB 5LAV)	PPARγ (PDB 5YCP)
Les-3390	−9.342	−7.765
Les-3467	−8.801	−8.189
6SK	−10.151	-
BRL (Rosiglitazone)	-	−8.939

**Table 2:** The half-maximal inhibitory concentration (IC_50_) of spiro-thiazolidinone-isatin conjugates in the studied mammalian cells.

Comp.	HCT116	MCF-7	MDA-MB-231	4T1	KB3-1	K562	U373	HaCaT (Pseudo-Normal)	NIH3T3 (Pseudo-Normal)
**1**	>100	>100	85.31 ± 1.15	89.39 ± 1.93	62.3 ± 0.51	71.84 ± 7.79	76.70 ± 0.54	96.73 ± 1.02	77.56 ± 1.82
**2**	>100	84.93 ± 6.51	62.69 ± 0.22	73.03 ± 7.02	51.77 ± 0.26	9.96 ± 1.17	77.91 ± 0.56	82.78 ± 1.94	98.25 ± 0.64
**3**	88.93 ± 6.33	64.02 ± 4.77	56.49 ± 1.93	43.81 ± 1.76	46.71 ± 0.54	7.99 ± 0.12	49.44 ± 1.33	38.43 ± 0.77	82.93 ± 2.05
**4**	>100	>100	99.21 ± 0.41	91.81 ± 1.55	60.21 ± 0.72	88.75 ± 4.06	98.60 ± 1.92	>100	>100
**5**	92.79 ± 1.09	42.60 ± 1.24	68.79 ± 0.62	95.27 ± 1.07	52.09 ± 0.67	95.51 ± 2.64	76.21 ± 0.80	>100	>100
**6**	>100	83.28 ± 6.03	55.85 ± 5.67	67.98 ± 2.60	45.23 ± 0.36	>100	69.51 ± 0.90	85.34 ± 1.27	>100
**7**	>100	52.18 ± 2.15	8.55 ± 0.06	28.96 ± 0.92	17.16 ± 0.21	27.66 ± 3.02	72.34 ± 0.80	8.63 ± 0.57	79.74 ± 0.93
**8**	>100	>100	>100	>100	75.93 ± 4.21	>100	>100	>100	>100
**9**	>100	74.46 ± 4.73	7.89 ± 0.33	7.84 ± 0.49	22.3 ± 1.17	67.11 ± 0.70	98.22 ± 0.87	>100	>100
**10**	>100	>100	>100	>100	16.92 ± 0.86	>100	>100	>100	>100
**11**	>100	6.00 ± 0.28	6.37 ± 0.11	5.66 ± 0.27	6.95 ± 0.14	74.35 ± 0.66	66.91 ± 0.83	8.67 ± 0.49	>100
**12**	>100	>100	>100	>100	0.97 ± 0.11	>100	>100	>100	>100
**13**	41.38 ± 0.62	>100	>100	>100	0.99 ± 0.08	5.55 ± 0.17	>100	>100	>100
**14**	>100	>100	60.24 ± 0.62	>100	19.55 ± 0.23	>100	89.52 ± 0.81	>100	>100
**15**	>100	77.42 ± 2.90	30.72 ± 0.45	46.60 ± 2.98	9.77 ± 0.78	>100	79.33 ± 1.31	>100	90.32 ± 0.68
**16**	>100	>100	53.27 ± 0.51	69.05 ± 5.11	29.47 ± 0.33	>100	>100	>100	>100
**17**	8.24 ± 0.26	6.64 ± 0.39	13.52 ± 2.21	65.98 ± 4.48	5.85 ± 0.42	67.86 ± 0.81	32.26 ± 0.25	10.56 ± 0.21	91.00 ± 1.34
**18**	8.37 ± 0.51	6.99 ± 0.16	6.67 ± 0.15	46.74 ± 1.19	7.92 ± 0.65	37.37 ± 0.49	29.81 ± 0.35	>100	98.39 ± 0.66
**19**	62.68 ± 0.76	63.90 ± 0.59	59.31 ± 2.27	60.51 ± 1.84	34.98 ± 0.48	>100	54.89 ± 0.33	96.79 ± 0.78	94.35 ± 0.87
Dox	0.90 ± 0.11	0.62 ± 0.12	0.60 ± 0.08	0.73 ± 0.10	0.53 ± 0.08	0.95 ± 0.11	0.35 ± 0.09	3.1 ± 0.18	0.63 ± 0.12

**Table 3:** AutoDock Vina binding energies with the MDM2 (PDB 5LAV) and PPARγ (PDB 5ICP).

Compound	MDM2 (PDB 5LAV)	PPARγ (PDB 5YCP)
**1**	−7.263	−6.273
**2**	−7.781	−6.538
**3**	−7.202	−7.399
**4**	−8.934	−7.212
**5**	−8.522	−6.234
**6**	−8.480	−5.868
**7**	−9.528	−5.861
**8**	−8.621	−4.434
**9**	−8.243	−5.952
**10**	−8.408	−4.789
**11**	−8.694	−5.755
**12**	−8.638	−4.520
**13**	−8.427	−4.449
**14**	−7.280	−4.745
**15**	−8.141	−4.411
**16**	−7.964	−1.728
**17**	−9.720	−7.861
**18**	−10.160	−8.513
**19**	−9.423	−7.978
NUT (Nutlin-3a)	−8.632	-
8LX (Lobeglitazone)	-	−9.755

**Table 4:** Percentage of simulation frames during which key MDM2 residues maintain stable contacts (hydrophobic, H-bonds, halogen bonds) with each ligand over 100 ns of MD.

Residue (Interaction)	Compound 18 Time of Interaction (%)	6SK Time of Interaction (%)	Nutlin-3a Time of Interaction (%)
His96 (H-bond, hyd)	75	55	27
Leu54 (H-bond, halogen/hydrophobic)	65	85	45
Phe55 (halogen/hydrophobic)	63	30	10
Leu57 (hydrophobic)	43	80	43
Gly58 (H-bond hydrophobic)	58	30	11
Tyr100 (hydrophobic, halogen)	44	75	15

**Table 5:** Evaluation of drug-likeness based on Lipinski’s Rule of Five and predicted toxicity profiles of the compounds, including the number of hydrogen bond acceptors (*HBA*s), hydrogen bond donors (*HBD*s), molecular weight (M), lipophilicity calculated by the Moriguchi method (*MLogP*), number of rule violations, toxicity class (*TClass*), and median lethal dose (*LD_50_*).

Compound	Lipinski’s Rules of Five					Toxicity Profile	
	HBA	HBD	M	MLogP	Violation	TClass	LD50
**1**	3	1	360.81	2.17	0	IV	693
**2**	2	1	365.23	3.00	0	IV	1098
**3**	2	1	454.14	3.24	0	IV	1313
**4**	3	1	328.36	2.62	0	IV	1313
**5**	2	1	344.82	2.73	0	IV	1098
**6**	3	1	474.96	3.75	0	IV	1600
**7**	3	1	483.37	3.89	0	IV	1098
**8**	4	1	534.03	2.87	1	IV	1600
**9**	3	1	497.39	4.09	0	IV	1600
**10**	3	1	538.44	3.67	1	IV	1600
**11**	2	1	487.79	4.72	0	IV	693
**12**	4	1	526.01	3.39	1	IV	1600
**13**	3	1	530.42	4.29	1	IV	693
**14**	3	1	586.30	4.29	1	IV	1000
**15**	2	1	509.45	5.04	2	IV	693
**16**	2	1	598.35	5.24	2	IV	1600
**17**	2	1	489.02	4.78	1	IV	1098
**18**	3	1	472.57	4.68	0	IV	1024
**19**	4	1	460.52	3.72	0	IV	1313

## Data Availability

Data are contained within the article. Simulation input and output files, processed trajectories, and any additional datasets that support the findings of this work are available from the corresponding author upon reasonable request.

## Supplementary Materials

The following supporting information can be downloaded at: https://www.mdpi.com/article/10.3390/ph18101502/s1. Figures S1–S38: Copies of 1H and 13C NMR spectra of compounds **1**–**19**. Figures S39: The cytotoxic effects of the investigated spiro-thiazolidinone–isatin conjugates **1**–**19** and doxorubicin.

## Abbreviations

ADMET	Absorption, Distribution, Metabolism, Excretion, and Toxicity
HBA	Hydrogen Bond Acceptor
HBD	Hydrogen Bond Donor
IC50	Half-Maximal Inhibitory Concentration
LD50	Median Lethal Dose
MD	Molecular Dynamics
MDM2	Mouse Double Minute 2 homolog
NMR	Nuclear Magnetic Resonance
PPARγ	Peroxisome Proliferator-Activated Receptor Gamma
RMSD	Root-Mean-Square Deviation
RMSF	Root-Mean-Square Fluctuation
