# Supplementary material for: Design and Optimization of Spiro-Isatin-Thiazolidinone Hybrids with Promising Anticancer Activity
Source: Pharmaceuticals (Basel). 2025 Oct 7;18(10):1502. doi: 10.3390/ph18101502 (PMC12567349; doi:10.3390/ph18101502)
Supplement: Supplementary file 1 [file pharmaceuticals-18-01502-s001.zip › pharmaceuticals-3889665-supplementary.pdf]

Supplementary Materials

Design and Optimization of Spiro-Isatin-Thiazolidinone Hybrids with Promising Anticancer Activity

Dmytro Khylyuk [1,*], Serhii Holota [2,3], Natalia Finiuk [4], Rostyslav Stoika [4], Tetyana Rumynska [5] and Roman Lesyk [2,6,*]

[1] Chair and Department of Organic Chemistry, Medical University of Lublin, ul. Chodźki 4a, 20-093 Lublin, Poland
[2] Department of Pharmaceutical, Organic and Bioorganic Chemistry, Danylo Halytsky Lviv National Medical University, 69 Pekarska St., 79010 Lviv, Ukraine; golota_serg@yahoo.com
[3] Department of Organic and Pharmaceutical Chemistry, Lesya Ukrainka Volyn National University, 13 Volya Ave., 43025 Lutsk, Ukraine
[4] Institute of Cell Biology of National Academy of Sciences of Ukraine, 14/16 Drahomanov Str., 79005 Lviv, Ukraine; nataliyafiniuk@gmail.com (N.F.); stoika.rostyslav@gmail.com (R.S.)
[5] Department of Microbiology, Danylo Halytsky Lviv National Medical University, 69 Pekarska St., 79010 Lviv, Ukraine; tanityshka.r@gmail.com
[6] Department of Biotechnology and Cell Biology, Medical College, University of Information Technology and Management in Rzeszow, Sucharskiego 2, 35-225 Rzeszow, Poland
* Correspondence: dmytro.khylyuk@umlub.edu.pl (D.K.); dr_r_lesyk@org.lviv.net (R.L.)

Table of Contents

- Copies of ^{1}H and ^{13}C NMR spectra of compounds **1-19**..S1-S38.
- The cytotoxic effects of the investigated spiro-thiazolidinone–isatin conjugates **1-19** and doxorubicin…………………………………………………………………..S39.

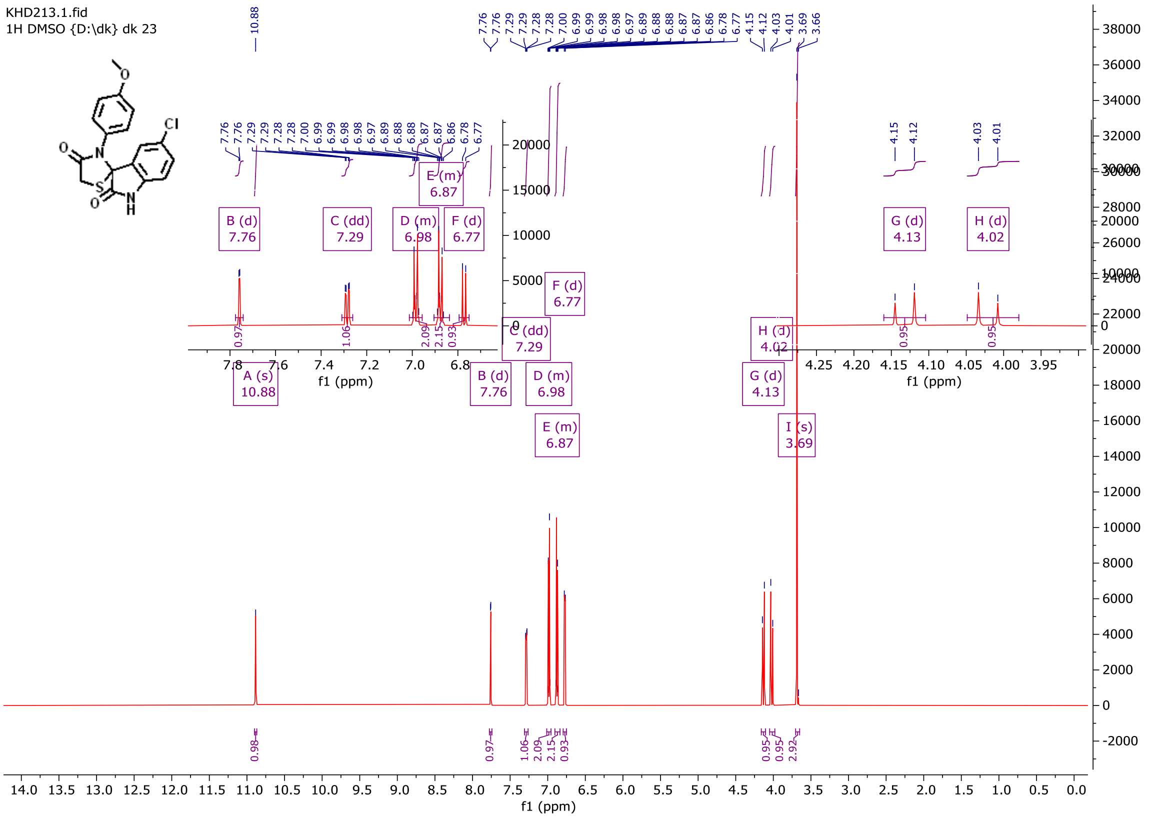

Figure S1. ^{1}H NMR spectrum of compound **1**

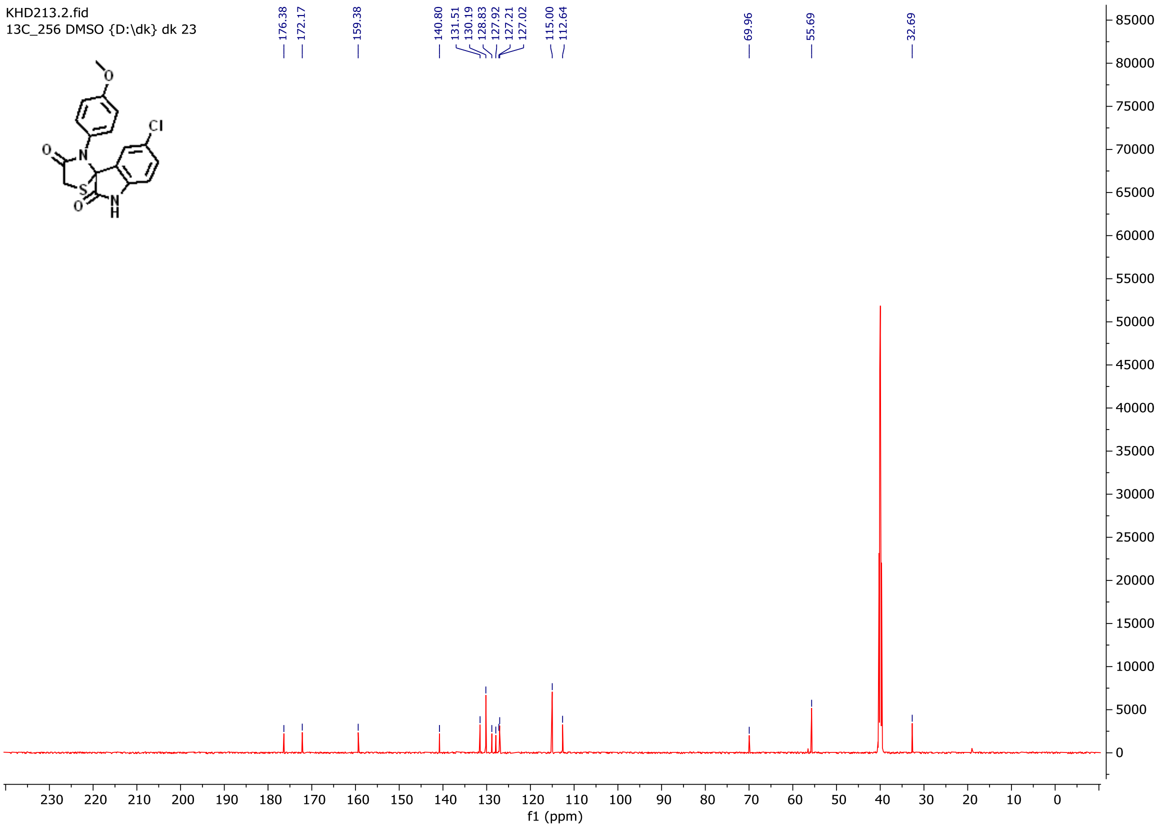

Figure S2. ^{13}C NMR spectrum of compound **1**

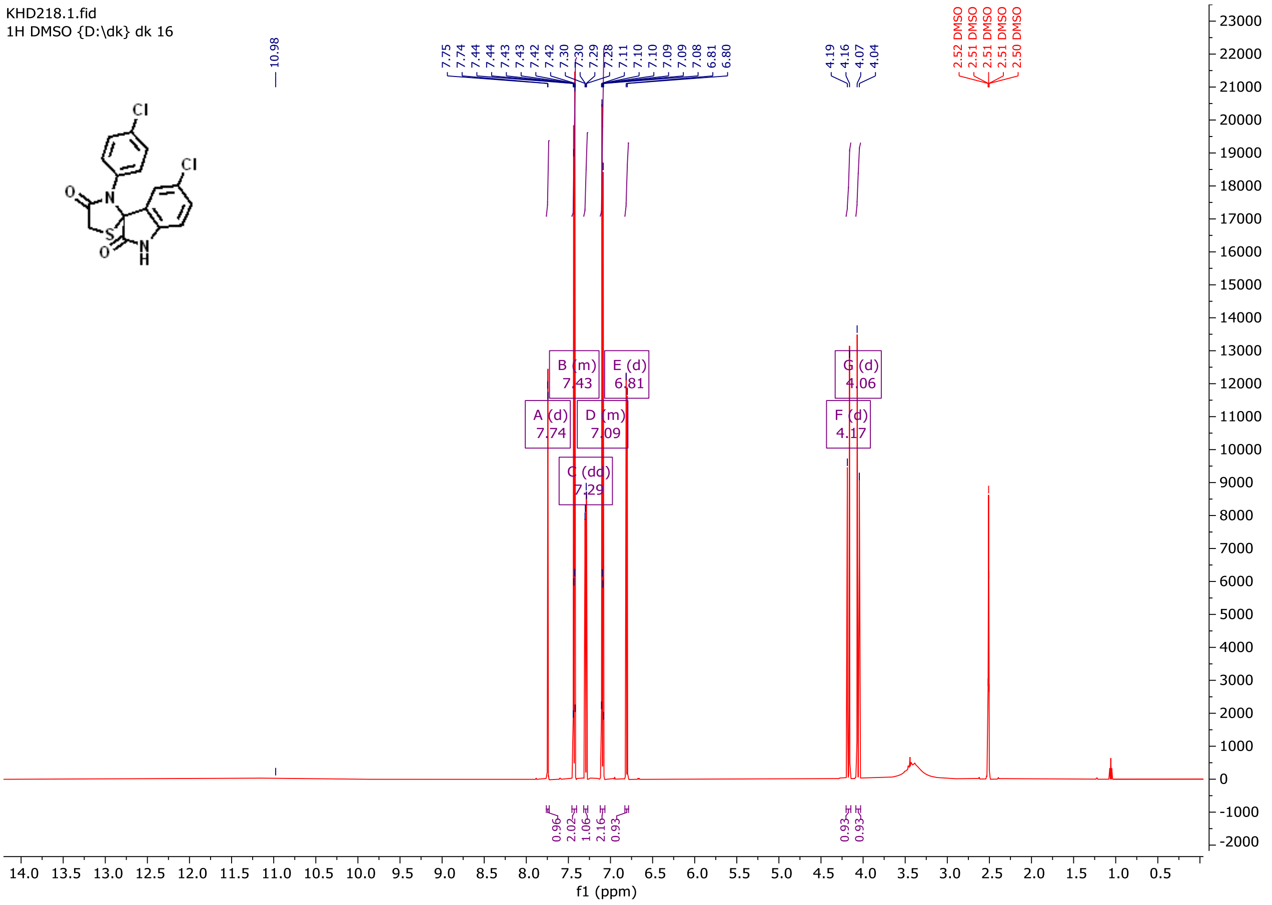

Figure S3. ^{1}H NMR spectrum of compound **2**

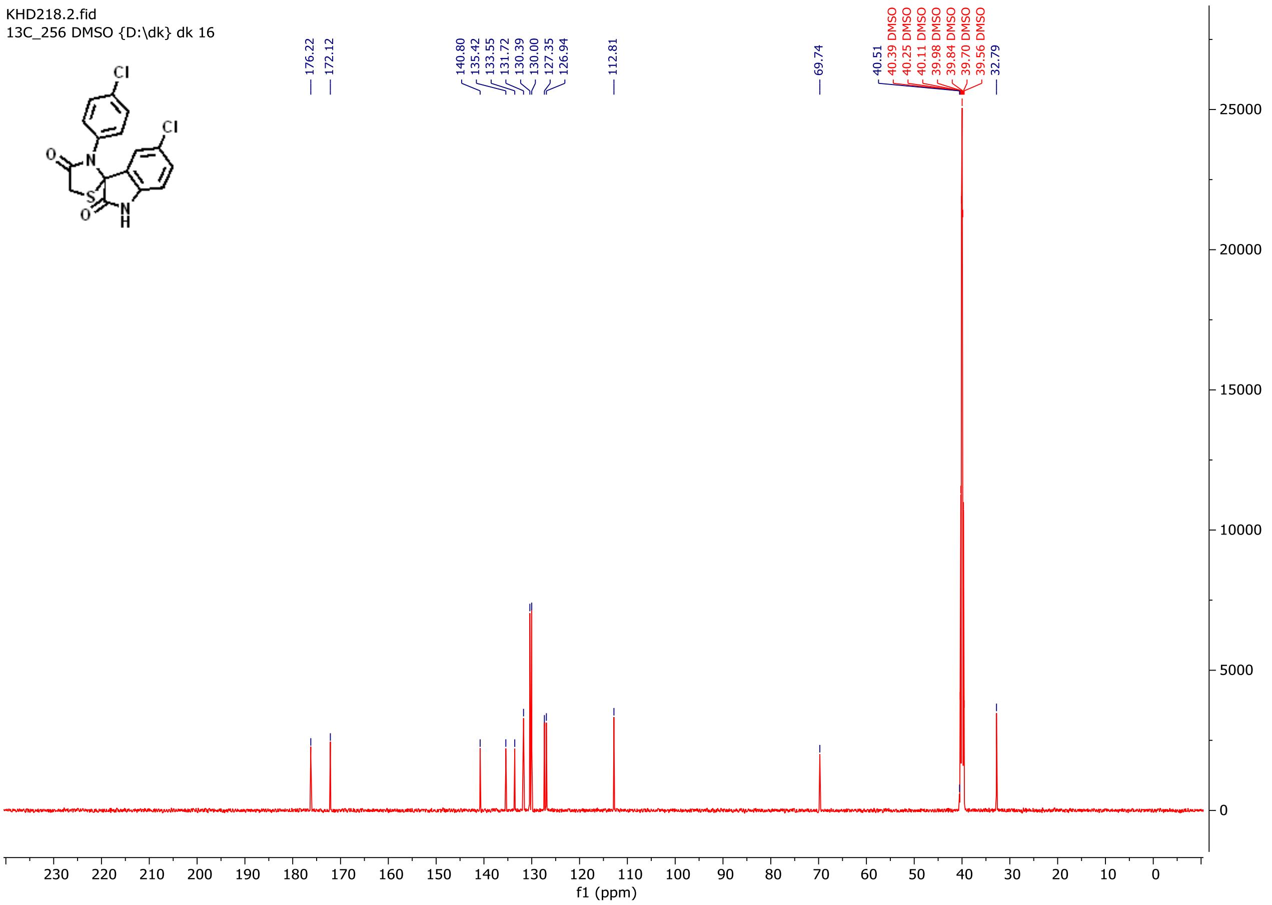

Figure S4. [13C] NMR spectrum of compound **2**

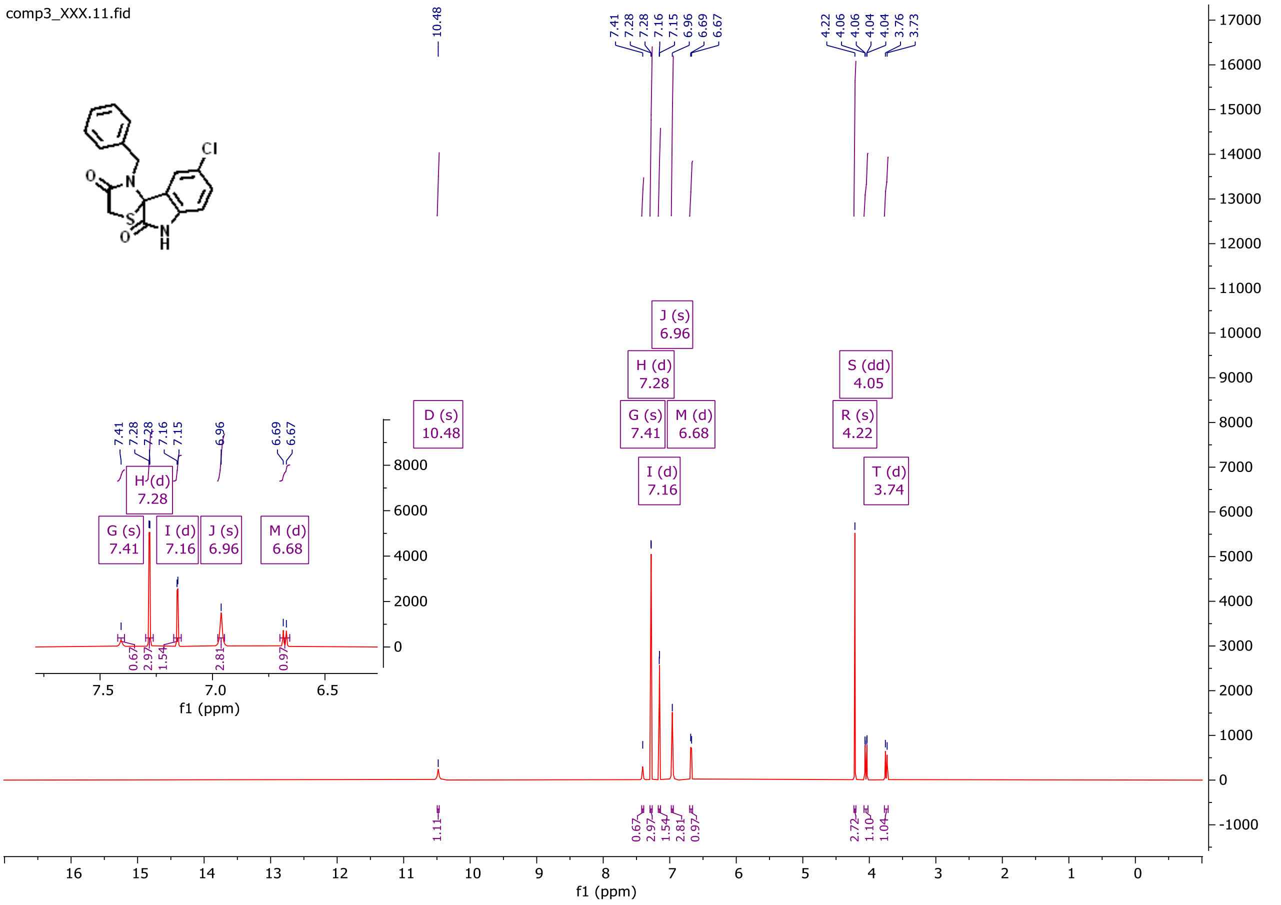

Figure S5. ^{1}H NMR spectrum of compound **3**

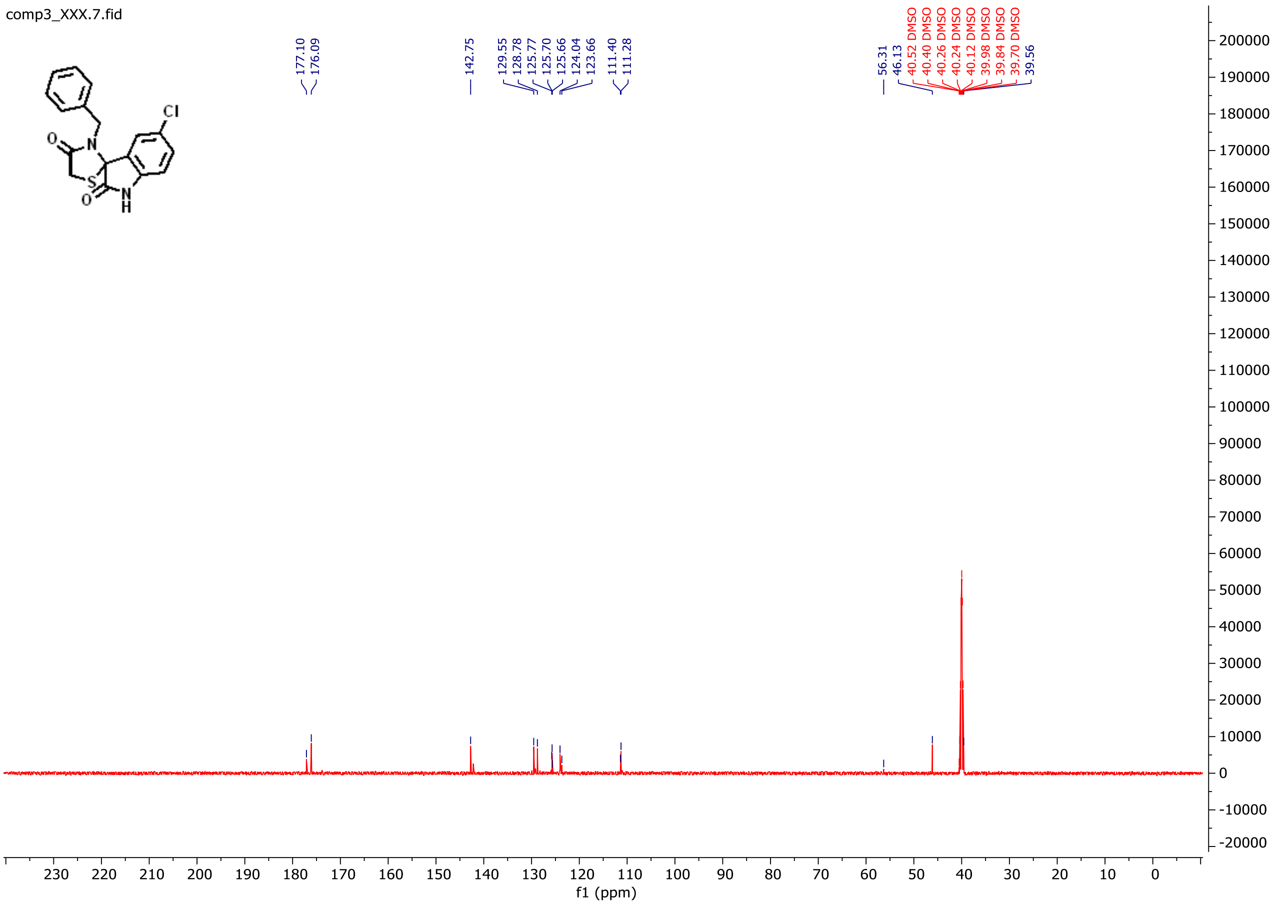

Figure S6 ^{13}C NMR spectrum of compound **3**

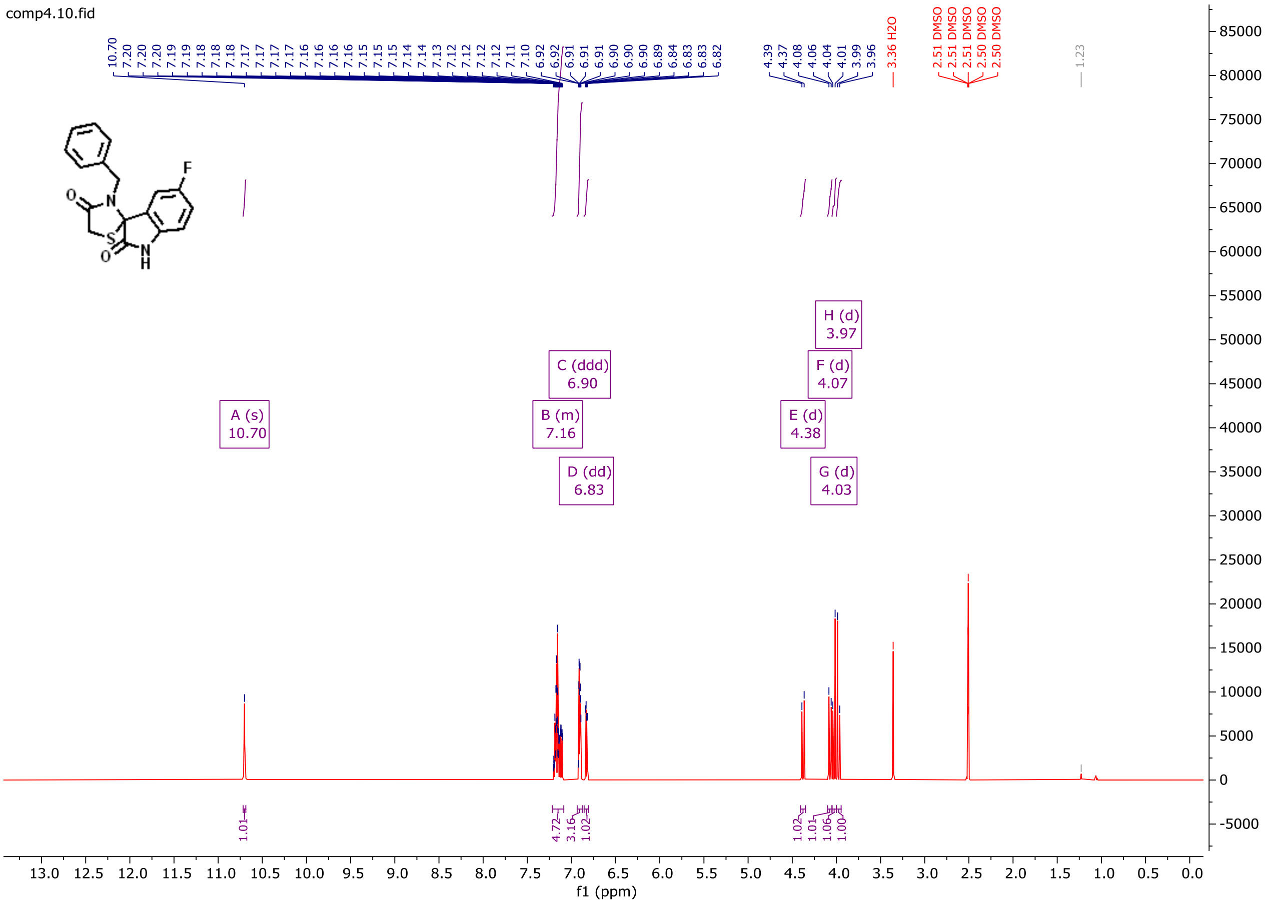

Figure S7. ^{1}H NMR spectrum of compound **4**

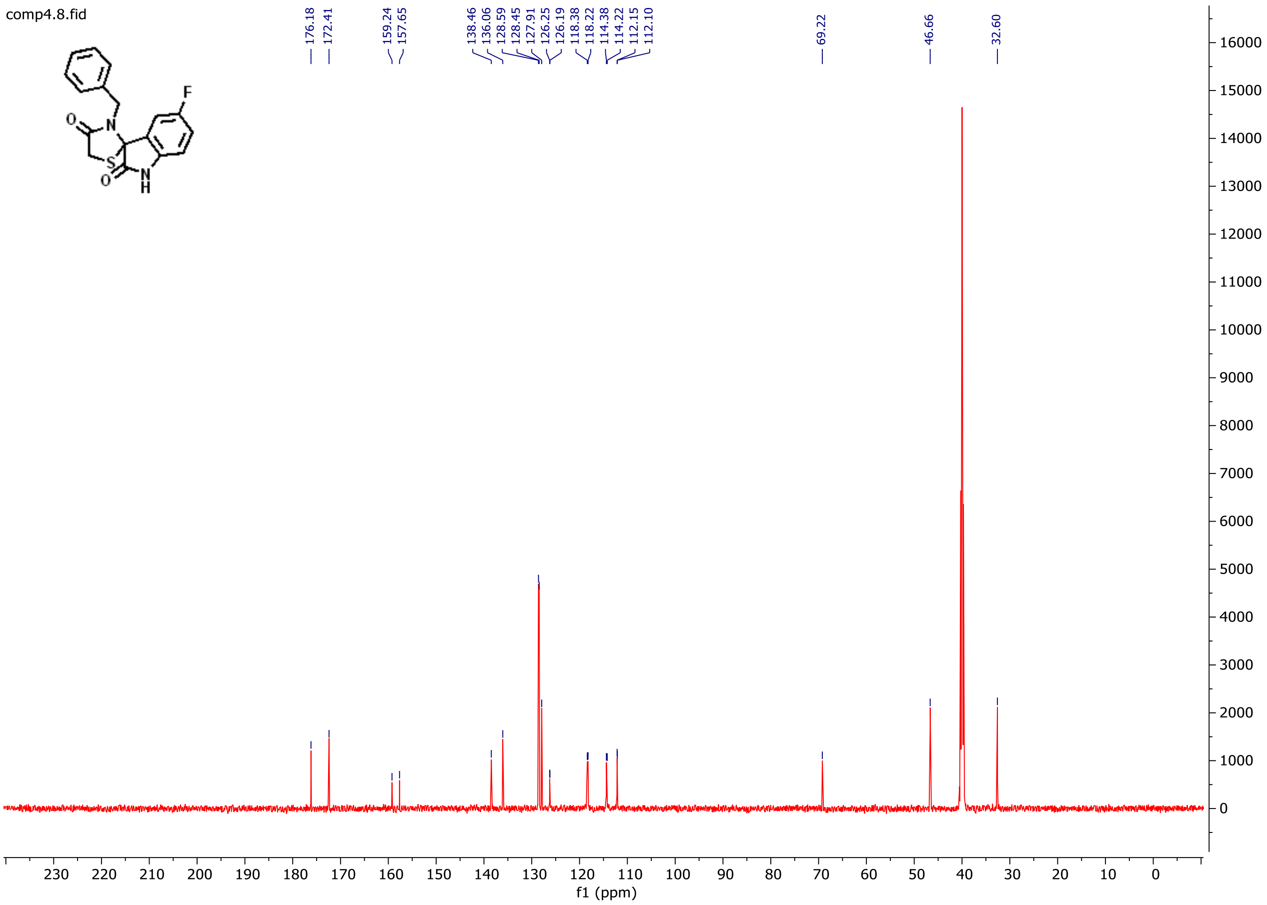

Figure S8. ^{13}C NMR spectrum of compound **4**

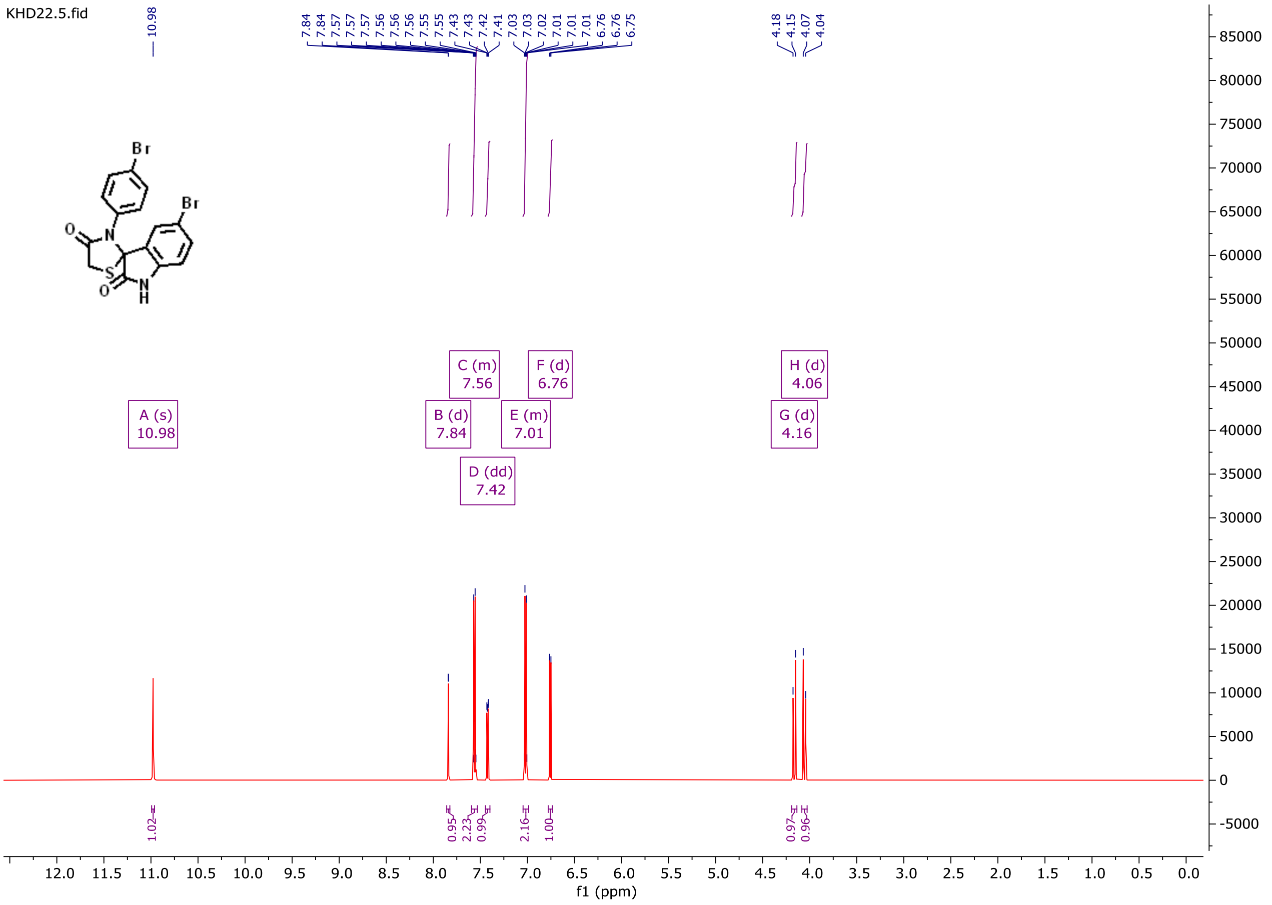

Figure S9. ^{1}H NMR spectrum of compound **5**

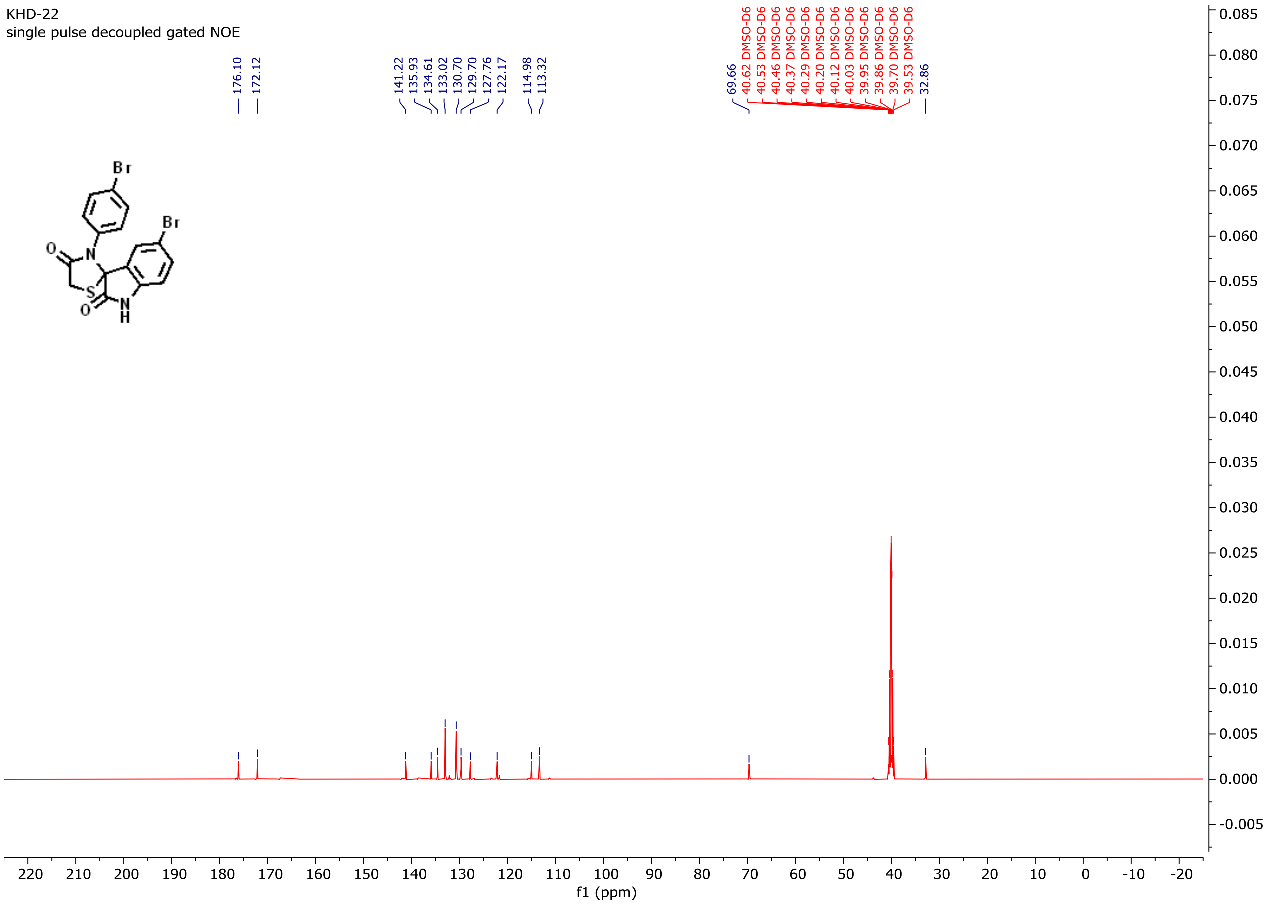

Figure S10. ^{13}C NMR spectrum of compound **5**

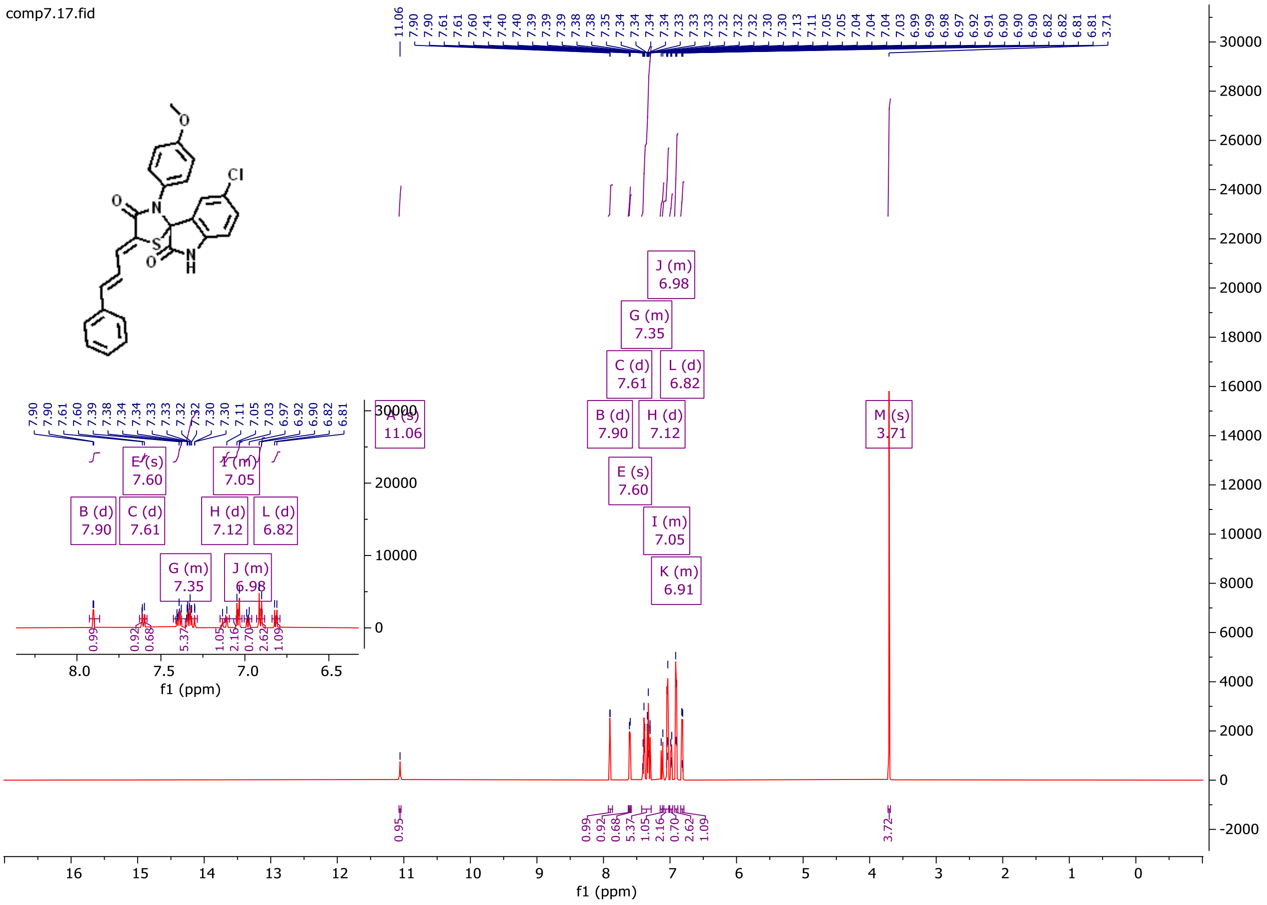

Figure S11. 1H NMR spectrum of compound **6**

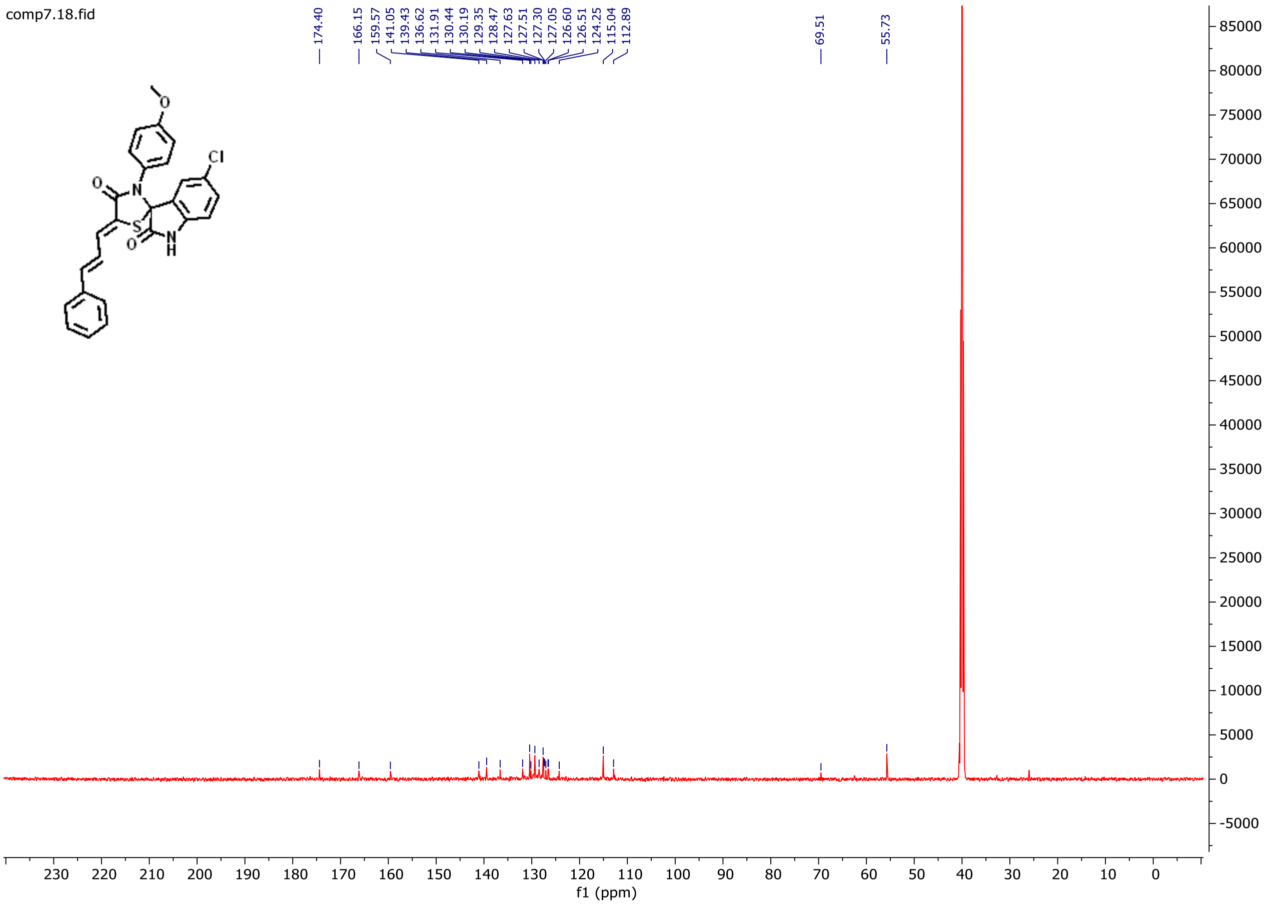

Figure S12. 13C NMR spectrum of compound **6**.

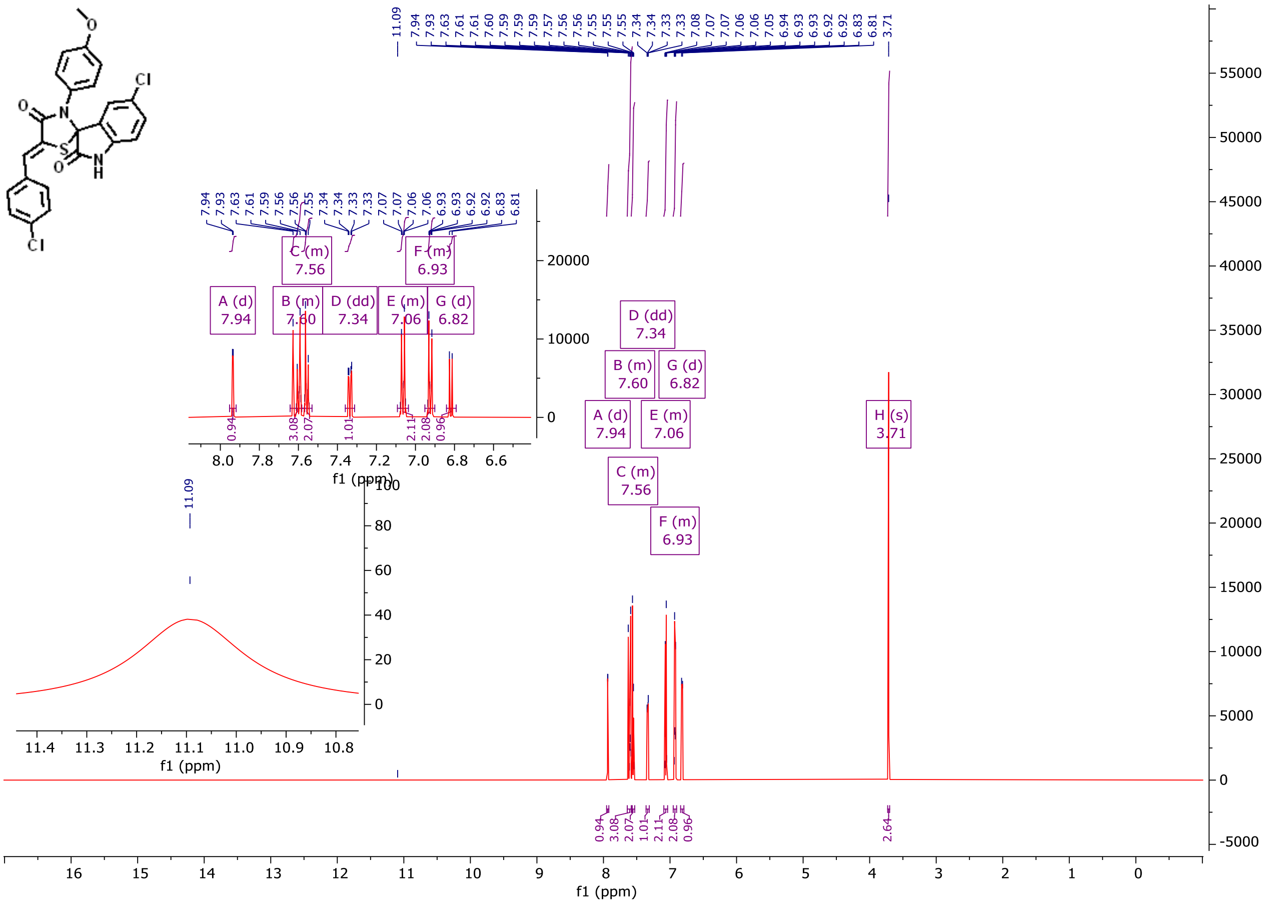

Figure S13. ^{1}H NMR spectrum of compound **7**

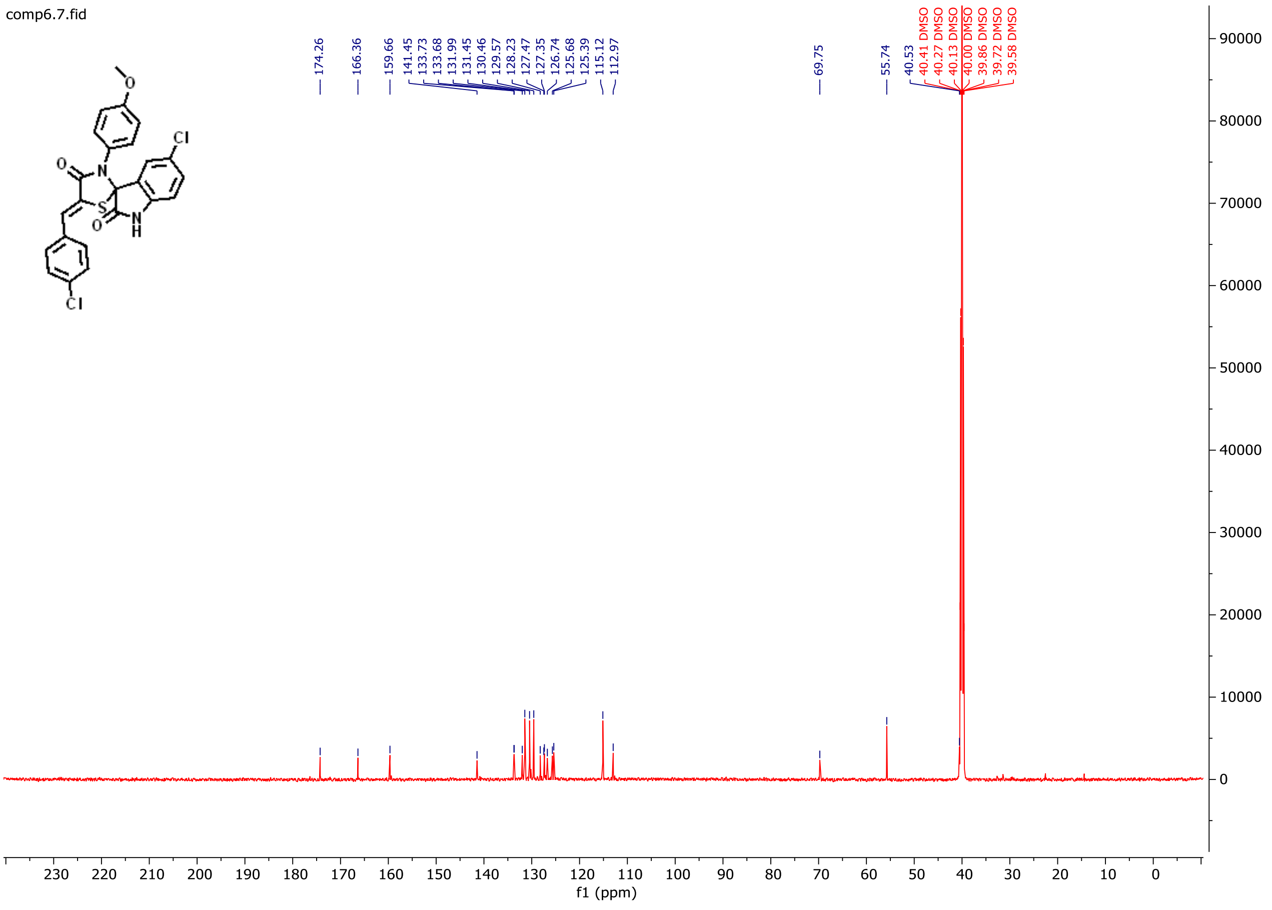

Figure S14. ^{13}C NMR spectrum of compound **7**

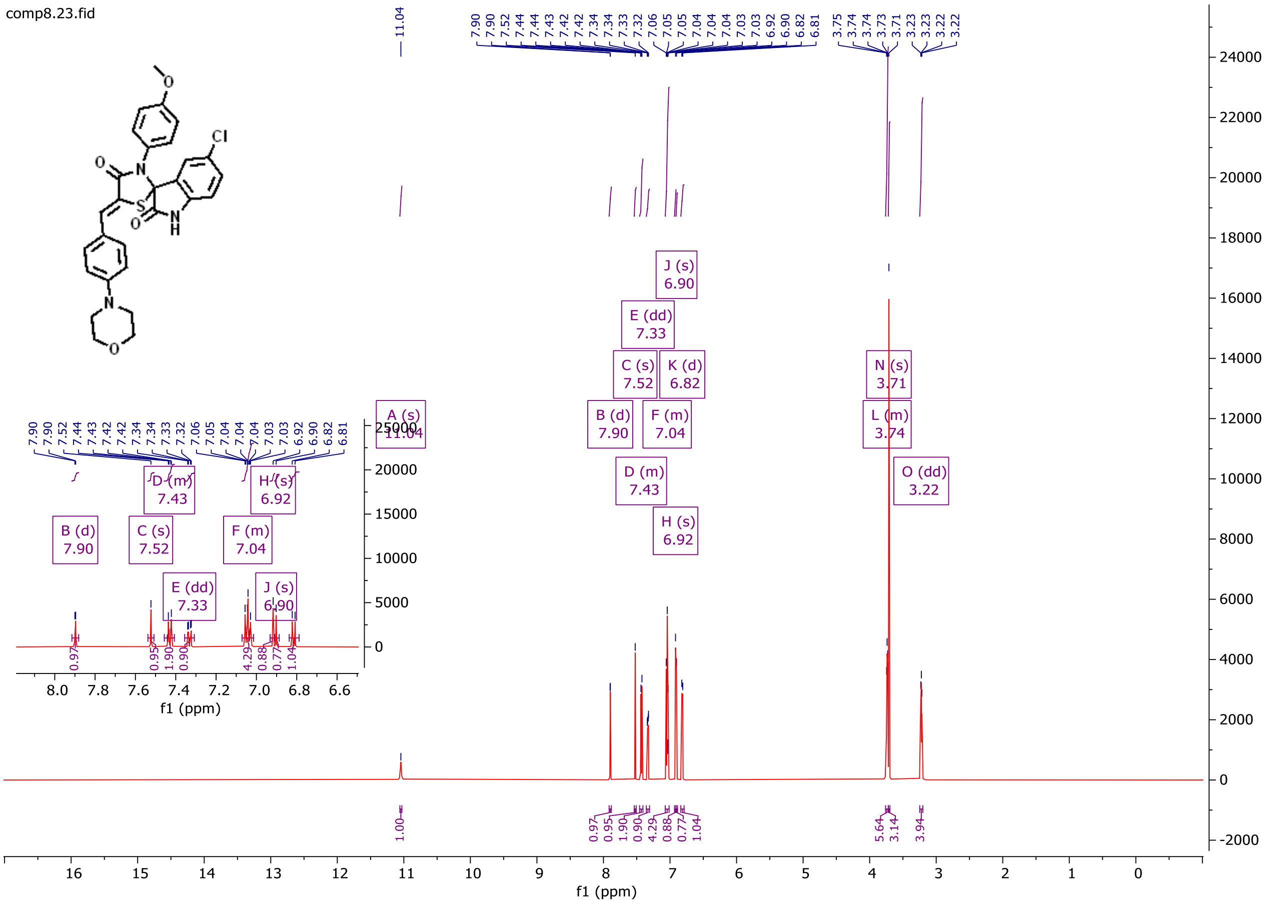

Figure S15. ^{1}H NMR spectrum of compound **8**

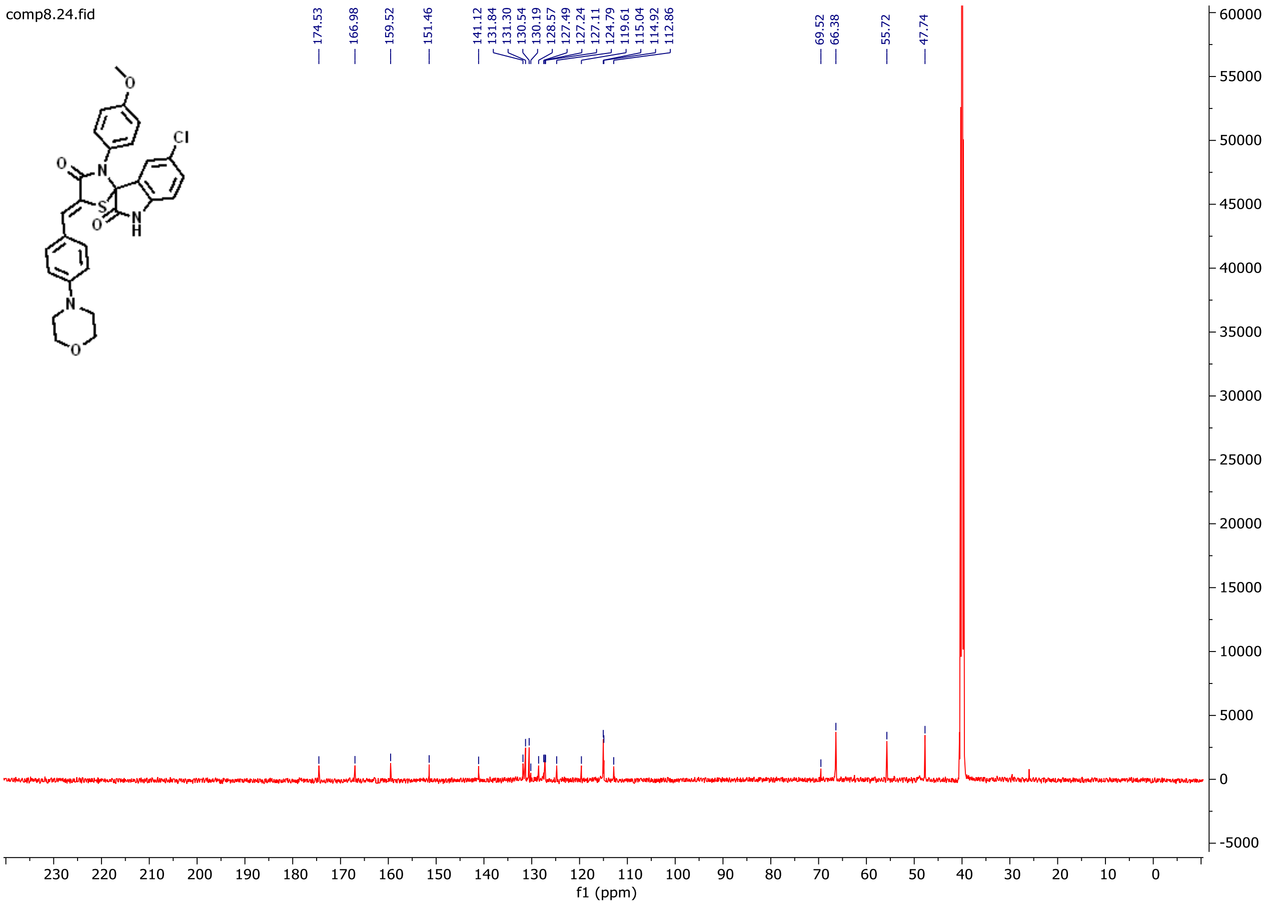

Figure S16. ^{13}C NMR spectrum of compound **8**

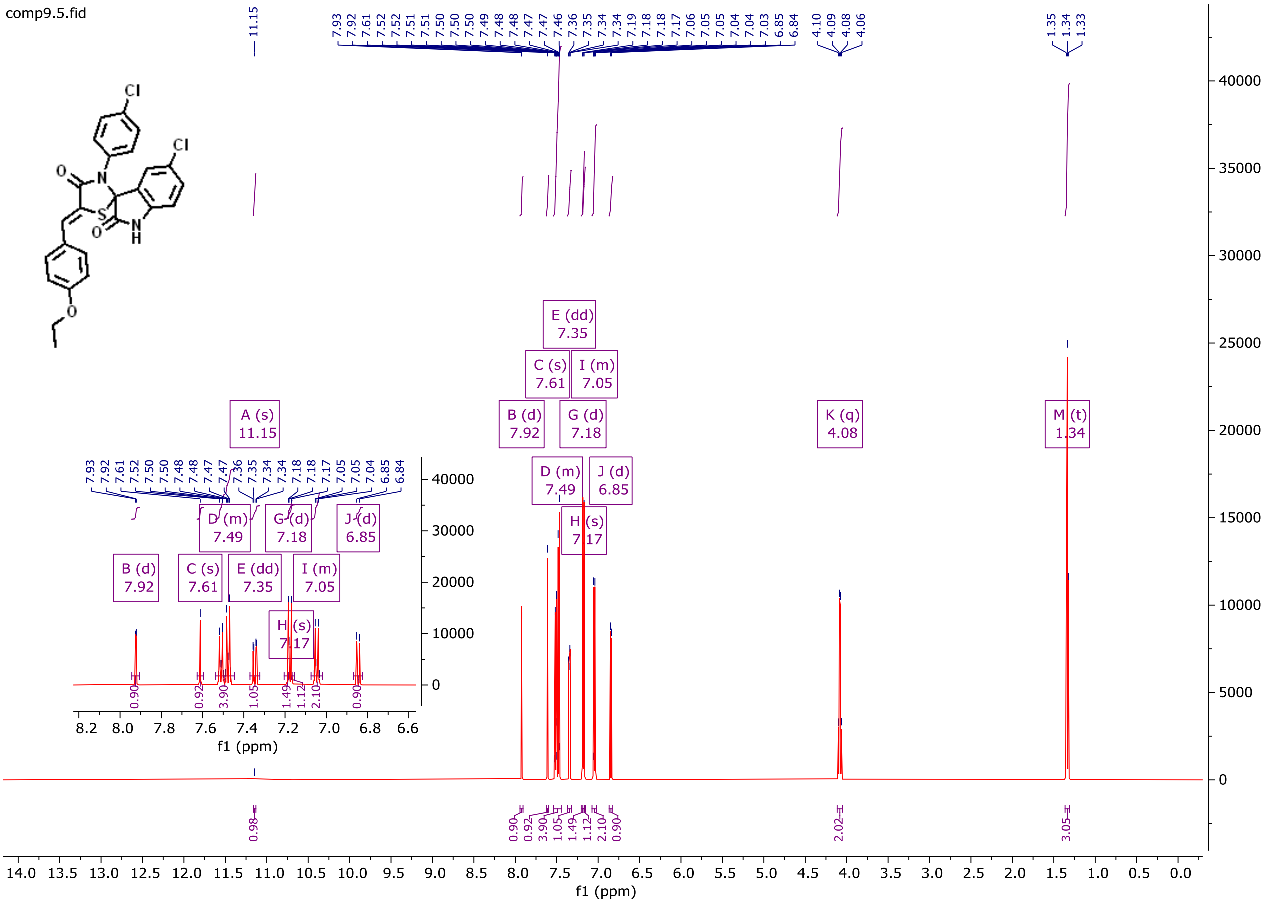

Figure S17. ^{1}H NMR spectrum of compound **9**

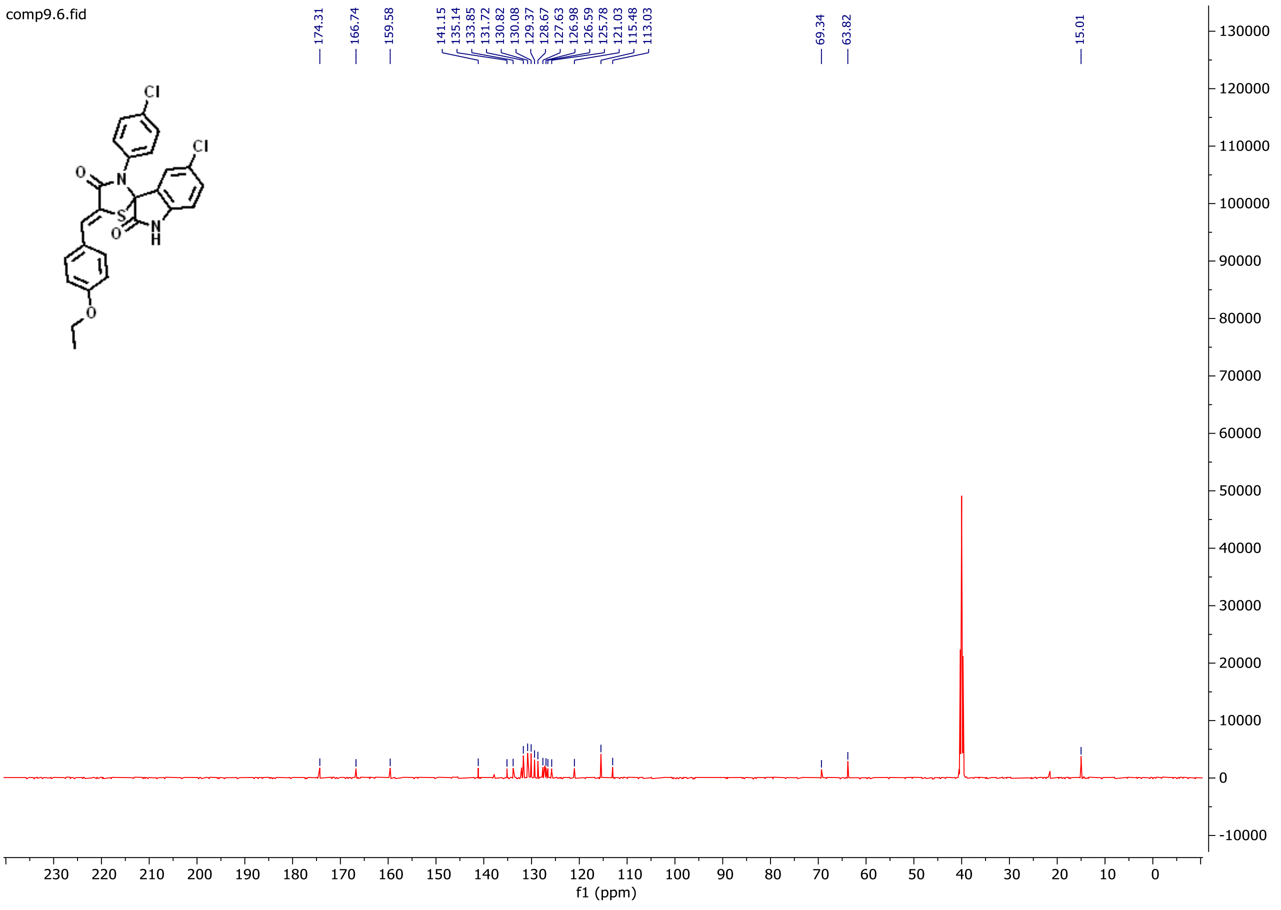

Figure S18. ^{13C} NMR spectrum of compound **9**

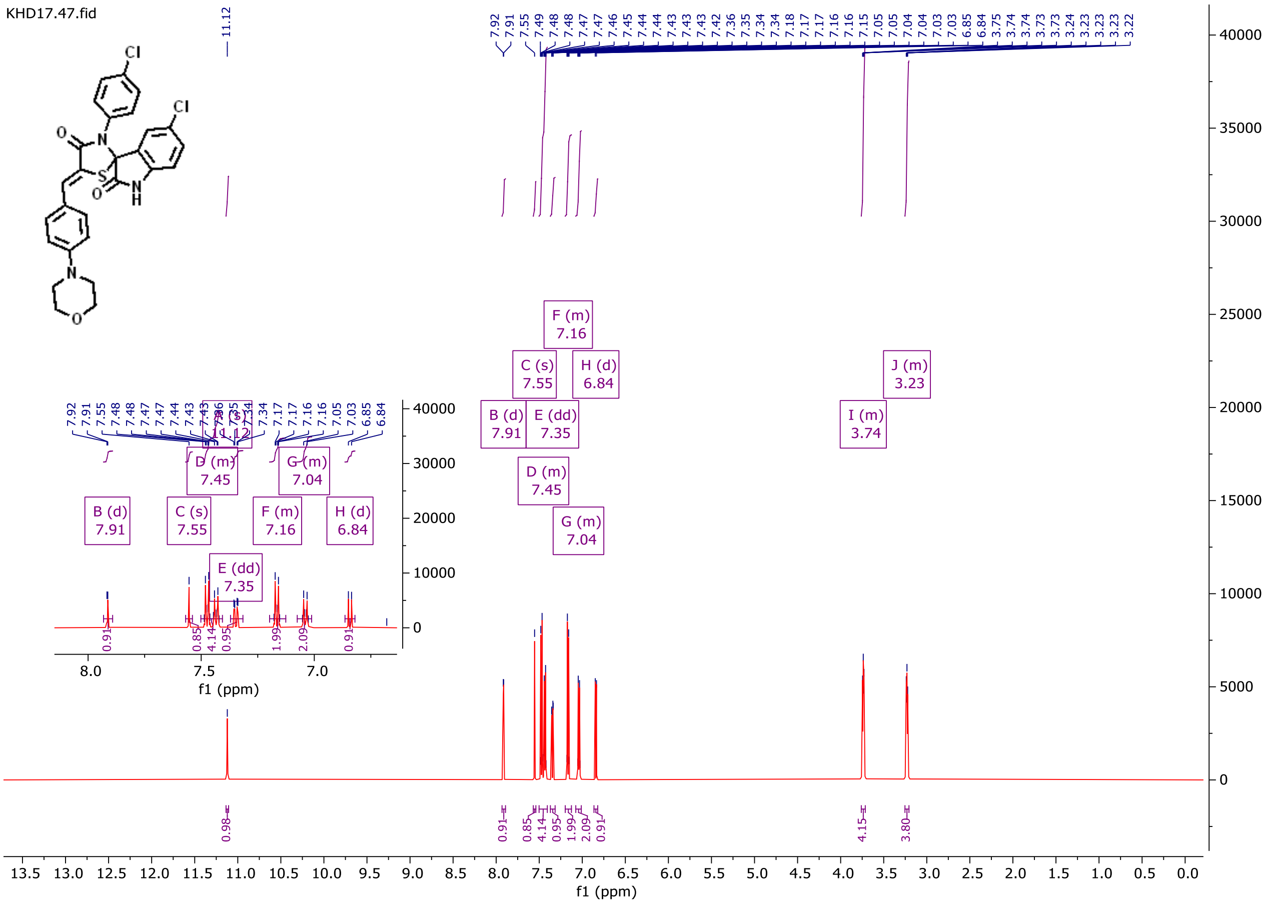

Figure S19. ^{1}H NMR spectrum of compound **10**

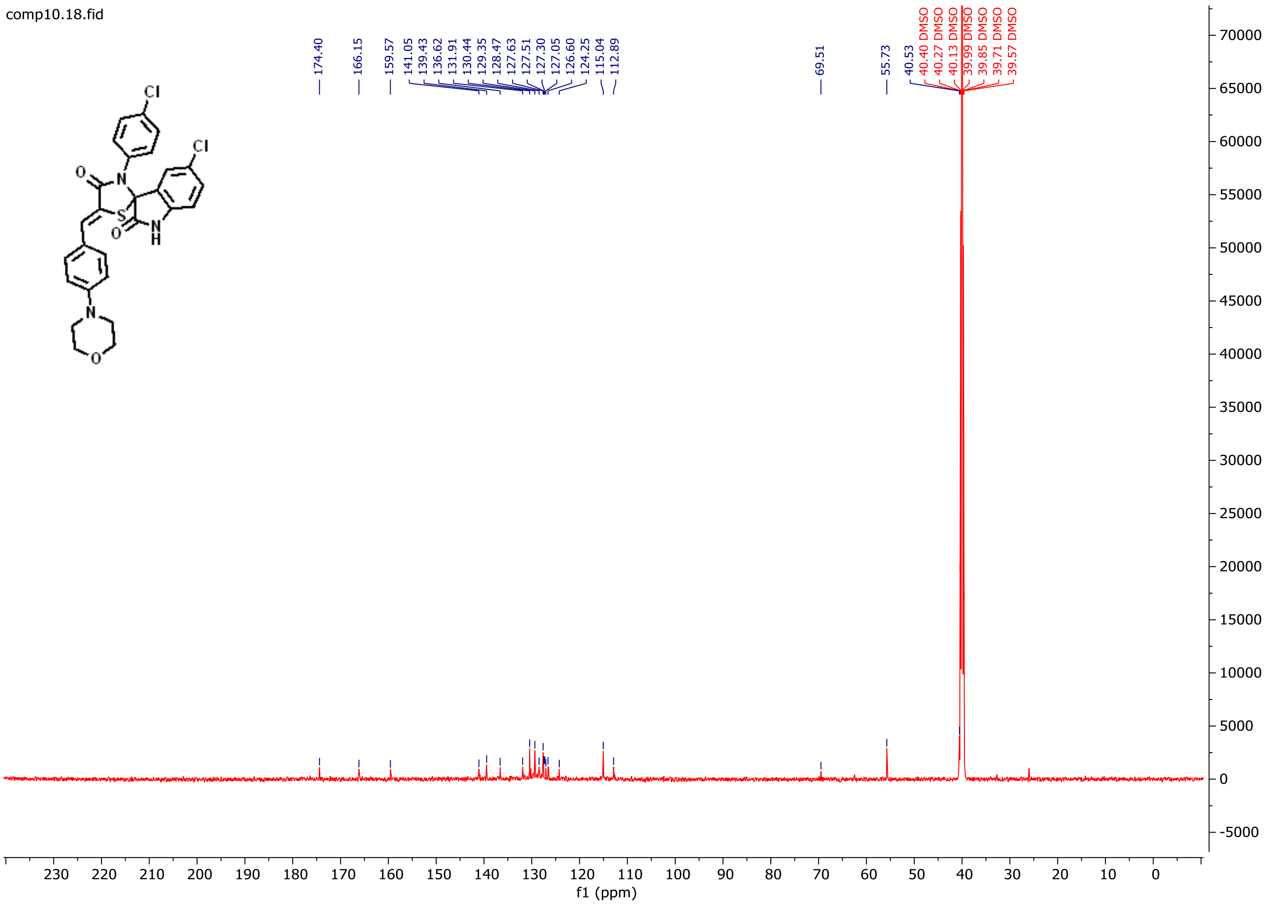

Figure S20. ^{13}C NMR spectrum of compound **10**

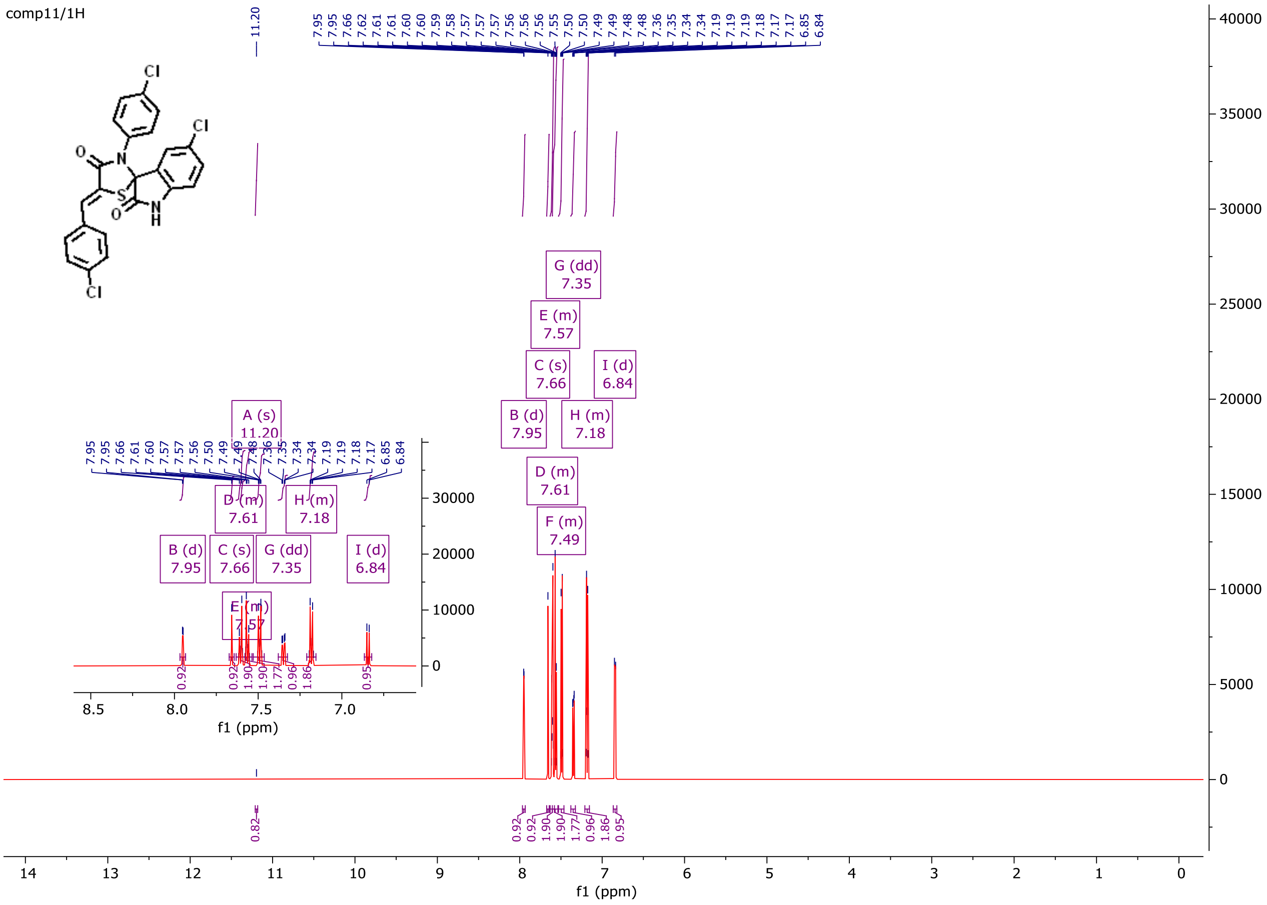

Figure S21. ^{1}H NMR spectrum of compound **11**

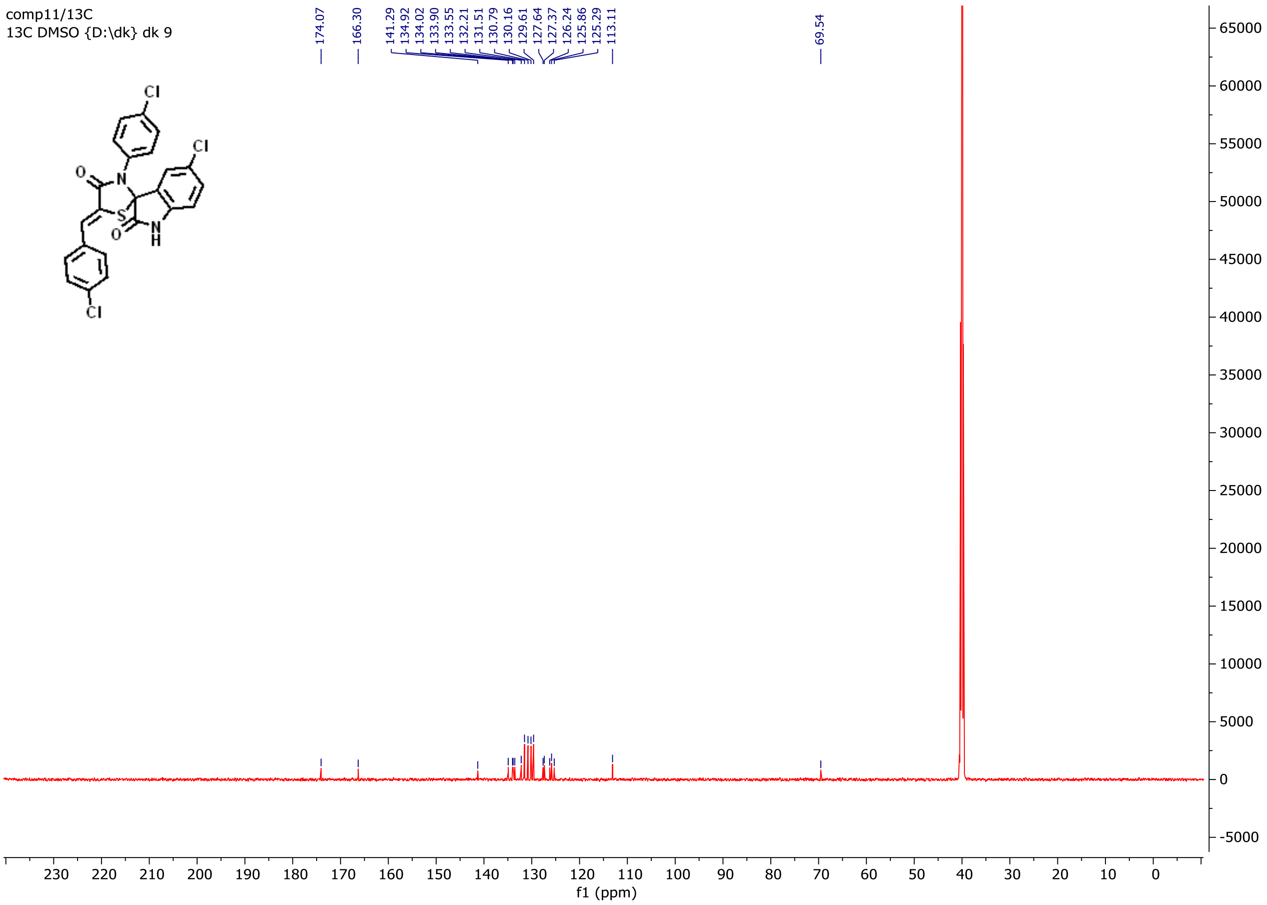

Figure S22. ^{13}C NMR spectrum of compound **11**

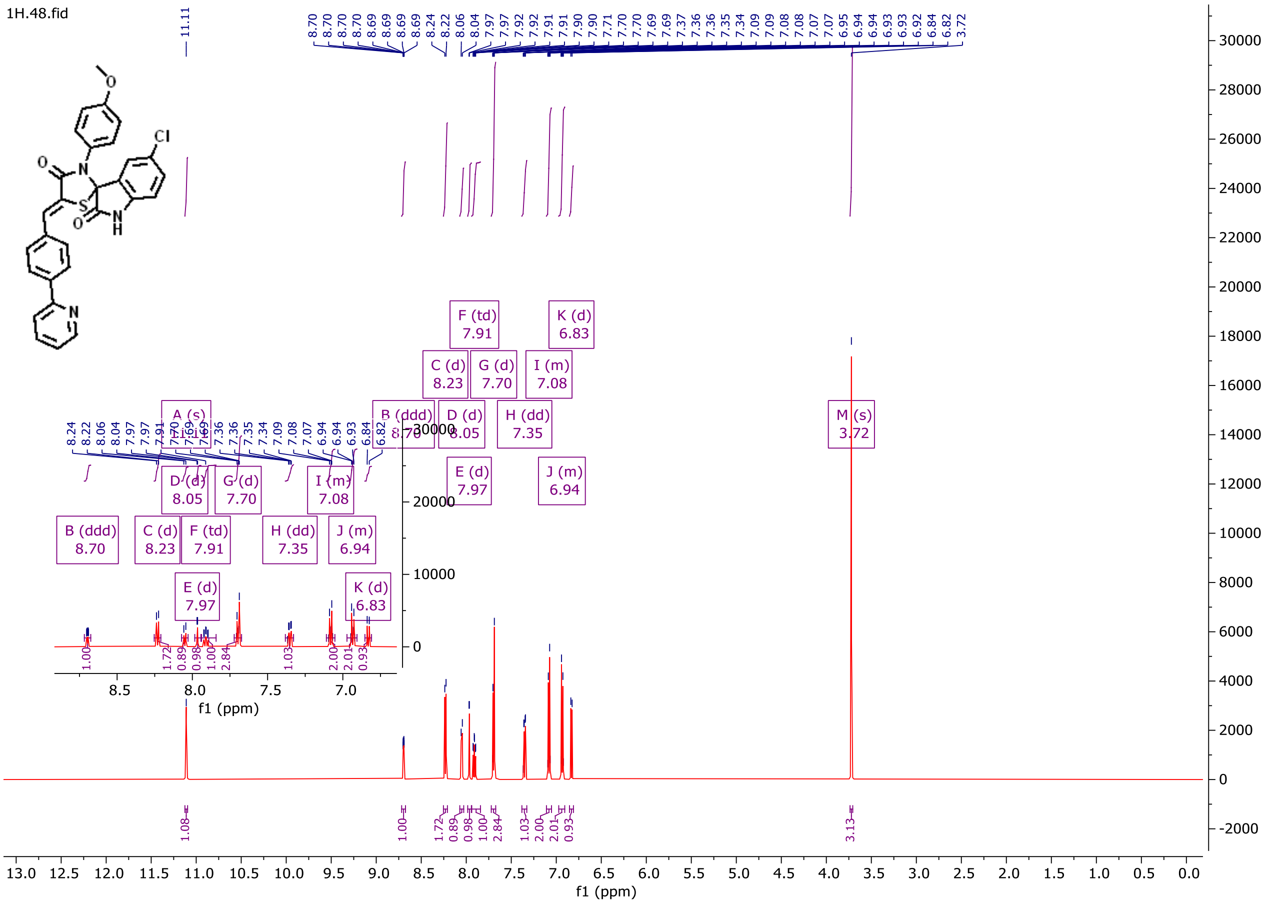

Figure S23. 1H NMR spectrum of compound **12**

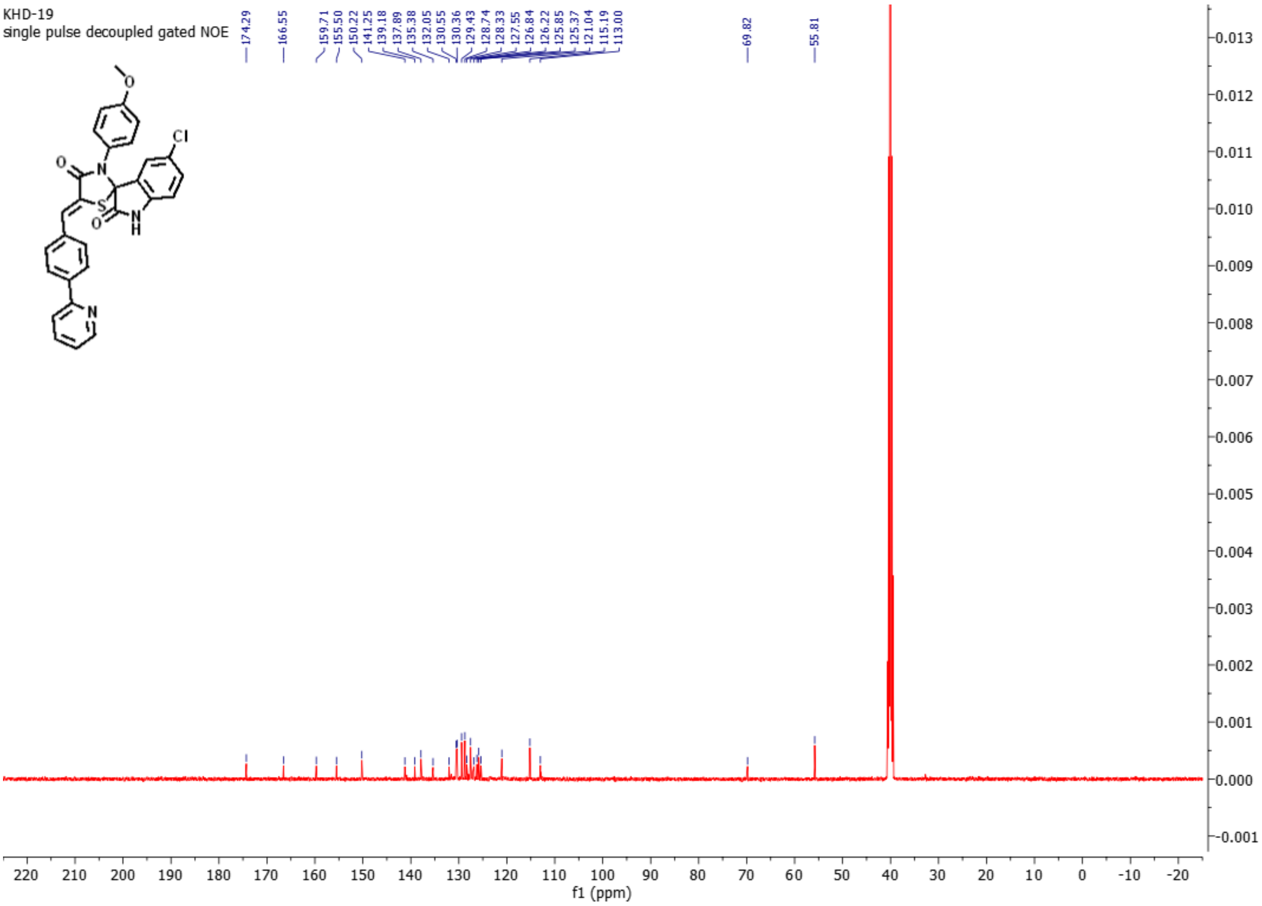

Figure S24. 13C NMR spectrum of compound **12**

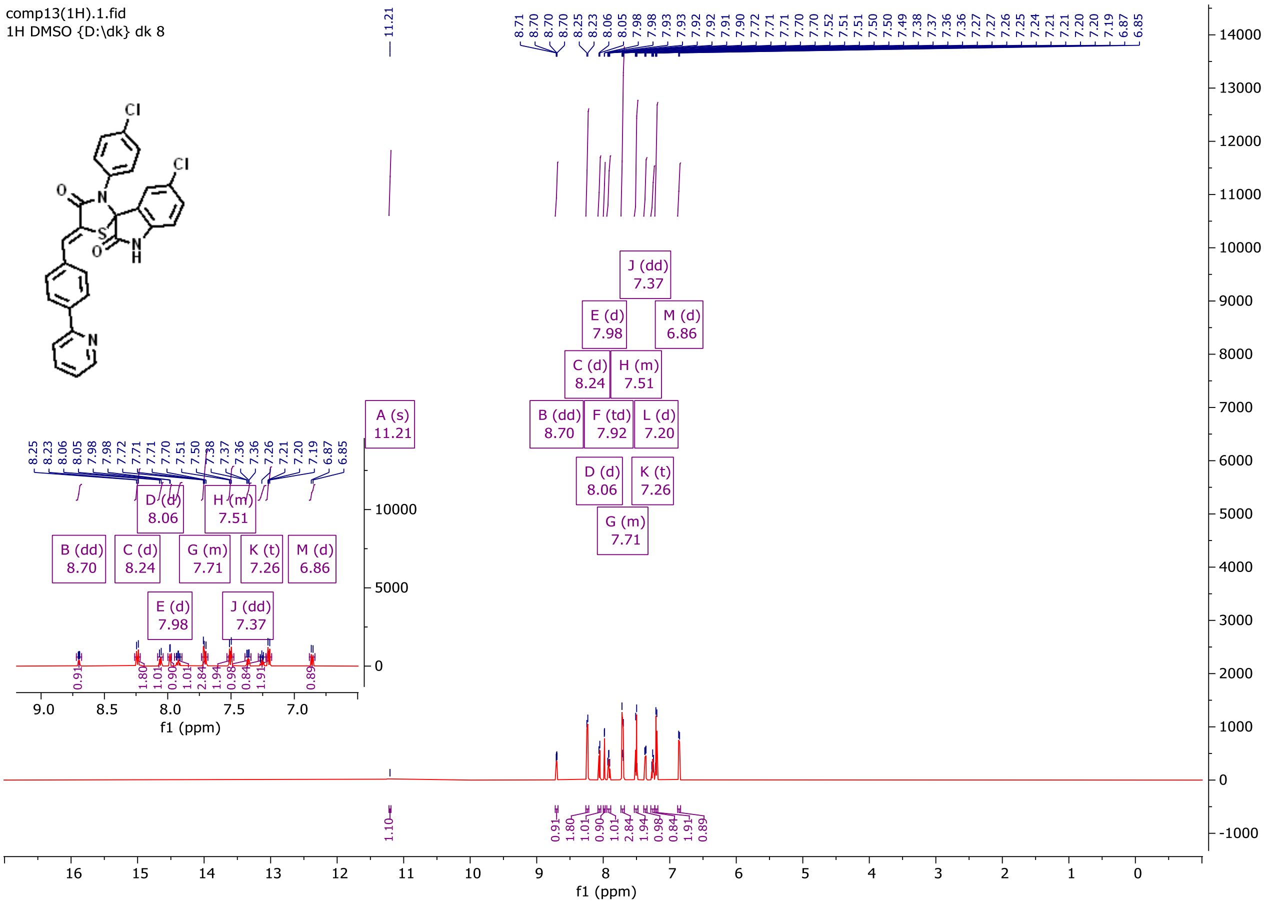

Figure S25. 1H NMR spectrum of compound **13**

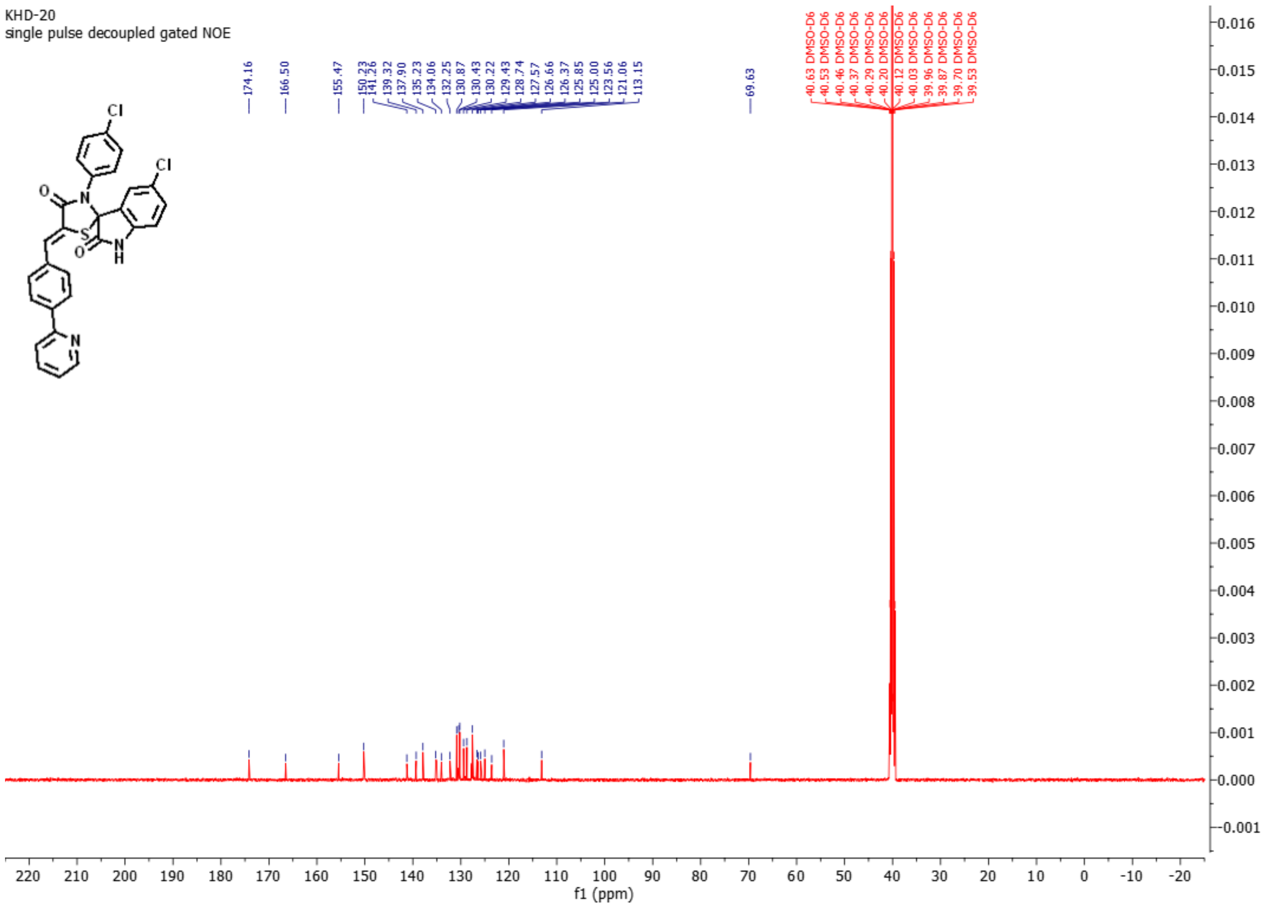

Figure S26. 13C NMR spectrum of compound **13**

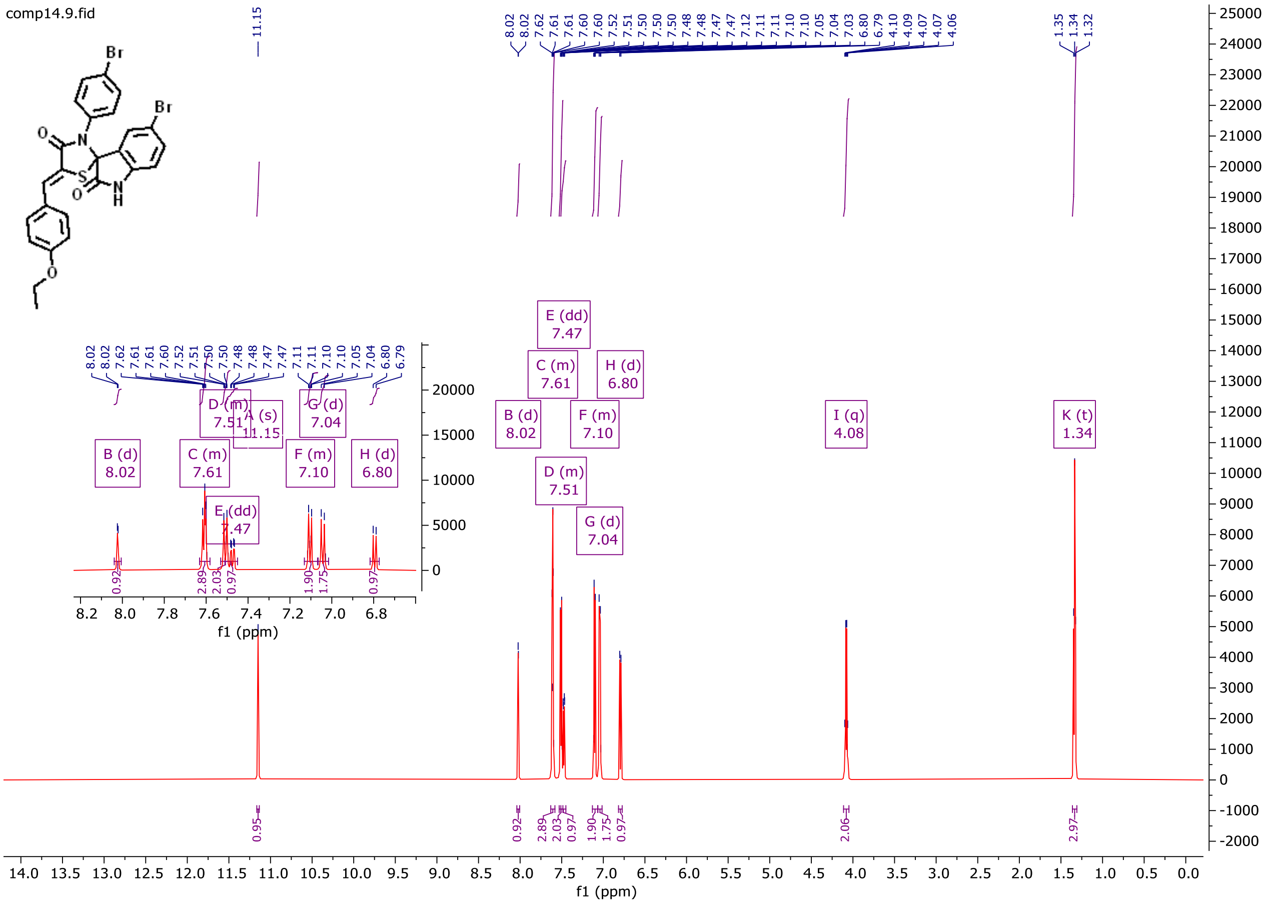

Figure S27. 1H NMR spectrum of compound **14**

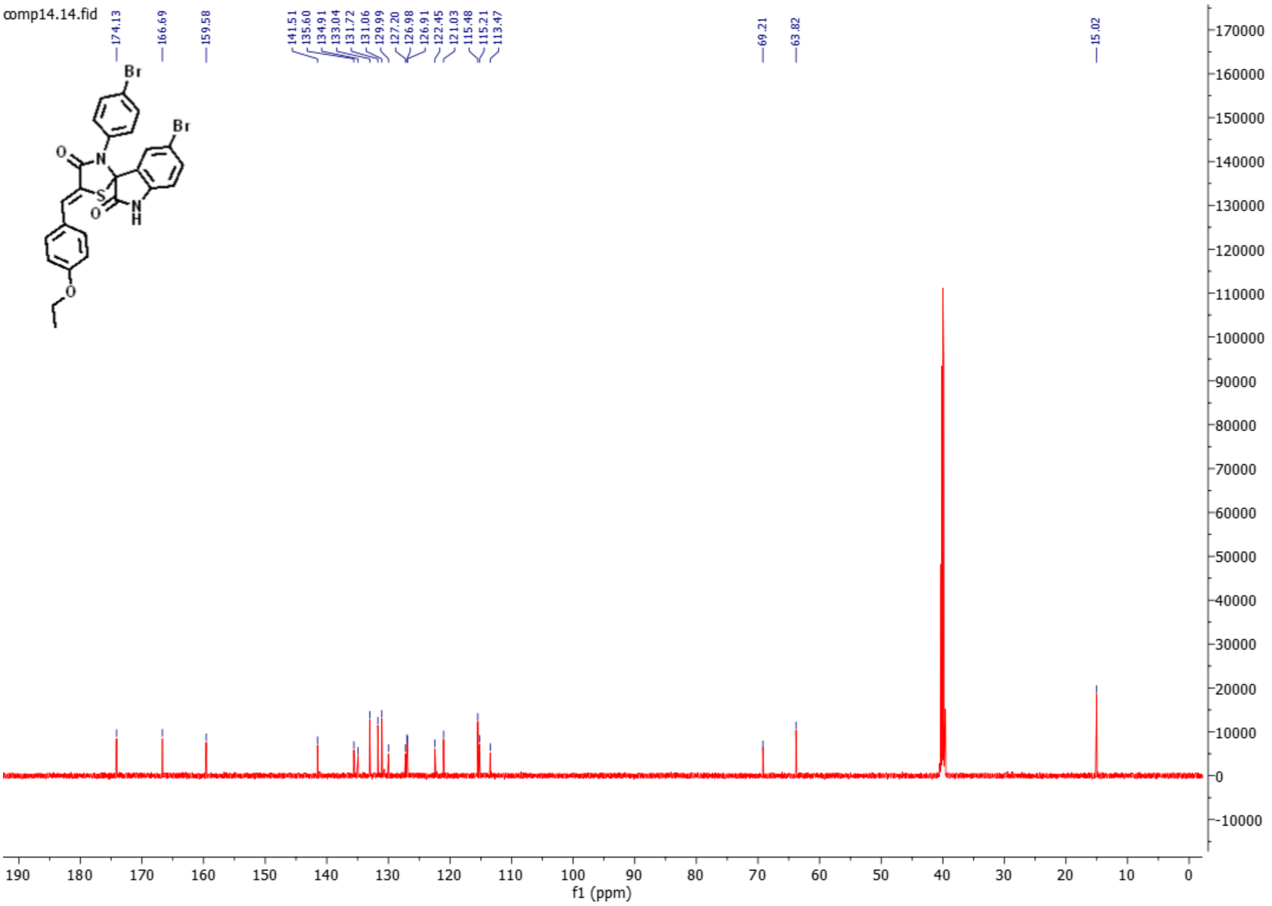

Figure S28. 13C NMR spectrum of compound **14**

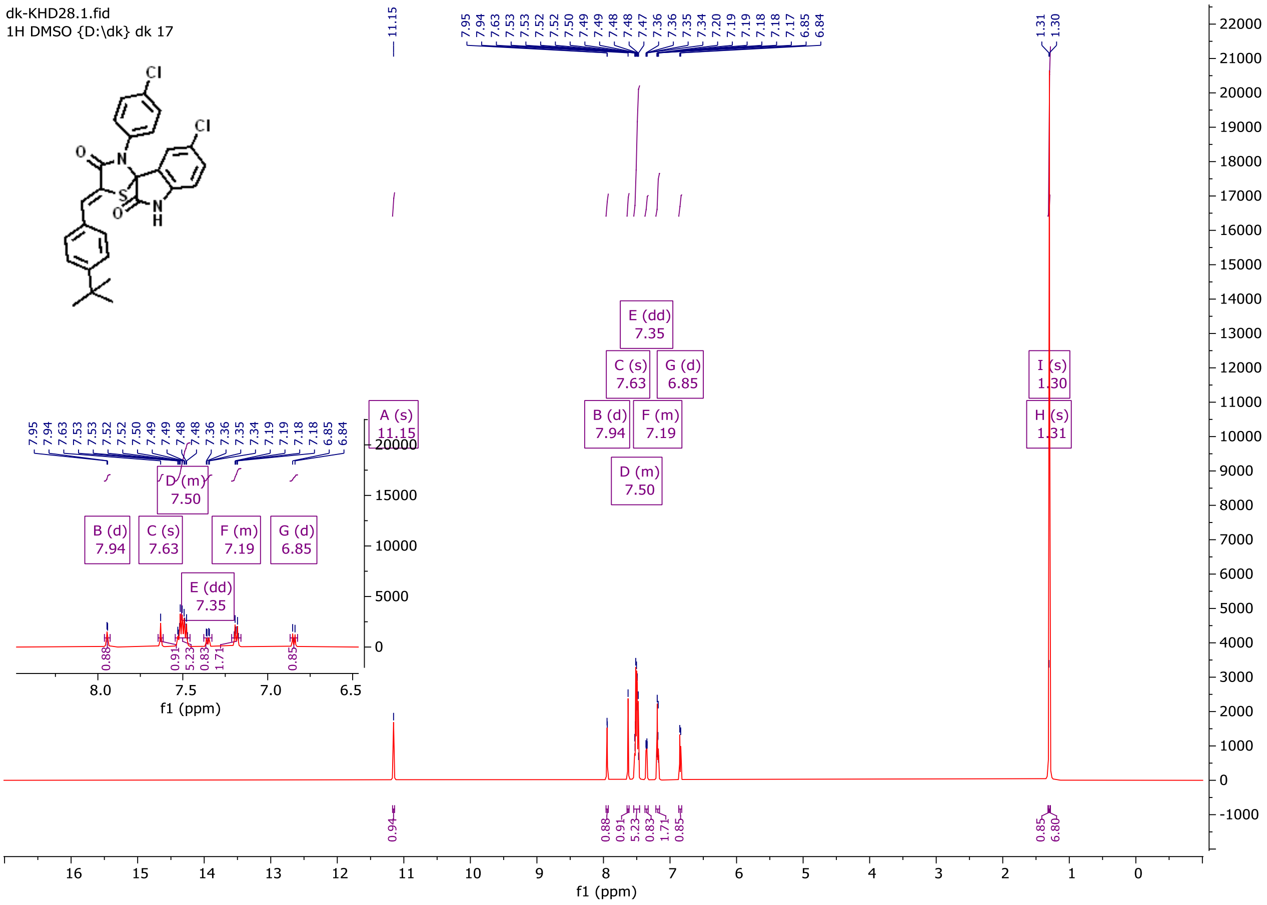

Figure S29. 1H NMR spectrum of compound **15**

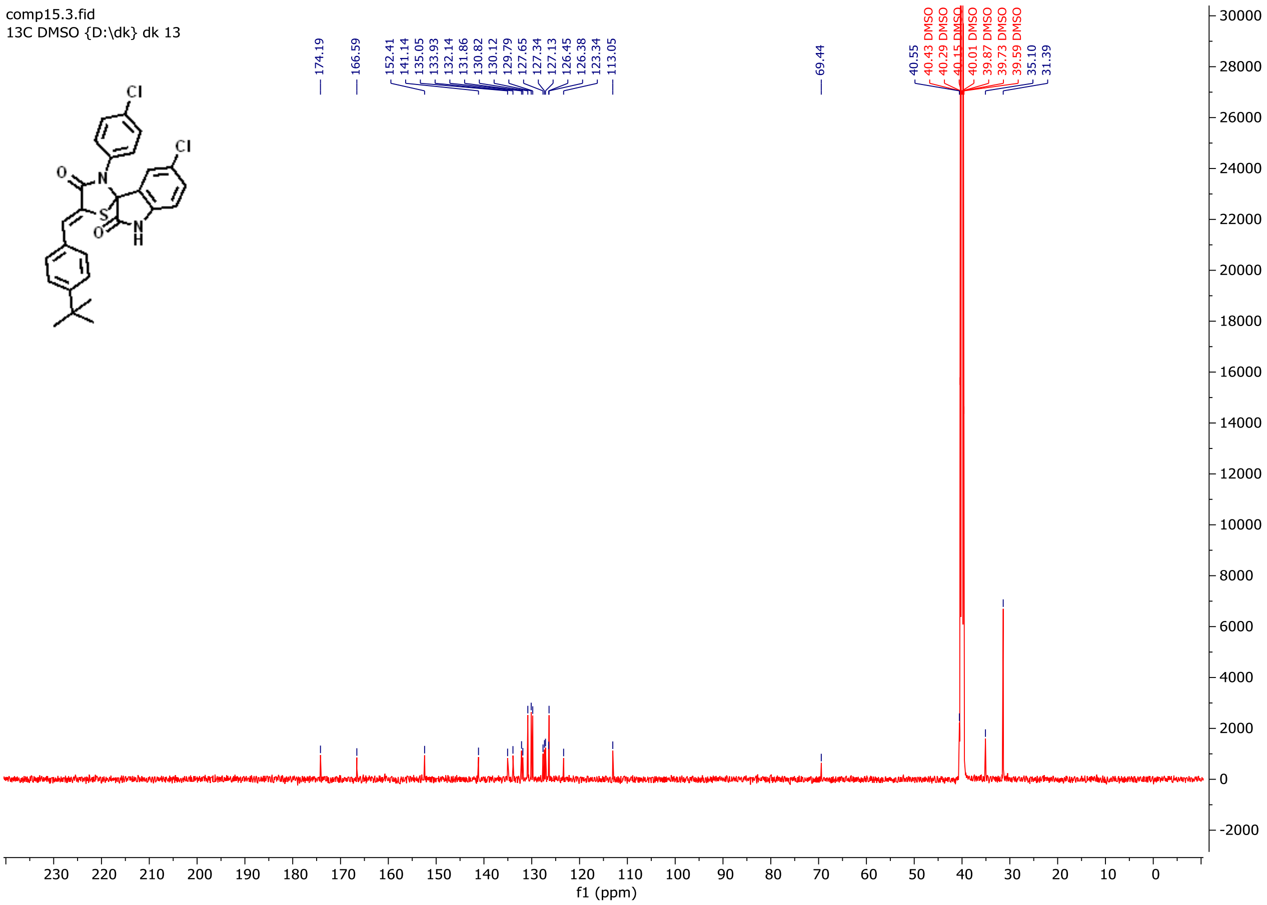

Figure S30. 13C NMR spectrum of compound **15**

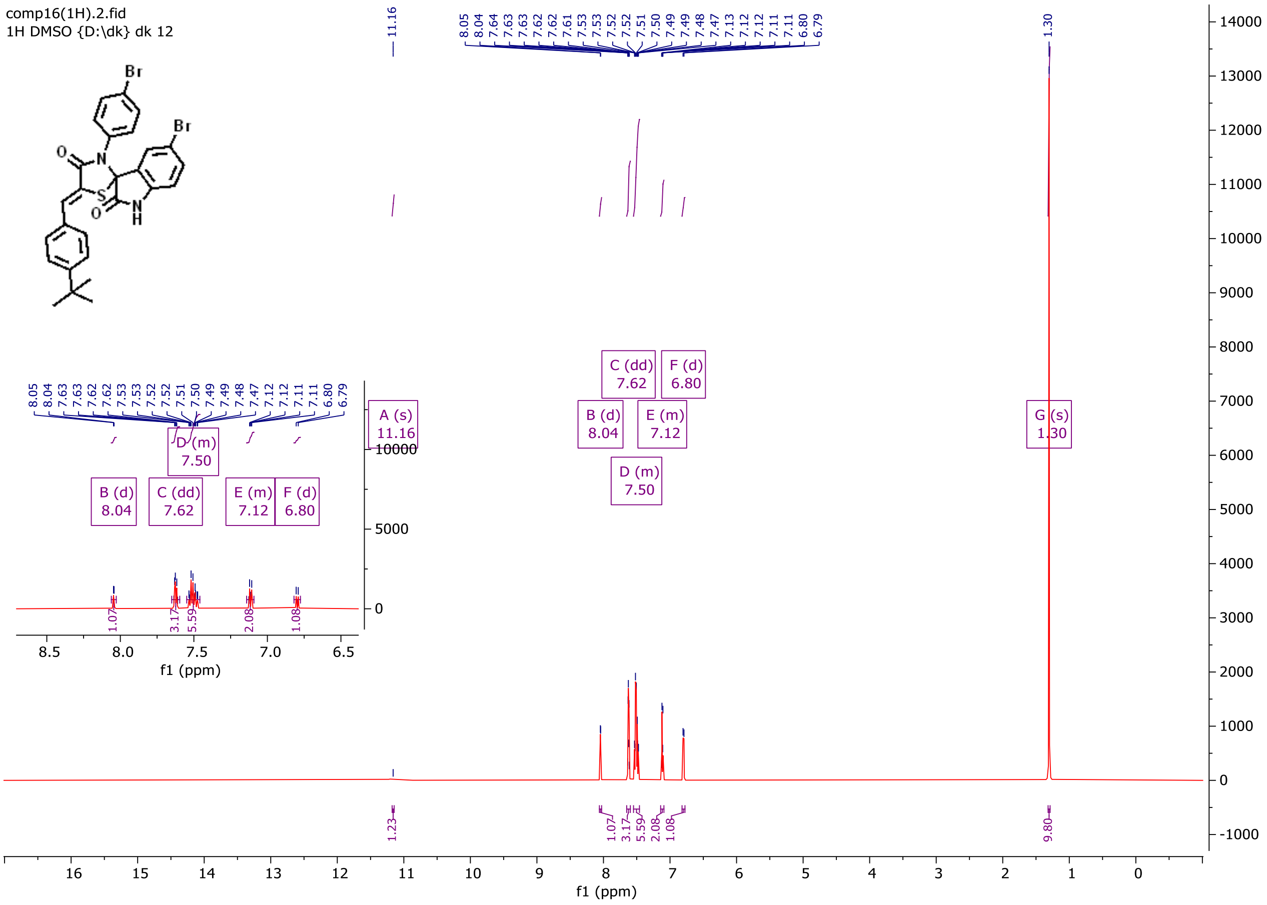

Figure S31. 1H NMR spectrum of compound **16**

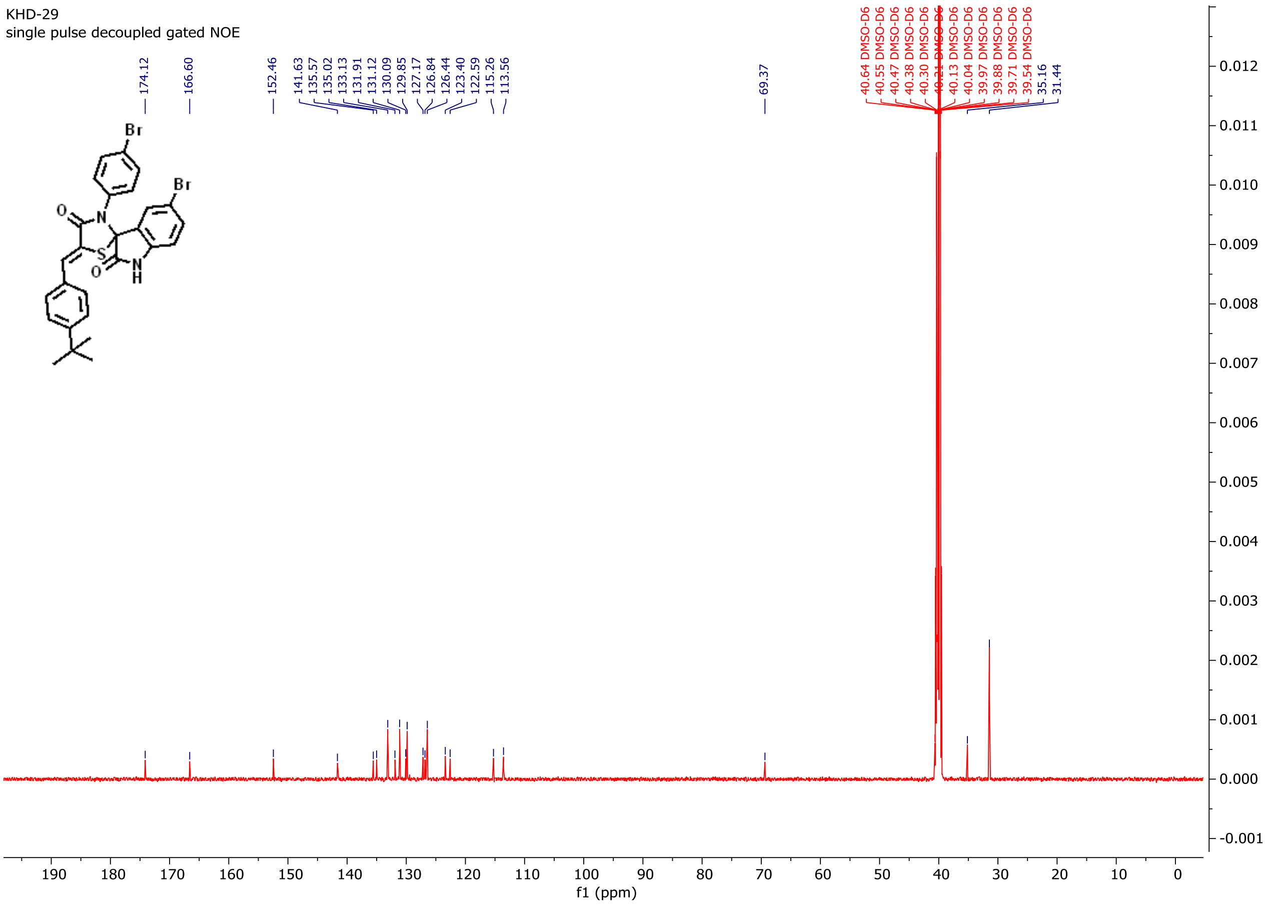

Figure S32. 13C NMR spectrum of compound **16**

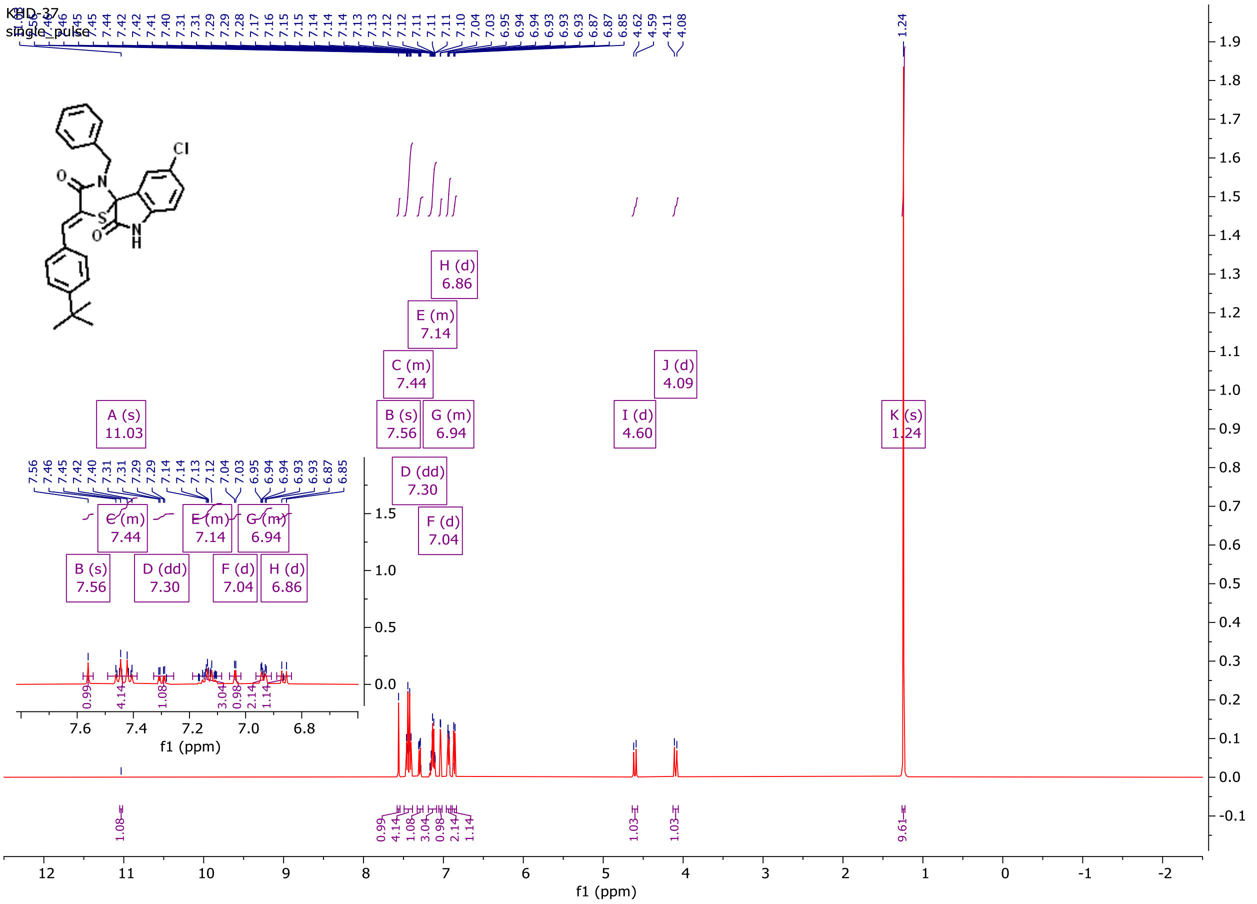

Figure S33. 13C NMR spectrum of compound **17**

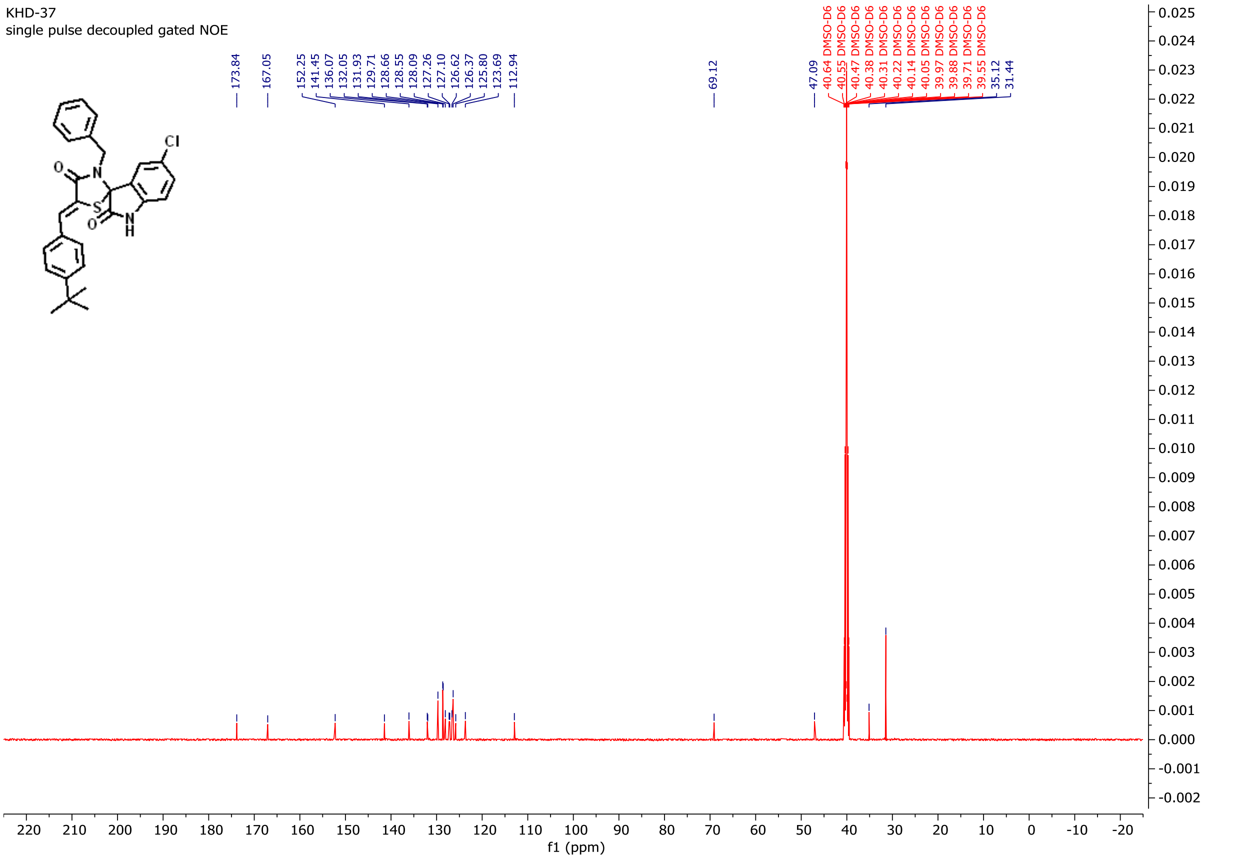

Figure S34. 13C NMR spectrum of compound **17**

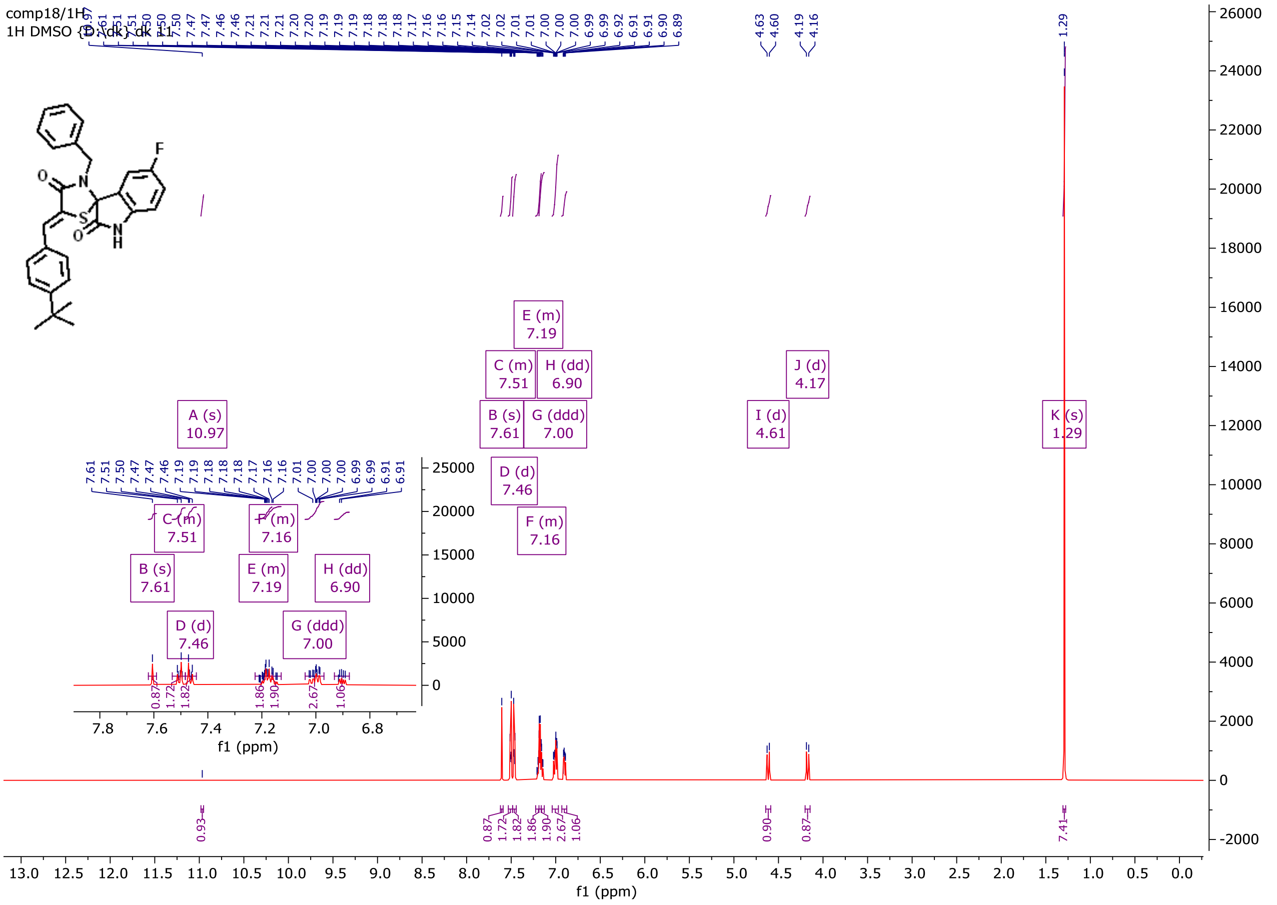

Figure S35. 13C NMR spectrum of compound **18**

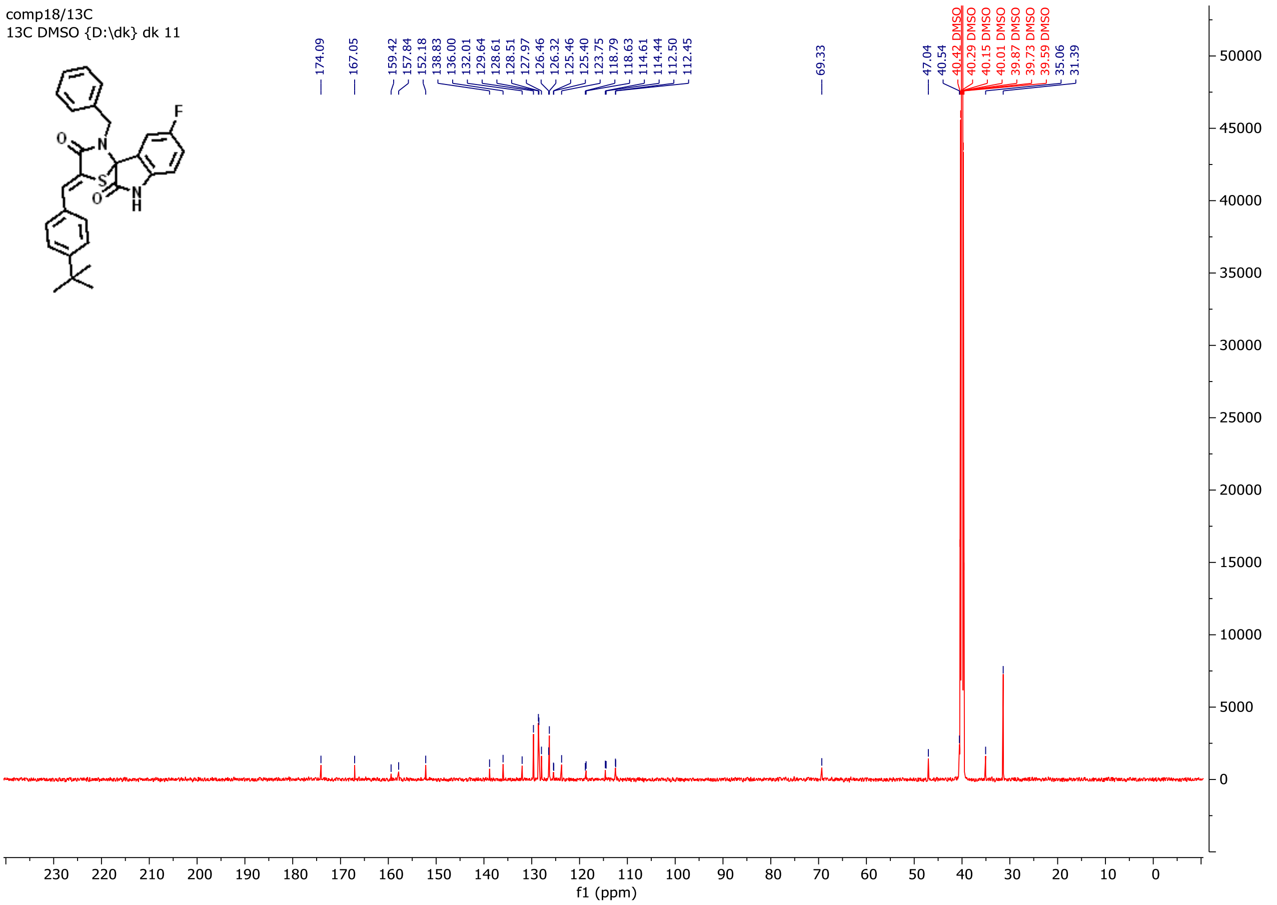

Figure S36. ^{13}C NMR spectrum of compound **18**

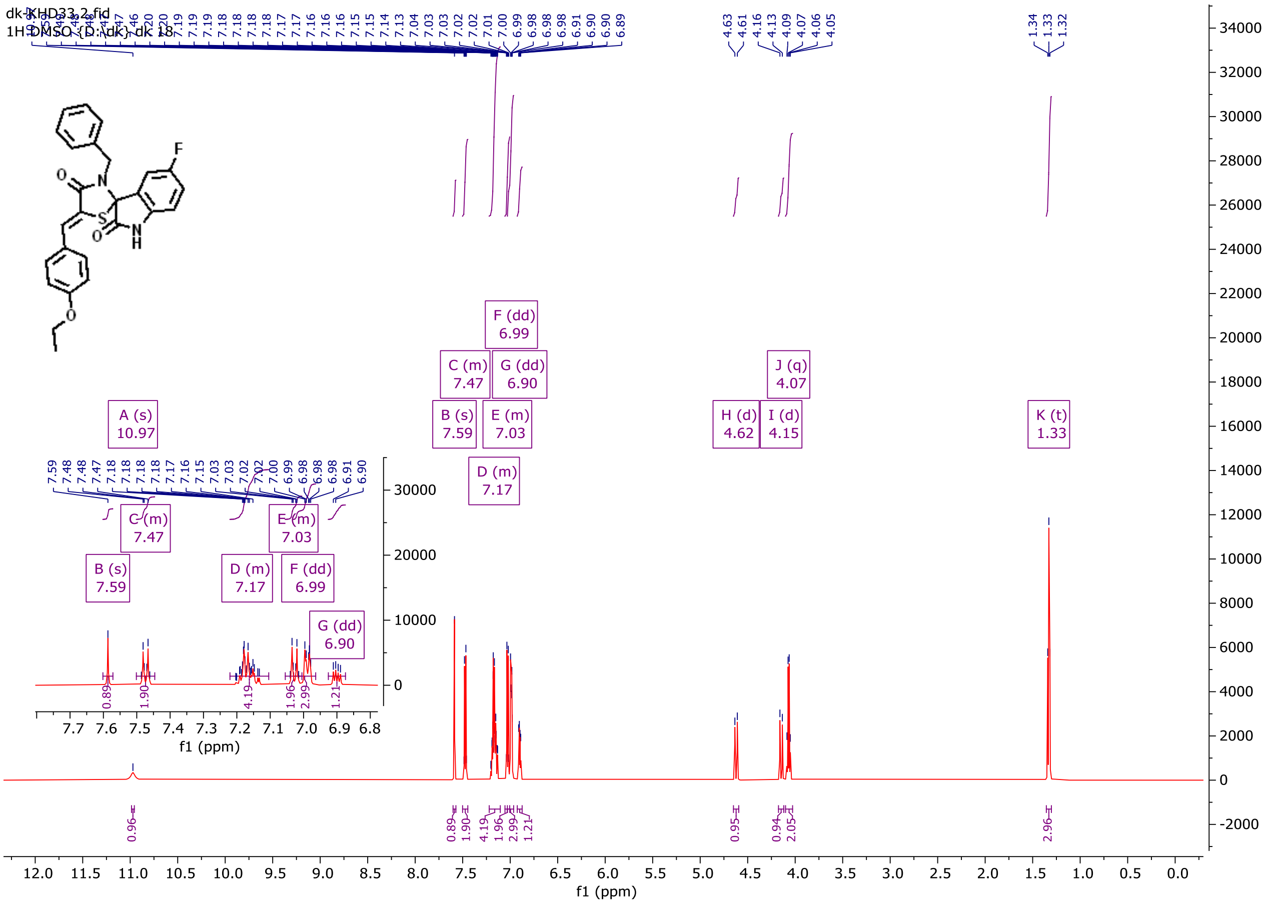

Figure S37. 13C NMR spectrum of compound **19**

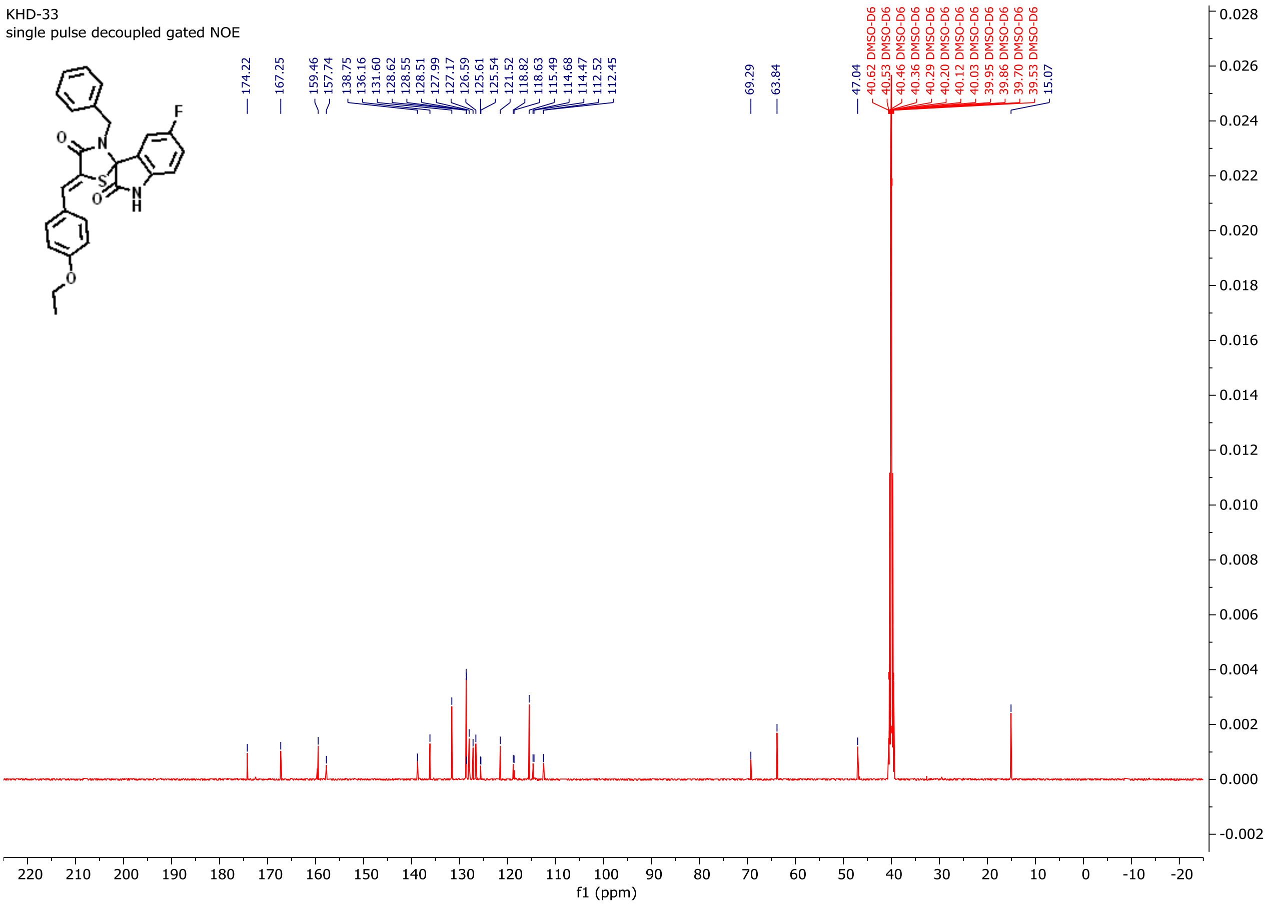

Figure S38. ^{13}C NMR spectrum of compound **19**

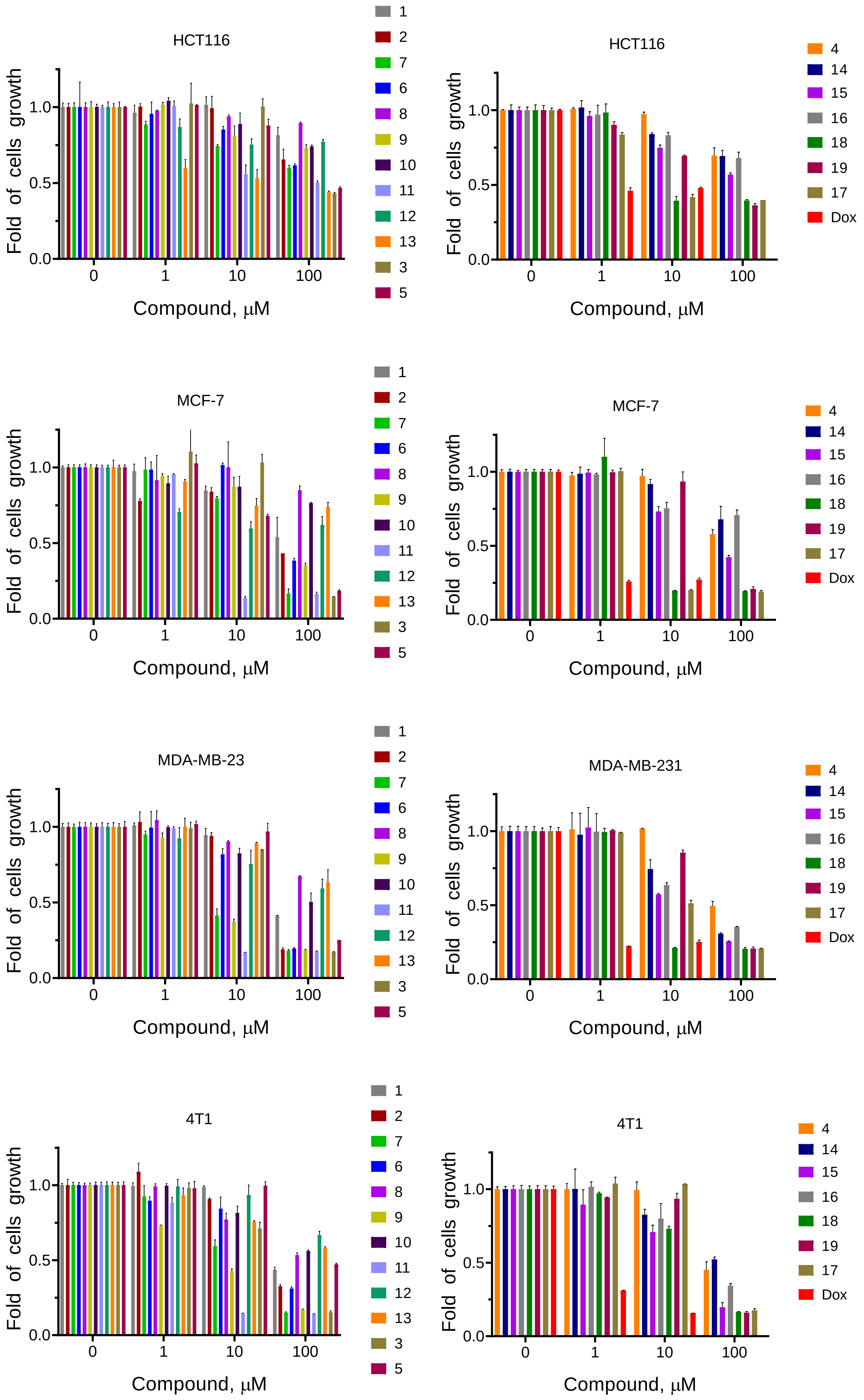
HCT116
Fold of cells growth
1.0
0.5
0.0
0
1
10
100
Compound, μM
1
2
7
6
8
9
10
11
12
13
3
5
HCT116
Fold of cells growth
1.0
0.5
0.0
0
1
10
100
Compound, μM
4
14
15
16
18
19
17
Dox
MCF-7
Fold of cells growth
1.0
0.5
0.0
0
1
10
100
Compound, μM
1
2
7
6
8
9
10
11
12
13
3
5
MCF-7
Fold of cells growth
1.0
0.5
0.0
0
1
10
100
Compound, μM
4
14
15
16
18
19
17
Dox
MDA-MB-23
Fold of cells growth
1.0
0.5
0.0
0
1
10
100
Compound, μM
1
2
7
6
8
9
10
11
12
13
3
5
MDA-MB-231
Fold of cells growth
1.0
0.5
0.0
0
1
10
100
Compound, μM
4
14
15
16
18
19
17
Dox
4T1
Fold of cells growth
1.0
0.5
0.0
0
1
10
100
Compound, μM
1
2
7
6
8
9
10
11
12
13
3
5
4T1
Fold of cells growth
1.0
0.5
0.0
0
1
10
100
Compound, μM
4
14
15
16
18
19
17
Dox

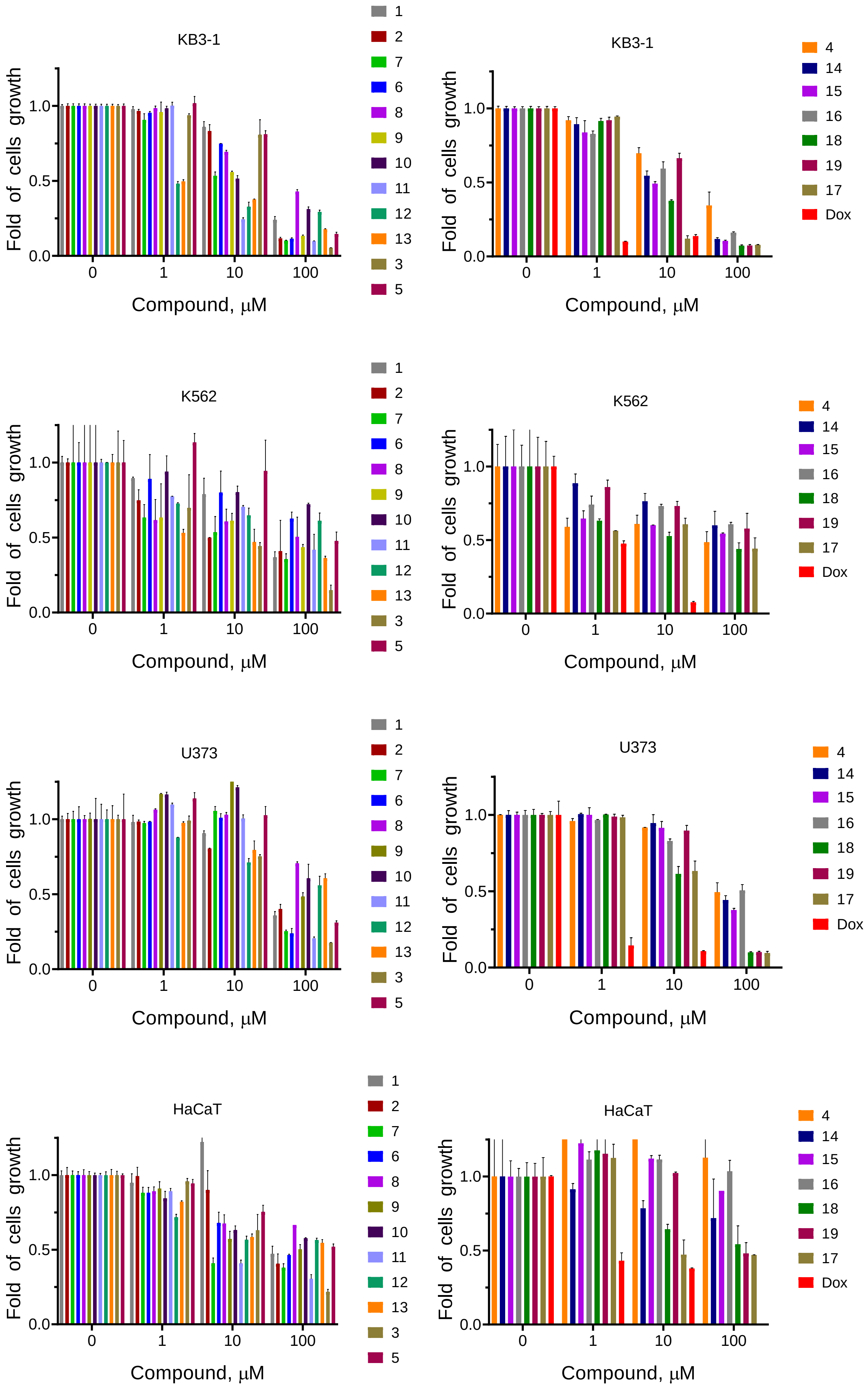
KB3-1
Fold of cells growth
0.0
0.5
1.0
0
1
10
100
Compound, μM
1
2
7
6
8
9
10
11
12
13
3
5
KB3-1
4
14
15
16
18
19
17
Dox
K562
U373
HaCaT

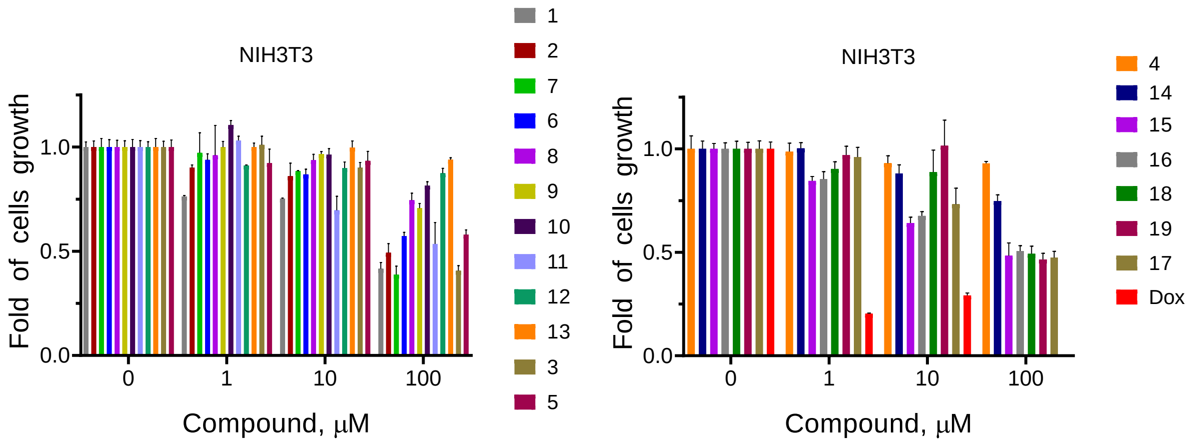

Figure S39. The cytotoxic effects of the investigated spiro-thiazolidinone–isatin conjugates and doxorubicin (Dox) were evaluated in human colorectal carcinoma (HCT116), estrogen receptor-positive breast adenocarcinoma (MCF-7), triple-negative breast adenocarcinoma (MDA-MB-231), epidermoid carcinoma (KB3-1), myeloid leukemia (K562), glioblastoma (U373), mouse breast carcinoma (4T1), as well as pseudo-normal human keratinocytes (HaCaT) and mouse fibroblasts (NIH3T3). Cytotoxicity was assessed by the MTT assay after 72 h of exposure. Data are expressed as mean ± SD.
